# Dental caries experience in Indigenous children and adolescents: a systematic review and meta-analysis assessing worldwide disparities and time trends (1968–2024)

**DOI:** 10.1007/s00784-026-07047-8

**Published:** 2026-07-29

**Authors:** André Luiz Martins, Edmarlon Girotto, Junior César de Souza Benedito, Pablo Guilherme Caldarelli, Kely Barboza Ribeiro, Elis Regina Jacintho, Sara Carolina Scremin Souza, Vicente Martínez-Vizcaíno, Marco Aurelio Anselmo Peres, Arthur Eumann Mesas

**Affiliations:** 1 Postgraduate Program in Public Health, Universidade Estadual de Londrina, Avenida Robert Koch, 60, Londrina, PR 86039-440 Brazil; 2 Indigenous Health Secretariat, Special Indigenous Sanitary District of the Southern Coast, Ministry of Health, Curitiba, Brazil; 3 Ottawa Hospital Research Institute, Ottawa, Canada; 4 Health and Social Research Center, Universidad de Castilla-La Mancha, Cuenca, Spain; 5 Facultad de Ciencias de la Salud, Universidad Autónoma de Chile, Talca, Chile; 6 Institute for Health Research of Castilla-La Mancha (IDISCAM), Toledo, Spain; 7 National Dental Research Institute Singapore, National Dental Centre, Singapore, Singapore; 8 Duke-NUS Medical School, Singapore, Singapore

## Abstract

**Objective:**

To summarize the evidence on the global experience of dental caries among Indigenous children and adolescents worldwide using decayed, missing, and filled teeth indices for deciduous (dmft) and permanent (DMFT) dentitions.

**Materials and methods:**

A systematic review of the PubMed, SciELO, Embase, and Scopus databases was conducted. The search included studies with primary data on dmft and DMFT in Indigenous populations worldwide published until February 9, 2026. The pooled means (95% CI) of dmft and DMFT were calculated using a random-effects meta-analysis. Subgroup analyses and meta-regression explored heterogeneity through sociodemographic, geographic and time variables.

**Results:**

A total of 95 studies from 19 countries were included in the systematic review, 59 of which provided data for meta-analysis. The pooled mean for dmft was 3.75 (3.58–3.92) and for DMFT was 2.36 (2.18–2.55). Considerable heterogeneity was detected in pooled analyses for dmft and DMFT. Both indices were higher in studies from North America (dmft = 5.23 [4.61–5.86]; DMFT = 4.74 [2.28–7.20]) than in those from Oceania and Asia (dmft = 3.30 [3.11–3.50]; DMFT = 1.74 [1.57–1.91]). Multivariate meta-regression revealed that dmft increased as the percentage of female participants rose (β = 0.10, *p*-value = 0.030). Additionally, DMFT increased with increasing age (β = 0.40, *p*-value < 0.001) and decreased with increasing HDI (β=-3.13, *p*-value = 0.034). No statistically significant differences in caries experience were found across the study periods.

**Conclusions:**

For nearly six decades, Indigenous children and adolescents worldwide have consistently experienced dental caries affecting approximately two to four teeth. Disparities in dental caries were observed across age, sex, and national human development levels.

**Clinical relevance:**

The persistence of dental caries underscores the urgency of implementing effective preventive strategies that are culturally adapted to Indigenous contexts.

**Supplementary Information:**

The online version contains supplementary material available at 10.1007/s00784-026-07047-8.

## Introduction

Indigenous populations are characterized by a rich cultural, linguistic and spiritual diversity that is manifested in their ways of life, traditional health practices, ancestral knowledge and balanced connections with the environment [1, 2]. The United Nations Declaration on the Rights of Indigenous Peoples (UNDRIP) guarantees self-determination, the right to traditional lands and resources, and prior consultation and protection of their languages, cultures and knowledge, ensuring their full and equal participation in society [3]. Despite their notable sociocultural and geographical differences, Indigenous populations share a common struggle to protect their rights because of contact and a prolonged history of colonization and structural and interpersonal racism [4, 5]. Indigenous sovereignty has been increasingly strained by the escalating impacts of global environmental change within their territories, including climate change and pollution, alongside persistent threats to and infringements upon Indigenous land and water rights [6]. This process has led to substantial changes in various sociocultural and economic systems and has affected lifestyles, demographics, and health conditions worldwide [1, 4, 7]. As efforts to document and address these health impacts expand, the production, use, and governance of health data concerning Indigenous peoples also raise important questions of Indigenous data sovereignty. Grounded in the principle of self-determination and reflected in frameworks such as the UNDRIP and the CARE Principles (Collective Benefit, Authority to Control, Responsibility, and Ethics), Indigenous data sovereignty must be considered when situating Indigenous oral health within a global public health perspective [8, 9].

Health disparities are frequently observed in scientific evidence comparing Indigenous and non-Indigenous populations [10–12]. In areas with greater interaction with the urban population, substantial changes in lifestyle and higher rates of chronic conditions, such as hypertension, diabetes, depression, and notably, dental health problems, have been reported [13–15]. Epidemiological changes in oral health conditions result from a complex interplay among social, economic, commercial [16–19], cultural [20], and environmental determinants [21], which disproportionately affect more vulnerable groups [20]. Among Indigenous populations, this process may assume distinct forms across different ethnic groups [21]. Indigenous populations face limited access to oral health promotion initiatives and services [10, 22] and suffer from poorer oral health indicators, including higher rates of dental caries (tooth decay), than non-Indigenous populations [15]. Untreated dental caries can be up to three times more prevalent among Indigenous adults than among non-Indigenous adults [23]. Comparable disparities were also observed between Indigenous and non-Indigenous children [15] suggesting that the onset of these inequalities may occur at early stages of life and persist into adulthood.

In addition to individual factors, such as ethnicity, age, and sex, the prevalence and experience of dental caries are also influenced by contextual factors affecting lifestyle and oral health policies and programs, including the country or global region of residence and its socioeconomic conditions and level of human development [24]. In contrast to improvements among non-Indigenous children in Latin American countries, there has been a significant increase in dental caries among Indigenous children in recent decades [25]. Similarly, the prevalence of early childhood caries is greater among Indigenous than non-Indigenous populations in developed countries such as the United States, Canada, Australia, and New Zealand [26].

While these findings suggest that such dental health disparities occur regardless of national wealth or development status, there is a lack of global data on the experience of childhood dental caries in Indigenous populations across ethnic groups, global regions, and time periods. These data are critical for developing tailored preventive strategies and early treatment to address dental health inequities among the Indigenous population. The gold standard indices used to characterize the experience of dental caries across different populations are the decayed, missing due to caries, and filled teeth (dmft) index for deciduous (primary) dentition and the DMFT index for permanent dentition, both of which are based on World Health Organization (WHO) criteria [27]. In both indices, each point represents a tooth that has experienced dental caries.

Previous systematic reviews have investigated inequities in the prevalence and experience of dental caries by comparing rates between Indigenous and non-Indigenous populations [15, 23, 28, 29] and dental caries among Indigenous populations in specific regions of the world [30, 31]. However, to the best of our knowledge, this is the first systematic review to comprehensively assess the global experience of dental caries in Indigenous children and adolescents or its potential associations with individual and contextual factors over time.

Therefore, we aimed to synthesize the global evidence on the experience of dental caries in Indigenous children and adolescents as measured by the dmft and DMFT indices. Additionally, subgroup analyses and meta-regression were employed to examine whether these indices vary according to individual factors (mean age and percentage of female individuals among study participants), contextual factors (country, global region and the Human Development Index [HDI]), and the time trend based on the year the study data were collected.

## Methods

### Protocol and registration

This systematic review was conducted in accordance with the Preferred Reporting Items for Systematic Reviews and Meta-Analyses (PRISMA) and Meta-analysis Of Observational Studies in Epidemiology guidelines (MOOSE) [32, 33]. The review protocol was prospectively registered with the International Prospective Register of Systematic Reviews (PROSPERO) under registration number CRD42024590381.

### Search strategy and databases

A systematic search was conducted in the following electronic databases from the date of inception to March 9, 2025: MEDLINE (via PubMed), Embase, Scopus and SciELO. The search was subsequently updated to include the period from March 10, 2025, to February 9, 2026. The search strategy combined keywords related to dental caries and Indigenous populations. The search terms used with the appropriate Boolean operators (AND or OR) included “dental caries”, “Indigenous”, and “children and adolescents” in addition to several synonymous and related terms. Complete search strategies with database-specific syntax are provided in the supplementary material (Table S1). No publication year, language, sex, age, or geographical location restrictions were applied.

### Eligibility criteria

The research question was formulated using the population, exposure, and outcome framework adapted for observational studies: population (Indigenous children and adolescents), exposure (Indigenous ethnicity/cultural background), and outcome (dental caries experience measured by dmft/DMFT indices). This framework guided the study selection and data extraction protocols.

The inclusion criteria were as follows: (1) observational study designs, including cross-sectional, cohort, or case‒control studies, that reported dental caries data in Indigenous populations; (2) participants aged 0–19 years (this range was selected to include both the developmental periods of deciduous and permanent dentition and to allow for a comprehensive assessment of caries across childhood and adolescence); (3) studies that reported caries experience using the standardized dmft index for deciduous dentition or the DMFT index for permanent dentition; and (4) studies from any geographical region, provided that they included clearly defined Indigenous populations with explicit ethnic or cultural identification.

The exclusion criteria were as follows: (1) studies that did not address dental caries in Indigenous populations as the primary outcome; (2) nonprimary research articles, including systematic reviews, meta-analyses, narrative reviews, editorials, commentaries, conference abstracts, and case reports; (3) studies using non-standardized caries indices or composite measures that could not be disaggregated into dmft or DMFT values; (4) mixed-population studies that included both Indigenous and non-Indigenous participants, unless they provided stratified results by ethnicity or Indigenous status; (5) studies reporting aggregate data across dentition types that combine deciduous and permanent teeth without providing individual dmft and DMFT indices; (6) intervention studies, including randomized controlled trials and quasiexperimental designs, as the research focus was on observational prevalence data rather than treatment effects; (7) studies that included participants outside the 5–19 years of age range, unless age-stratified reporting allowed extraction of data for the target age group; (8) duplicate publications reporting identical datasets (preference was given to the most recent or methodologically comprehensive version); and (9) no data of interest.

### Study identification and selection

Two PhD candidates independently screened all retrieved articles by title and abstract, followed by a full-text review of potentially eligible studies. Disagreements between reviewers were resolved through consensus or consultation with a third researcher (PhD Advisor). Studies meeting the inclusion criteria were subjected to data extraction, whereas ineligible studies were excluded for documented reasons following the PRISMA and MOOSE guidelines.

### Data extraction

Using a standardized extraction form, researchers collected key study variables, including publication details, geographical location, the Indigenous populations included, population characteristics, study design, outcome measures, and dental caries experience data (dmft/DMFT values with statistical measures). Data extraction was performed independently by two reviewers, with discrepancies resolved through discussion or third-party consultation.

### Evaluation of methodological quality

Two researchers independently evaluated the methodological quality of the included studies via the Joanna Briggs Institute critical appraisal checklist [34]. Six of the eight checklist items were applied; “exposure” and “results” items were deemed inapplicable to the study objectives and design. Thus, the remaining six items were classified as “yes”, “no”, or “unclear”. Any disagreement between evaluators on quality assessments was resolved through structured discussion, with unresolved conflicts adjudicated by a third independent reviewer to ensure consistent and reliable quality evaluation across all included studies.

### Statistical analysis

A statistical meta-analysis was conducted on studies reporting mean dmft and DMFT indices and corresponding sample sizes and measures of dispersion, such as standard deviation, standard error, confidence interval, or variance. When studies presented results stratified by variables such as ethnicity, sex, age group, socioeconomic status, or geographic location [35–61], pooled means were calculated using an online statistical tool (https://home.ubalt.edu/ntsbarsh/business-stat/other_applet/Pooled.htm). When studies reported results for multiple years (repeated cross-sections) [36, 38, 50, 62, 63], each year was treated as an independent analytical unit. For two studies [35, 63], graphical data were digitized using WebPlotDigitizer software (http://apps.automeris.io/wpd4/). For one study [35], sample size estimation was performed using Australian population census data [64]. For the remaining studies with missing data [35, 65–68], the authors were directly contacted via email, and one responded [66].

For the meta-analysis, the studies reporting dmft included participants aged 0–13 years. Studies reporting DMFT were stratified into two groups: children (5–12 years) and adolescents (13–19 years), and systematically categorized by multiple variables, including country of origin (single-study countries were grouped as “others”), global region (North America, Latin America, Asia, and Oceania), age group (based on the participants’ mean age), and sex distribution. The age categorization used mean ages, and the sex distribution was categorized by the percentage of female participants. We use “sex” rather than “gender” because most studies reported only binary male/female categories. However, we acknowledge that dental health and caries are influenced by gender-related factors (e.g., self-care and women´s roles in Indigenous culture) that future research should address [69].

The temporal analysis grouped studies by data collection period. For studies spanning multiple years, the mean collection year was calculated, and non-integer results were rounded down to the preceding year. For studies without reported collection dates, the year before publication was used as the reference point. The HDI values were extracted from the 2023/24 Human Development Report by the United Nations Development Programme [70], which covered the same years as the studies for which HDI data was available. The closest available year was used if an exact match was not possible. The HDI categorization differed by dental caries experience index to ensure that an adequate number of studies were included in each group.

Meta-regression analyses were used to examine the relationships between the dmft/DMFT indices and the following key potential moderator variables: mean age, percentage of female individuals, global region, HDI, and year of data collection. Pearson correlation tests were used to assess the associations between effect sizes and study characteristics. Heterogeneity was assessed using the I² statistic with the following categorization: unimportant (0%-40%), moderate (30%-60%), substantial (50%-90%), and considerable (75%-100%) [71], and the corresponding *p*-values were analyzed particularly for overlapping ranges. Sensitivity analyses evaluated the robustness of the results by systematically removing individual studies to identify those with disproportionate contributions to heterogeneity [72]. The pooled estimates were then recalculated with 95% confidence intervals (CIs) [72]. Publication bias was assessed using a visual inspection of the funnel plot and Egger regression test to detect small-study effects and potential reporting bias [72].

## Results

### Study selection

The complete search identified 19,129 registries. After excluding duplicates, a total of 13,756 studies remained for screening. Following a review of the title and abstract, 169 studies were selected. Six additional studies were identified through complementary searches, resulting in a total of 175 studies selected for full-text evaluation. When the exclusion criteria were applied (Table S2), 95 studies [23, 35–53, 55–63, 65–68, 73–133] met the eligibility requirements for inclusion in the systematic review, 59 of which were included in the meta-analyses [23, 35–53, 55–63, 65–68, 73–109, 111–130, 134]. Specifically, 45 studies [23, 35–40, 43, 44, 46–50, 52, 53, 55, 60–63, 74, 75, 77, 79, 80, 87, 90–95, 98, 105, 108, 109, 113, 114, 120, 126–128, 130] provided 64 independent data points valid for the dmft index, whereas 39 studies [35, 36, 38–49, 51, 53, 56–59, 62, 63, 74, 75, 79, 90–94, 98, 102, 103, 106, 109, 112, 113, 117, 122] provided 71 independent data points valid for the DMFT index.

**Fig. 1:**
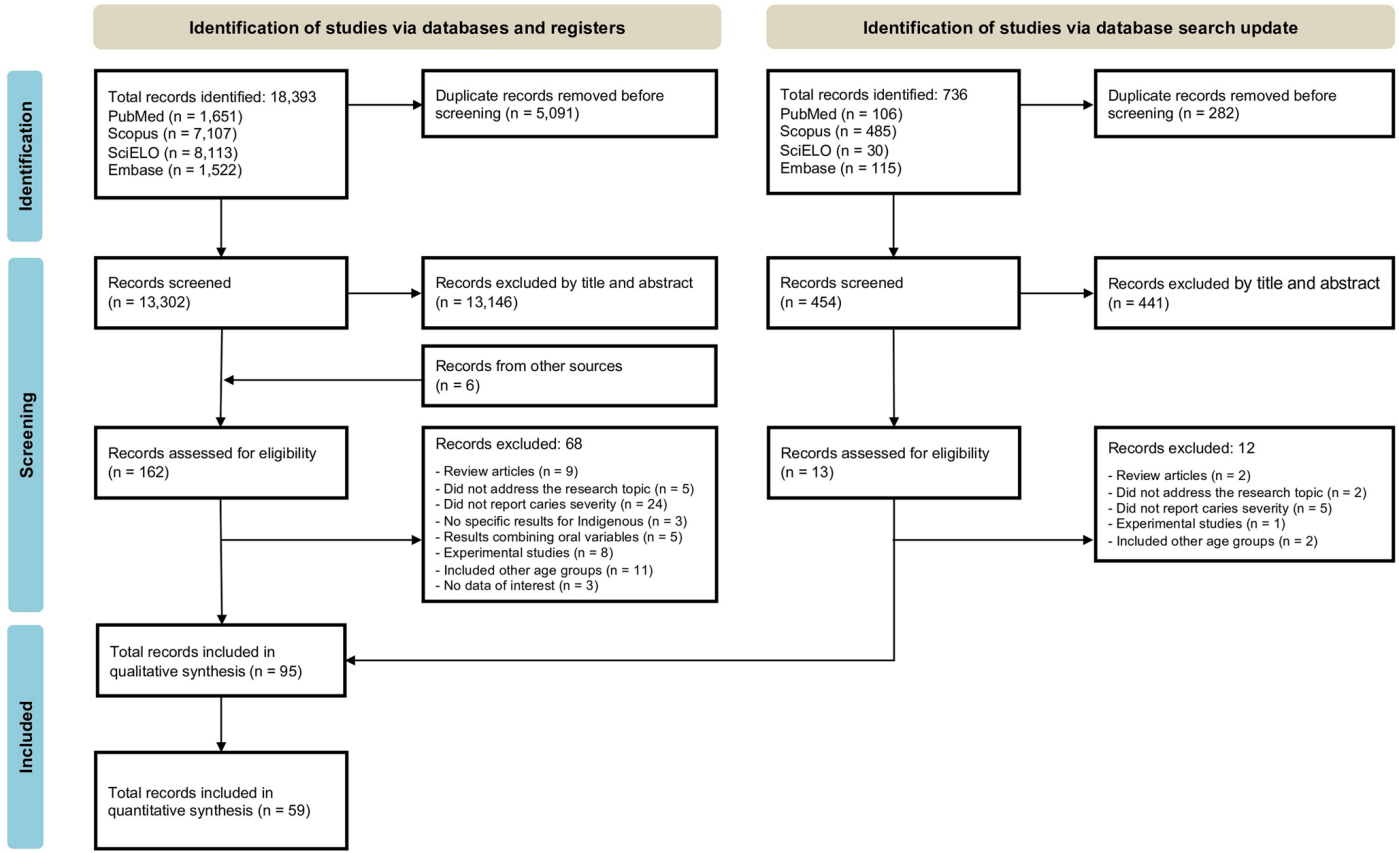
PRISMA flow diagram of study identification and selection

The PRISMA flow diagram shows the detailed study identification and selection process (Fig. 1).

### Characteristics of the included studies

The 95 included studies included Indigenous populations from 19 countries. The largest representation was from Australia (*n* = 32) [35, 38, 42, 43, 47, 51, 59, 62, 63, 67, 74, 77, 83, 86, 87, 89, 93, 95, 97–102, 104, 108, 118, 120, 121, 127, 129, 134], followed by Brazil (*n* = 22) [36, 37, 39–41, 44–46, 48, 49, 52, 53, 56, 60, 66, 78, 88, 90, 96, 107, 111, 135], Canada (*n* = 9) [50, 55, 61, 113, 114, 123, 128, 133, 134], the United States (*n* = 6) [23, 57, 75, 92, 105, 124], New Zealand (*n* = 4) [81, 91, 119, 134], China (*n* = 4) [58, 79, 122, 130], Ecuador (*n* = 3) [65, 80, 112], India (*n* = 3) [103, 109, 126], Malaysia (*n* = 3) [73, 76, 106] and Sri Lanka (*n* = 2) [68, 82]. Some countries were represented by a single study, including Mexico [85], Argentina [84], Cameroon [115], Nepal [116], Panama [94], Venezuela [117], Tanzania [125], Paraguay [131] and Chile [132]. Most of the included studies were cross-sectional (*n* = 91, 96.8%), and only three studies (3.2%) were longitudinal [45, 79, 98] (Table 1).

**Table 1:** Characteristics of the studies on dental caries in Indigenous populations included in the systematic review

Authors (year of publication)	Year of data collection	Country	Study design	Sample size (only Indigenous individuals)	Ethnicity/Indigenous populations
Abdul Kadir, Adnan (1989)	NR	Malaysia	Cross-sectional	69	Orang Asli
Alves Filho, Santos and Vettore (2009)	2006	Brazil	Cross-sectional	508	Guarani
Arantes & Frazão (2016)	2013	Brazil	Cross-sectional	509	Kaiwoá, Guarani, and Terena
Arantes et al. (2009)	1999/2004	Brazil	Longitudinal	128	Xavante
Arantes, Jamieson and Frazão (2021)	2010, 2012–2014	Brazil	Cross-sectional	1,830	Guarani, Kaiwoá, Terena, Kadiwéu
Arantes, Santos and Coimbra Jr. (2001)	1997	Brazil	Cross-sectional	228	Xavante
Arrow (2016)	2014	Australia	Cross-sectional	268 for deciduous dentition; 349 for permanent dentition	Aboriginal and Torres Strait Islander
Baldisserotto, Ferreira and Warmling (2019)	2009–2010	Brazil	Cross-sectional	204	Guarani (Ñandeva, Mbyá, and Kaiowá)
Barrett et al. (1972)	1970	Australia	Cross-sectional	309	Aboriginal
Batliner et al. (2016)	NR	United States	Cross-sectional	399	Native Americans
Berhan Nordin et al. (2019)	2014	Malaysia	Cross-sectional	227	Orang Asli
Butten et al. (2019)	2013–2015	Australia	Cross-sectional	180	Aboriginal and Torres Strait Islander
Caires et al. (2018)	2017	Brazil	Cross-sectional	138	Sateré-Mawé and Tikuna
Carneiro et al. (2008)	2004	Brazil	Cross-sectional	590	Baniwa
Chang (1971)	1966–1970	China	Longitudinal	90	Aboriginal
Chinnakotla et al. (2023)	2011–2013	Ecuador	Cross-sectional	458	Indigenous Peoples
D’Mello et al. (2011)	2004–2006	New Zealand	Cross-sectional	31	Māori
Dasanayake and Caufield (2002)	1997	Sri Lanka	Cross-sectional	39	Veddas
Davies et al. (1997)	1992	Australia	Cross-sectional	429 for deciduous dentition; 407 for permanent dentition	Aboriginal
De la Maza, Cueto (1989)	1987	Chile	Cross-sectional	200	Mapuche
de Muñiz (1985)	NR	Argentina	Cross-sectional	135 for deciduous dentition; 362 for permanent dentition	Amerindians
del Rio Gomez (1991)	NR	Mexico	Cross-sectional	100	Mazahua
Dimitropoulos et al. (2018)	2014	Australia	Cross-sectional	88	Aboriginal
Dogar et al. (2011)	NR	Australia	Cross-sectional	79	Aboriginal
Drummond et al. (2015)	2010	Brazil	Cross-sectional	48	Indigenous people in an urban context
Endean et al. (2004)	1999–2000	Australia	Cross-sectional	606	Anangu
Fischman (1974)	1965	Paraguay	Cross-sectional	107	Chaco indians
Gonçalves et al. (2015)	2011	Brazil	Cross-sectional	342	Xukuru
Gowda et al. (2009)	NR	New Zealand	Cross-sectional	236	Māori
Grim et al. (1994)	1989	United States	Cross-sectional	457	Native Americans
Guisilini et al. (2021)	2012–2013	Brazil	Cross-sectional	402	Indigenous Peoples of the Lower, Middle, and East Xingu
Ha (2014)	2010	Australia	Cross-sectional	7,031	Aboriginal, Torres Strait Islander, South Sea Islander
Ha et al. (2016)	2000–2002/2007–2010	Australia	Cross-sectional	16,592 (2002); 8,354 (2010)	Aboriginal, Torres Strait Islander, South Sea Islander
Ha, Crocombe and Mejia (2014)	2009	Australia	Cross-sectional	2,918 for deciduous dentition 2,311 for permanent dentition	Aboriginal, Torres Strait Islander, South Sea Islander
Hagens et al. (2023)	2022	Panama	Cross-sectional	106	Ngäbe-Buglé
Hallett and O’Rourke (2002)	2000	Australia	Cross-sectional	48	Aboriginal and Torres Strait Islander
Hirata et al. (1977)	NR	Brazil	Cross-sectional	210	Camaiurá, Iuaulapiti, Uaurá, Calapalo, Cuicuro, Menaico, Matipu, Nafuqua, Txição, and Trumai
Hirooka et al. (2014)	2007	Brazil	Cross-sectional	246 (mothers); 206 (children)	Indigenous Peoples of the Middle and Lower Xingu
Homan and Davies (1973)	NR	Australia	Cross-sectional	307	Aboriginal and Torres Strait Islander
Hopcraft and Chow (2007)	2004	Australia	Cross-sectional	486	Aboriginal and Torres Strait Islander
Jamieson et al. (2010)	1993–2010	Australia	Longitudinal	145	Aboriginal and Torres Strait Islander
Jamieson et al. (2013)	2006–2008	Australia	Cross-sectional	442	Aboriginal and Torres Strait Islander
Jamieson et al. (2016)	2004–2009	Australia, Canada, New Zealand	Cross-sectional	65 Australians; 105 Canadians; 386 New Zealanders	Aboriginal, Torres Strait Islander, Māori
Jamieson et al. (2021)	2004–2006/2017–2018	Australia	Cross-sectional	229 (2004–2006); 334 (2017–2018)	Aboriginal and/or Torres Strait Islander
Jamieson, Armfield and Roberts-Thomson (2006)	2000–2003	Australia	Cross-sectional	10,473	Aboriginal and Torres Strait Islander
Jamieson, Armfield and Roberts-Thomson (2007)	2000–2003	Australia	Cross-sectional	10,517	Aboriginal and Torres Strait Islander
Jamieson, Armfield and Roberts-Thomson (2007)	1989–2000	Australia	Cross-sectional	196 (1989); 547 (2000)	Aboriginal
Jamieson, Roberts-Thomson and Sayers (2010)	2005–2008	Australia	Cross-sectional	442	Aboriginal and Torres Strait Islander
Jamieson, Sayers and Roberts-Thomson (2013)	2003–2010	Australia	Cross-sectional	441	Aboriginal
Jayashantha and Johnson (2016)	2012	Sri Lanka	Cross-sectional	194	Veddas
John et al. (2015)	NR	India	Cross-sectional	206	Tribal
Johnson et al. (2014)	2012	Australia	Cross-sectional	324	Aboriginal
Jones et al. (1992)	NR	United States	Cross-sectional	381	Alaska Natives
Kadir et al. (1990)	NR	Malaysia	Cross-sectional	303	Orang Asli
Kailis (1979)	1971	Australia	Cross-sectional	372	Aboriginal
Koike et al. (2024)	2016	Brazil	Cross-sectional	104	Fulni-ô
Kroon et al. (2019)	2004–2017	Australia	Cross-sectional	393 (2004); 263 (2012); 284 (2015); 87 (2016); 63 (2017)	Aboriginal and Torres Strait Islander
Kruger, Dyson and Tennant (2005)	NR	Australia	Cross-sectional	31	Aboriginal
Kumar et al. (2013)	NR	India	Cross-sectional	743	Tribal
Lalloo et al. (2016)	2010	Australia	Cross-sectional	6,825	Aboriginal, Torres Strait Islander, South Sea Islander
Lawrence et al. (2009)	2001–2005	Canada	Cross-sectional	460 (2001); 699 (2002); 1,275 (2003); 974 (2004); 952 (2005)	First Nations
Lee et al. (2022)	2018–2019	Canada	Cross-sectional	146	First Nations and Métis
Lemos et al. (2018)	2007, 2013	Brazil	Cross-sectional	368 (2007); 423 (2013)	Kisêdje, Ikpeng, Kaiabi, Trumai, Kamayura, Yudjá, Waurá, Tapayuna
Maurício and Moreira (2014)	2010	Brazil	Cross-sectional	233	Xukuru do Ororubá
Maurício, Fávaro and Moreira (2024)	2018	Brazil	Cross-sectional	131	Xukuru do Ororubá
Medina et al. (2008)	1998	Ecuador	Cross-sectional	930	Naporunas
Miranda, Souza and Leal (2018)	2010	Brazil	Cross-sectional	308	Indigenous people in an urban context
Miyazaki and Takehara (1988)	1979–1980	China	Cross-sectional	843	Aboriginal
Nascimento, Scabar (2008)	NR	Brazil	Cross-sectional	77	Wai-wai
Niendorff and Jones (2000)	1991	United States	Cross-sectional	24,696 (permanent dentition); 12,518 (deciduous dentition)	Native Americans
Oliveira et al. (2024)	NR	Brazil	Cross-sectional	125	Parakanã
Peressini et al. (2004)	2000	Canada	Cross-sectional	66	First Nations
Peressini et al. (2004)	2000	Canada	Cross-sectional	87	First Nations
Phipps et al. (2012)	2010–2011	United States	Cross-sectional	8,461	Native Americans and Alaska Natives
Poni et al. (2023)	2017	Cameroon	Cross-sectional	120	Baka Indigenous Peoples
Prasai Dixit et al. (2013)	NR	Nepal	Cross-sectional	361	Chepang
Quintero De La Hoz (2022)	2016	Venezuela	Cross-sectional	25	Indigenous Peoples of Maracaibo
Rigonatto, Antunes and Frazão (2001)	1991	Brazil	Cross-sectional	288	Yawalapiti, Aweti, Mehinaku, Kamaiura
Sampaio et al. (2010)	2003	Brazil	Cross-sectional	1,461	Potiguara
Schamschula et al. (1980)	NR	Australia	Cross-sectional	83	Aboriginal
Schluter, Lee (2016)	2004–2013	New Zealand	Cross-sectional	93,715 (5 years) 94,001 (8 years)	Māori
Schroth, Moore and Brothwell (2005)	2001–2022	Canada	Cross-sectional	236	First Nations
Seow et al. (1996)	NR	Australia	Cross-sectional	184	Aboriginal and Torres Strait Islander
Seow et al. (1999)	1996–1997	Australia	Cross-sectional	147	Aboriginal
Shen et al. (2015)	2005	China	Cross-sectional	360	Non-Han
Shi et al. (2018)	2013–2014	Canada	Cross-sectional	129	First Nations, Métis, Inuit
Ship (1966)	1959–1962	United States	Cross-sectional	6,988	Indigenous Peoples
Simangwa et al. (2018)	2016	Tanzania	Cross-sectional	721	Maasai
Singh et al. (2011)	NR	India	Cross-sectional	418	Aboriginal
Smith et al. (2015)	2014	Australia	Cross-sectional	173	Aboriginal and Torres Strait Islander
So et al. (2017)	2011–2013	Ecuador	Cross-sectional	1,407	Kichwa
Tsai and Lawrence (2022)	2011–2015	Canada	Cross-sectional	344	First Nations
Yerex et al. (2025)	2006 2016	Canada	Cross-sectional	4,773 7,442	First Nations and Inuit
Zander et al. (2013)	2011	Australia	Cross-sectional	138	Aboriginal
Zeng et al. (2005)	NR	China	Cross-sectional	470	Zhuang

*NR* not reported

A total of 60 studies (62.8%) focused on deciduous dentition using the dmft index [23, 35–40, 43, 44, 46–48, 50, 52, 53, 55, 57, 60–63, 65, 67, 74, 75, 77–83, 86, 87, 89–95, 98, 100, 101, 104, 105, 108, 109, 113–115, 119, 120, 123, 126–130, 133], while 67 studies (71.3%) examined permanent teeth using the DMFT index [35, 36, 38–49, 51, 53, 56–60, 62, 63, 66–68, 73–76, 78, 79, 82, 83, 85, 86, 89–94, 97–104, 106, 109, 111–113, 115–119, 122–124, 129, 131, 132, 135].

Australia was the leading contributor, with the highest number of studies in both indices, 23 studies for dmft [35, 38, 43, 47, 62, 63, 67, 74, 77, 83, 86, 87, 89, 93, 95, 98, 100, 101, 104, 108, 120, 127, 129], and 24 for DMFT [35, 38, 42, 43, 47, 51, 59, 62, 63, 67, 74, 83, 86, 89, 93, 97–102, 104, 118, 129], followed by Brazil with 12 [36, 37, 39, 40, 44, 46, 48, 52, 53, 60, 78, 90] and 17 [36, 39–41, 44–46, 48, 49, 53, 56, 60, 66, 78, 90, 111, 135] studies, respectively (Table 1).

### Evaluation of methodological quality

Overall, most assessments fully met the methodological requirements with “yes” ratings. However, methodological deficiencies were identified in approximately one-fifth of the assessments, of which half received “no” ratings and half were classified as “unclear” due to insufficient reporting detail (Table S3). In summary, the most significant methodological concerns centered on two specific criteria: a detailed description of the study subjects and settings showed inadequacy in 19.9% of the studies, whereas the use of objective measurement criteria demonstrated deficiencies in 25.3% of the assessments (Table S4). Additional areas of uncertainty included definitions of inclusion criteria (7.4% “unclear”) and strategies for addressing confounding variables (14.7% “unclear”). All studies adequately addressed confounder identification and statistical analysis methodology (100% “yes” ratings), suggesting strong analytical approaches despite some descriptive limitations (Table S4).

### Meta-analyses results

#### Deciduous dentition (dmft)

The meta-analysis of deciduous dentition caries included 44 studies reporting dmft indices across multiple geographic regions and Indigenous populations [23, 35–40, 43, 44, 46–50, 52, 53, 55, 60–63, 74, 75, 77, 79, 80, 87, 90–95, 98, 105, 108, 109, 113, 114, 120, 126–128, 130]. The overall pooled estimate revealed a global mean dmft of 3.75 (95% CI: 3.58–3.92) among Indigenous children. Heterogeneity was considerable across all included studies (I²=98.7%) (Figure S1).

**Fig. 2:**
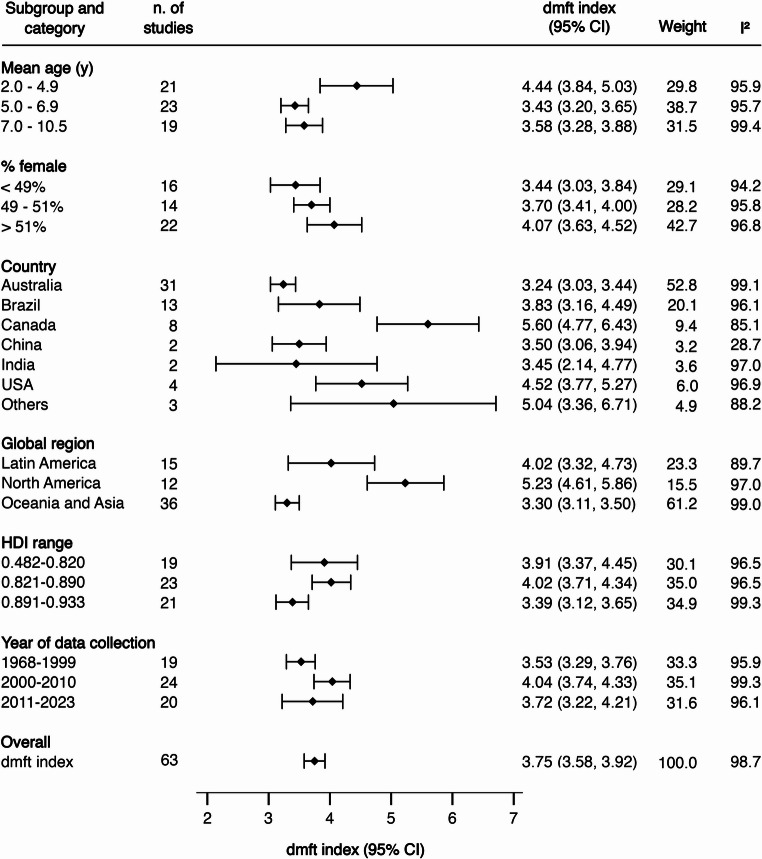
Graphical representation of the dmft index of Indigenous children by subgroup of mean age, percentage of female individuals, country, global region, HDI range, and data collection period and overall data (dmft: decayed, missing due to caries, and filled teeth index for deciduous dentition; HDI: human development index)

**Fig. 3:**
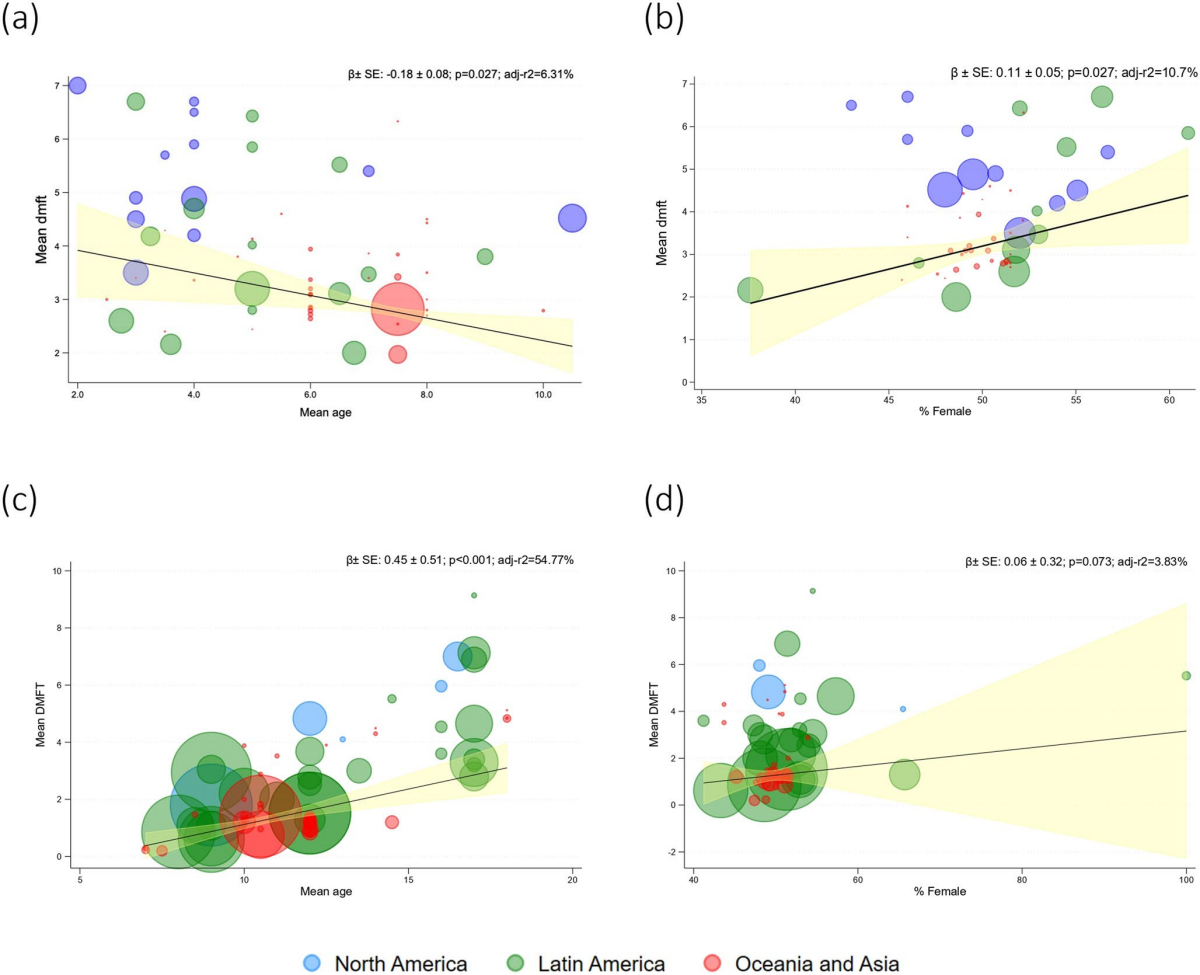
Meta-regression of the mean dmft and DMFT indices according to the mean age and percentage of female individuals (The colors indicate the global region, and the circle sizes represent the weight of the studies)

When the associations between dmft and age groups were examined, younger children (i.e., aged 2 to 4.9 years) had a greater pooled mean (dmft = 4.44; 95% CI: 3.84–5.03) than did those aged 5 to 6.9 years (dmft = 3.43; 95% CI: 3.20–3.65) or 7 to 10.5 years (dmft = 3.58; 95% CI: 3.28–3.88) (Fig. 2 and Figure S2). Meta-regression indicated that each one-year increase in mean age corresponded to a 0.18-point decrease in dmft (*p*-value = 0.027) (Table 2; Fig. 3-a).

**Table 2:** Meta-regression of dmft and DMFT indices according to mean age, percentage of female individuals, HDI, year of data collection, and global region

**Variables**	dmft (deciduous dentition)		DMFT (permanent dentition)	
	β-coefficient	*p*-value	β-coefficient	*p*-value
Univariate models				
Mean age (continuous)	-0.182	0.027	0.452	< 0.001
% female (continuous)	0.112	0.027	0.058	0.073
HDI (continuous)	-0.486	0.746	-2.462	0.171
North America (vs. Latin America)	1.218	0.005	1.739	0.035
Asia and Oceania (vs. Latin America)	-0.690	0.038	-1.183	0.006
Year of data collection (continuous)	-0.009	0.538	0.006	0.721
Multivariate model				
Mean age (continuous)	-0.075	0.405	0.399	< 0.001
% female (continuous)	0.100	0.030	0.035	0.149
HDI (continuous)	-1.265	0.509	-3.133	0.034
North America (vs. Latin America)	1.236	0.048	2.390	0.002
Asia and Oceania (vs. Latin America)	-0.365	0.496	− 0.017	0.966
Year of data collection (continuous)	-0.016	0.294	0.002	0.835

DMFT: decayed, missing due to caries, and filled teeth index for deciduous (dmft) and permanent (DMFT) dentition on the basis of the WHO criteria; *HDI* human development index

When the association between dmft and sample composition by sex was analyzed, studies with fewer than 49% young female individuals had a lower dmft (dmft = 3.44; 95% CI: 3.03–3.84) than those with between 49% and 51% (dmft = 3.70; 95% CI: 3.41-4.00) and more than 51% (dmft = 4.07; 95% CI: 3.63–4.52) (Fig. 2 and Figure S3). In the meta-regression, each 1% increase in the percentage of young female individuals corresponded to a 0.11-point increase in dmft (*p*-value = 0.027) (Table 2; Fig. 3-b).

When comparing dmft by country, studies from Canada had the highest mean dmft (dmft = 5.60; 95% CI: 4.77–6.43), followed by those from the United States (dmft = 4.52; 95% CI: 3.77–5.27) and Brazil (dmft = 3.83; 95% CI: 3.16–4.49). On the other hand, the lowest values were observed in Australia (dmft = 3.24; 95% CI: 3.03–3.44), India (dmft = 3.45; 95% CI: 2.14–4.77), and China (dmft = 3.50; 95% CI: 3.06–3.94) (Fig. 2 and Figure S4).

When countries were grouped by global region, the highest dmft was observed in North America (dmft = 5.23; 95% CI: 4.60–5.86), and the lowest dmft was observed in Asia and Oceania (dmft = 3.30; 95% CI: 3.11–3.50) (Fig. 2 and Figure S5). Meta-regression analyses corroborated these differences: compared with Latin America, the dmft was 1.22 points greater (*p*-value = 0.005) in North American studies and 0.69 points lower in Asia and Oceania (*p*-value = 0.038) (Table 2).

Analysis of the dmft according to the HDI indicated that the average dmft is greater in countries with the lowest HDI (dmft = 3.91; 95% CI: 3.35–4.45) and intermediate HDI (dmft = 4.02; 95% CI: 3.71–4.34) than in countries with the highest HDI (dmft = 3.39; 95% CI: 3.12–3.65) (Fig. 2 and Figure S6), although this difference was not significant in the meta-regression (*p*-value = 0.746) (Table 2).

Changes in dmft according to the data collection period revealed a lower dmft in the earlier period (1966–1999) (dmft = 3.53; 95% CI: 3.29–3.76), which increased between 2000 and 2010 (dmft = 4.04; 95% CI: 3.74–4.43), with a small decrease between 2011 and 2024 (dmft = 3.72; 95% CI: 3.22–4.21) (Fig. 2 and Figure S7). No significant trend was observed for dmft values over time (*p*-value = 0.538) (Table 2).

In the multivariate meta-regression model, the percentage of young female individuals (β-coefficient = 0.100; *p*-value = 0.030) and studies from North America rather than Latin America (β-coefficient = 0.123; *p*-value = 0.048) were associated with higher dmft regardless of age, HDI, and year of data collection (Table 2).

In the sensitivity analyses for deciduous dentition, all recalculated dmft estimates obtained after removing individual studies remained within the 95% CI of the original pooled analysis (Table S5 and Figure S8).

### Permanent dentition (DMFT)

The meta-analysis of permanent dentition caries included 39 studies reporting DMFT indices, which were analyzed by age group to account for the developmental progression of permanent teeth [35, 36, 38–44, 46–49, 51, 53, 56–59, 63, 75, 79, 90–94, 98, 102, 106, 112, 113, 117, 118, 136–140]. The overall pooled estimate revealed a global mean DMFT of 2.36 (95% CI: 2.18–2.55) among Indigenous children and adolescents, indicating a moderate but significant dental caries burden in permanent dentition across populations worldwide. Considerable heterogeneity was observed between the included studies (I²=99.6%) (Figure S10).

**Fig. 4:**
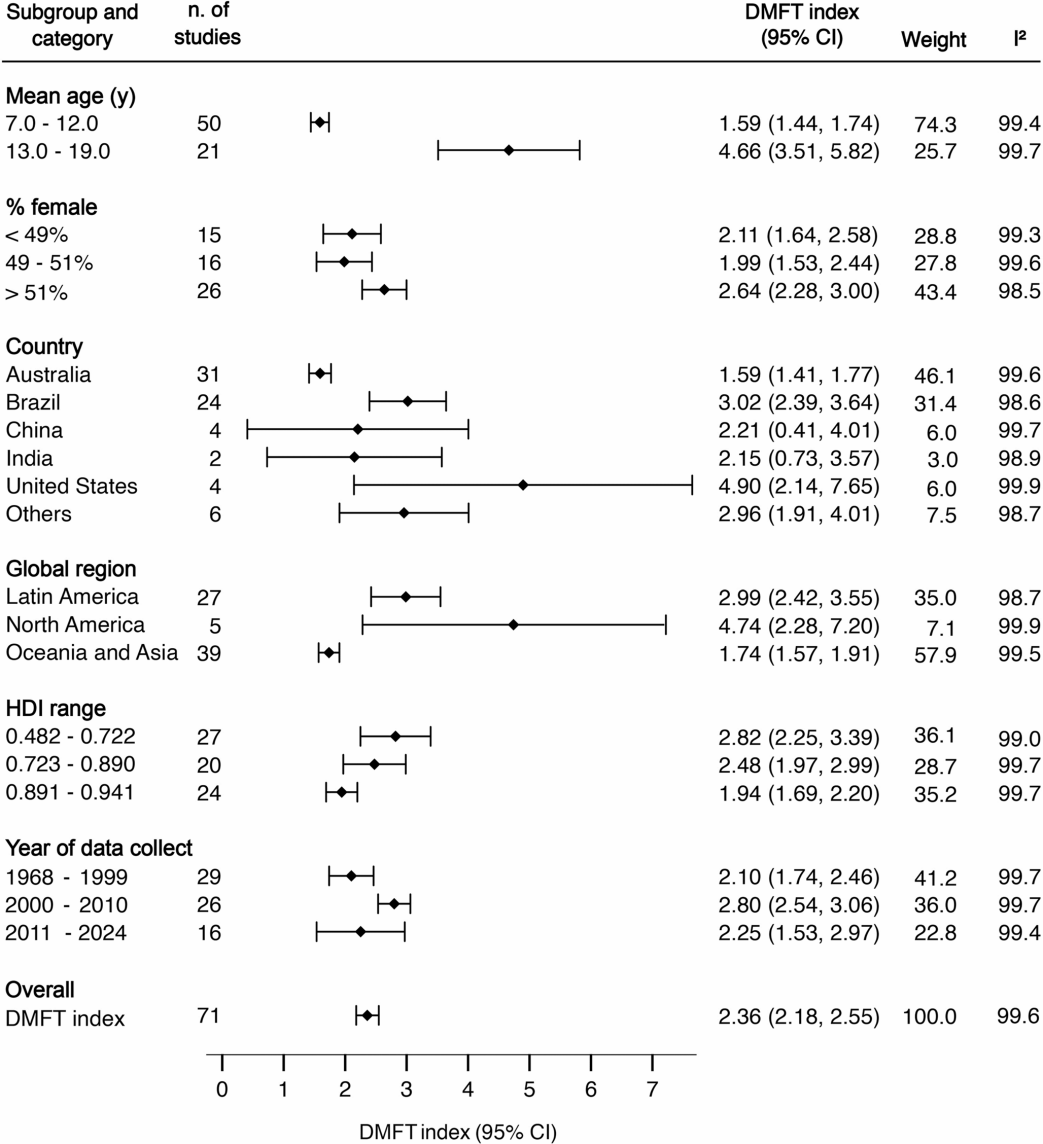
Graphical representation of the DMFT index of Indigenous children and adolescents by subgroup of mean age, percentage of female individuals, country, global region, HDI range, data collection period and overall data (DMFT: decayed, missing due to caries, and filled teeth index for permanent dentition; HDI: human development index)

The DMFT index was lower in children aged 7 to 12 years (DMFT = 1.59; 95% CI: 1.44–1.74) than in those aged 13 to 19 years (DMFT = 4.66; 95% CI: 3.51–5.82) (Fig. 4 and Figure S11). A statistically significant upward trend was observed in the meta-regression, with each additional year of age of participants corresponding to an increase of 0.45 points in DMFT values (*p*-value < 0.001) (Table 2; Fig. 3-c).

In the univariate regression models, no statistically significant differences were found in DMFT according to the percentage of young female individuals (*p*-value = 0.073) (Table 2; Fig. 3-d, and Figure S12), HDI (*p*-value = 0.171), or data collection period (*p*-value = 0.721) (Table 2).

With respect to variations in DMFT by country, the United States had the highest average (DMFT = 4.90; 95% CI: 2.14–7.65), followed by Brazil (DMFT = 3.02; 95% CI: 2.39–3.64), whereas Australia had the lowest (DMFT = 1.59; 95% CI: 1.41–1.77) (Fig. 4 and Figure S13).

When global regions were considered, the mean DMFT was highest in North America (DMFT = 4.74; 95% CI: 2.28–7.20), followed by Latin America (DMFT = 2.99; 95% CI: 2.42–3.55), and Asia and Oceania (DMFT = 1.74; 95% CI: 1.57–1.91) (Fig. 4 and Figure S14). In the meta-regression, North America was associated with greater DMFT (β-coefficient = 0.174; *p*-value = 0.035), whereas Asia and Oceania were associated with lower DMFT (β-coefficient=-1.183; *p*-value = 0.006) compared to Latin America. The effect observed in Asia and Oceania did not remain significant (*p*-value = 0.97) in the multivariate model, which included age, percentage of young female individuals, HDI, and the data collection period (Table 2).

With respect to the relationship between DMFT and HDI, countries with the lowest HDI had a greater mean DMFT (DMFT = 2.82; 95% CI: 2.25–3.39), followed by those with an intermediate HDI (DMFT = 2.48; 95% CI: 1.97–2.99) and then those with the highest HDI (DMFT = 1.94; 95% CI: 1.69–2.20) (Fig. 4 and Figure S15). Although HDI was not associated with DMFT in the univariate model, a higher HDI was significantly associated with lower DMFT values in the multivariate model (Table 2).

No significant trend was observed for dmft values over time (*p-*value = 0.538) (Table 2; Fig. 4 and Figure S16).

In the multivariate meta-regression model, in addition to the inverse association between DMFT and HDI, the mean age (β-coefficient = 0.100; *p*-value = 0.030) and studies from North America *versus* Latin America (β-coefficient = 0.123; *p*-value = 0.048) remained associated with greater DMFT regardless of age, HDI, and year of data collection (Table 2).

The results of sensitivity analyses for permanent dentition demonstrated stability, with all recalculated DMFT estimates remaining within the 95% CI of the main analysis regardless of which individual study was excluded (Table S6 and Figure S17).

### Publication bias

Statistical and visual assessments revealed evidence of publication bias affecting both deciduous and permanent dentition analyses. Egger regression analysis revealed small-study effects for both the dmft (*p*-value < 0.001) (Table S7) and DMFT analyses (*p*-value = 0.003) (Table S8). Consistently, funnel plots showed an asymmetry compatible with publication bias for both dmft (Figure S9) and DMFT (Figure S18).

## Discussion

### Main findings

This systematic review and meta-analysis provides a comprehensive global assessment of the experience of dental caries among Indigenous children and adolescents, incorporating data from 95 studies from 19 countries. Significant disparities in caries experience were observed between and within countries, with notable variations between the dmft and DMFT indices. Some demographic, geographic and socioeconomic factors were found to partially explain the heterogeneity between studies.

### Dental caries experience in deciduous dentition (dmft)

The global mean dmft of 3.75 indicates a substantial caries burden among Indigenous children worldwide, with marked discrepancies between studies from different countries. While studies from North America reported dmft indices 39% above the global average, those from Asia and Oceania reported indices 12% below the global average. These apparent geographical disparities may, at least in part, be due to differences in participants’ age distributions. While North American studies predominantly included children mostly under 5 years of age, Asian studies typically included older children with a mean age of approximately 8 years. Considering that physiological exfoliation of deciduous teeth occurs at older ages [141], the naturally lower dmft values in older children are biologically expected.

As observed for non-Indigenous populations, variations in oral health outcomes often reflect a combination of biological, social, and behavioral influences [25]. In this context, we observed that for each 1% increase in female participants, the dmft values increased by 0.10–0.11 points. This finding aligns with emerging evidence suggesting that biological factors such as differences in salivary composition and flow [142–144], combined with social [143] and behavioral factors [144], including culturally constructed eating patterns and common roles of female individuals [16], may contribute to increased caries susceptibility among the female population.

Substantial variation within countries highlights the diverse realities of Indigenous communities. In the United States, dmft indices ranged from 1.84 to 6.52 across regions [23], and in Brazil, they varied from 2.00 among Guarani populations [53] to approximately 6.00 among Xingu populations [36]. These differences illustrate how sociocultural, ethnic, economic, and environmental conditions, shaped by each Indigenous group’s distinct historical context of colonization [145] contribute to unequal oral health outcomes [21, 56].

The dmft did not vary according to the country’s level of development. While the HDI serves as a useful indicator for understanding oral health inequalities among different population groups [146], it may not fully capture socioeconomic inequalities or the specific vulnerabilities of Indigenous populations. Therefore, future studies would benefit from incorporating socioeconomic indicators beyond the HDI, such as income distribution, educational attainment, and geographic accessibility to health care, particularly dental care.

Additionally, the lack of change in dmft over nearly six decades suggests that dental caries prevention strategies have had limited effectiveness on Indigenous pediatric groups over time. The increasing consumption of sugar-rich foods by Indigenous populations [21, 147] further compounds this issue, as it is a significant risk factor for dental caries [148].

### Dental caries experience in permanent dentition (DMFT)

Likewise, the global mean DMFT of 2.36 for permanent dentition showed marked disparities. The age-related progression in permanent dentition, with the DMFT increasing 0.45 points per year, reflects the cumulative nature of caries experience. DMFT values increased nearly threefold from 1.6 among children (7–12 years) to 4.7 among adolescents (13–19 years), reinforcing the importance of age-oriented preventive strategies throughout development [15, 98].

Indigenous populations in North America presented higher DMFT values than those in Latin America, whereas populations in Asia/Oceania presented lower values. These differences likely reflect unequal access to public oral health measures, such as fluoride exposure [134], and variations in the availability and quality of dental services across healthcare systems [40, 111, 134].

The substantially increased consumption of ultra-processed foods, sugar-sweetened beverages, and other industrialized products, largely driven by the colonization process, food insecurity, and the disruption of traditional food systems, in North America may also help explain these findings [149]. In this sense, compared with white adults in Canada, Indigenous adults consume more ultra-processed foods and exhibit lower overall diet quality, which may further contribute to their greater caries experience [150].

The influence of commercial determinants, which have a strong impact on social conditions and consumption patterns among populations [145, 151, 152], has gradually increased across nearly all regions of the world, promoting significant changes in food systems [153]. This trend has been associated with general and oral health conditions [154], because ultra-processed foods are high in sugar, and their consumption is associated with poor dietary quality [153]. In Indigenous contexts, commercial determinants manifest themselves through the expansion marketing of ultra-processed foods [145, 155], which are nutrient-poor and more readily available than fresh and traditional foods, a dynamic that has caused a disconnection from traditional Indigenous food systems and, consequently, increased vulnerability to dental caries [149].

A significant inverse relationship was observed between the HDI and DMFT values, with higher socioeconomic development associated with lower caries experience. This pattern is consistent with the variables included in the multivariate model: countries with very high HDI, such as Australia and New Zealand, tend to provide broader fluoridation coverage and better access to dental services than countries with lower HDI [134]. However, even in highly developed settings, some Indigenous groups may present elevated DMFT levels [57], which could be associated with structural factors, such as territorial displacement [24], and barriers to accessing care.

Similarly, Indigenous groups in lower HDI countries, represented in this review by studies from Latin America, may present lower DMFT values because of differing exposure patterns, such as the reduced availability of industrialized food products, the partial preservation of traditional dietary practices, or the presence of structured oral health programs [156]. Together, these results suggest that the relationship between the HDI and the DMFT index among Indigenous populations is shaped not only by the level of national development but also by internal socioeconomic disparities, historical determinants, and interactions among dietary transitions, healthcare access, and regional health policies.

In the general population, this relationship has been documented in economically disadvantaged European countries, where children in Eastern Europe have worse oral health than those in high-income northern European countries [157]. Similar associations have been observed across diverse countries, including Australia, New Zealand, and Brazil [158].

### Time trends

No statistically significant differences in caries experience were found across the study periods (1968–2024), which is consistent with previous systematic reviews showing stability in caries experience among Indigenous South American populations between 1964 and 2018 [30]. However, this apparent stability masks considerable heterogeneity among Indigenous groups, with some experiencing increases while others showing reductions or stabilization in disease prevalence.

The geographic distribution of studies reflects both the global scope of Indigenous dental health research and regional research concentrations in countries with substantial Indigenous populations and established oral health surveillance systems. Australia contributed the largest number of publications (32 studies), characterized by more robust methodological approaches and representative samples encompassing diverse geographic contexts and socioeconomic factors, including national survey data [35, 38, 67, 93, 100, 101]. In contrast, Brazilian studies, despite increasing in recent decades, typically involve smaller samples with limited ethnic representativeness, given the country’s substantial Indigenous diversity [40, 46, 53, 56, 78].

The predominance of cross-sectional designs (97% of studies) is consistent with the observational nature of dental caries surveillance research and reflects practical challenges of conducting longitudinal studies in Indigenous communities, including geographic remoteness, cultural considerations, and resource limitations [159].

### Study limitations and methodological considerations

Several limitations warrant careful interpretation of the results. Methodological heterogeneity was substantial, with many studies not reporting the use of WHO diagnostic criteria [23, 37, 38, 42, 47, 50, 55, 57, 59, 75, 81, 91, 93–95, 98, 105, 113, 114, 127] or inter/intra-examiner calibration [23, 35, 38, 43, 57, 58, 76, 81, 87, 93, 94, 112, 118, 120]. However, most studies demonstrated high methodological quality, with rigorous statistical analyses [34]. Thus, despite minor limitations in the characterization of study subjects and settings, and in the description of objective measurement criteria and procedures the findings are robust and provide strong evidence on the topic.

Sample selection biases were evident, with some studies exclusively recruiting from school dental clinics [35, 38, 93], potentially overrepresenting children with dental problems or, conversely, underrepresenting those whose caregivers showed greater oral health interest [113]. Publication bias was detected through Egger regression tests, suggesting the preferential publication of studies reporting higher caries prevalence or more dramatic health disparities. This bias likely reflects editorial and researcher tendencies to publish studies demonstrating significant health inequities, potentially leading to overestimation of the true caries burden among Indigenous populations. These findings highlight the need for comprehensive publication of all relevant research regardless of effect size magnitude.

The absence of studies from many Indigenous groups and geographic regions limits the scope and representativeness of these studies. Additionally, this analysis did not examine important determinants such as fluoride exposure, healthcare access, oral hygiene behaviors, dietary patterns, and socioenvironmental factors that deserve investigation to deepen the understanding of Indigenous oral health disparities.

### Public health implications

Despite these limitations, our findings highlight the urgent need for culturally adapted public policies and oral health programs addressing Indigenous populations’ specific needs. Given that deciduous dentition caries serves as a strong risk indicator for permanent dentition caries [160, 161], the epidemiological patterns observed over recent decades will likely persist if effective interventions are not implemented. This persistence suggests inadequate access to preventive measures, dental care, and effective public policies targeting Indigenous oral health.

Upcoming interventions should include culturally appropriate health promotion and prevention strategies, community water fluoridation where feasible, expanded access to dental services, and comprehensive programs addressing social determinants of health. Cultural adaptation is essential for ensuring community engagement, reducing inequalities, and improving oral health outcomes among Indigenous populations [15, 30, 93, 162].

Importantly, the present findings are not exclusive to Indigenous populations and should not reinforce the stigmatization of Indigenous health in relation to non-Indigenous populations [163]. In addition, Indigenous data sovereignty is still an issue under development, linked to self-determination rather than merely to data protection [8]. This principle is aligned with the UNDRIP, which emphasizes collective rights and contextualizes Indigenous experiences within their broader historical, political, and cultural contexts [3]. It should also incorporate the CARE Principles [9]. This debate is essential for situating Indigenous health within a contemporary global public health perspective, extending well beyond dental health.

### Research priorities

Future research should prioritize large-scale epidemiological studies with a national scope and that incorporate diverse Indigenous populations, standardized methodologies, and comparable indicators. Such investigations are essential for identifying disease distribution patterns, mapping risk factors, and supporting the development of equitable, culturally appropriate public policies and prevention strategies. Longitudinal studies would enhance the understanding of caries progression over time and the impacts of changing social and environmental determinants on Indigenous oral health.

## Conclusion

Dental caries among Indigenous children and adolescents is a public health problem in different global contexts. Despite considerable heterogeneity in caries indices among the analyzed studies, the results revealed geographical variation in caries experience across both dentition types and age groups. Additionally, a significant association was identified between the percentage of female individuals in the sample and the experience of caries in deciduous dentition, as well as between the HDI and caries in permanent dentition. These findings underscore the importance of intersectoral actions and culturally sensitive, socially appropriate public policies for oral health that address the specific needs of each ethnic group. Prioritizing oral health promotion and prevention strategies is essential for reducing the prevalence of dental caries and oral health inequalities among Indigenous children and adolescents worldwide.

## Supplementary Information

Below is the link to the electronic supplementary material.

Supplementary Material 1 (PDF 6.44 MB)

## Data Availability

No datasets were generated or analysed during the current study.

## References

[1] Sarfati D, Robson B, Garvey G et al (2018) Improving the health of Indigenous people globally. Lancet Oncol 19:e276 29893251 10.1016/S1470-2045(18)30336-X

[2] Stephens C, Porter J, Nettleton C, Willis R (2006) Disappearing, displaced, and undervalued: a call to action for Indigenous health worldwide. lancet 367:2019–2028 16782493 10.1016/S0140-6736(06)68892-2

[3] United Nations (2007) United Nations declaration on the rights of indigenous peoples. United Nations 12:1–18

[4] Armitage A (1995) Comparing the policy of aboriginal assimilation: Australia, Canada, and New Zealand. UBC

[5] Reid P, Cormack D, Paine S-J (2019) Colonial histories, racism and health—The experience of Māori and Indigenous peoples. Public Health 172:119–124 31171363 10.1016/j.puhe.2019.03.027

[6] Redvers N, Celidwen Y, Schultz C et al (2022) The determinants of planetary health: an Indigenous consensus perspective. Lancet Planet Health 6:e156–e163 35150624 10.1016/S2542-5196(21)00354-5

[7] Coimbra CEA, Flowers NM, Salzano FM, Santos RV (2004) The Xavánte in transition: health, ecology, and bioanthropology in Central Brazil. University of Michigan Press

[8] Hudson M, Carroll SR, Anderson J et al (2023) Indigenous Peoples’ rights in data: a contribution toward Indigenous Research Sovereignty. Front Res Metr Anal 8:1173805 37215248 10.3389/frma.2023.1173805 PMC10192690

[9] Carroll SR, Garba I, Figueroa-Rodríguez OL et al (2020) The CARE principles for indigenous data governance. Data Sci J. 10.5334/dsj-2020-043. 43. https://doi.org

[10] Marrone S (2007) Understanding barriers to health care: a review of disparities in health care services among indigenous populations. Int J Circumpolar Health 66:188–198 17655060 10.3402/ijch.v66i3.18254

[11] Bramley D, Hebert P, Tuzzio L, Chassin M (2005) Disparities in indigenous health: a cross-country comparison between New Zealand and the United States. Am J Public Health 95:844–850 15855464 10.2105/AJPH.2004.040907 PMC1449267

[12] Anderson I, Crengle S, Kamaka ML et al (2006) Indigenous health in Australia, New Zealand, and the Pacific. Lancet 367:1775–1785 16731273 10.1016/S0140-6736(06)68773-4

[13] Brasil Fundação Nacional de Saúde (2002) Política Nacional de Atenção à Saúde dos Povos Indígenas. Brasília-DF

[14] King M, Smith A, Gracey M (2009) Indigenous health part 2: the underlying causes of the health gap. Lancet 374:76–85 19577696 10.1016/S0140-6736(09)60827-8

[15] Nath S, Poirier BF, Ju X et al (2021) Dental health inequalities among indigenous populations: a systematic review and meta-analysis. Caries Res 55:268–287 34107490 10.1159/000516137 PMC8491513

[16] World Health Organization (2024) Global strategy and action plan on oral health 2023–2030. World Health Organization, Geneva

[17] Mialon M (2020) An overview of the commercial determinants of health. Global Health 16:74 32807183 10.1186/s12992-020-00607-x PMC7433173

[18] Gilmore AB, Fabbri A, Baum F et al (2023) Defining and conceptualising the commercial determinants of health. Lancet 401:1194–1213 36966782 10.1016/S0140-6736(23)00013-2

[19] Friel S, Collin J, Daube M et al (2023) Commercial determinants of health: future directions. Lancet 401:1229–1240 36966784 10.1016/S0140-6736(23)00011-9

[20] World Health Organization (2022) Global oral health status report: towards universal health coverage for oral health by 2030. World Health Organization, Geneva

[21] Arantes R (2005) Saúde bucal dos povos indígenas no Brasil: panorama atual e perspectivas. In: Coimbra Jr. CEA, Santos RV, Escobar AL, editors. Epidemiologia e saúde dos povos indígenas no Brasil. Fiocruz, Rio de Janeiro

[22] Poirier B, Soares G, Sethi S et al (2023) Facilitators and challenges to maintaining oral Health for Indigenous communities globally: a qualitative systematic review. J Health Care Poor Underserved 34:377–398 37464501 10.1353/hpu.2023.0025

[23] Phipps KR, Ricks TL, Manz MC, Blahut P (2012) Prevalence and severity of dental caries among American Indian and Alaska Native preschool children. J Public Health Dent 72:208–215 22515656 10.1111/j.1752-7325.2012.00331.x

[24] Christian B, Blinkhorn A (2012) A review of dental caries in Australian Aboriginal children: the health inequalities perspective. Rural Remote Health 12(4):2032 23098560

[25] Alves Filho P, Santos RV, Vettore MV (2014) Fatores associados a cárie dental e doença periodontal em indígenas na América Latina: revisão sistemática. Revista Panam de Salud Pública 35:67–77 24626450

[26] Parker EJ, Jamieson LM, Broughton J et al (2010) The oral health of Indigenous children: a review of four nations. J Paediatr Child Health 46:483–486 20854317 10.1111/j.1440-1754.2010.01847.x

[27] World Health Organization (2013) Oral health surveys: basic methods. World Health Organization, Geneva

[28] Wang X, Ghanbarzadegan A, Sohn W et al (2024) Inequalities in dental caries among Indigenous and non-Indigenous children in Australia: A literature review. Aust Dent J 69:73–81 38197608 10.1111/adj.13005

[29] Schuch HS, Haag DG, Kapellas K et al (2017) The magnitude of Indigenous and non-Indigenous oral health inequalities in Brazil, New Zealand and Australia. Community Dent Oral Epidemiol 45:434–441 28509420 10.1111/cdoe.12307

[30] Soares GH, Pereira NF, Biazevic MGH et al (2019) Dental caries in South American Indigenous peoples: A systematic review. Community Dent Oral Epidemiol 47:142–152 30506750 10.1111/cdoe.12436

[31] Rebelo Vieira JM, Pereira JV, Sponchiado EC Jr. et al (2023) Prevalence of dental caries, periodontal disease, malocclusion, and tooth wear in indigenous populations in Brazil: a systematic review and meta-analysis. Braz Oral Res 37:e094 37820252 10.1590/1807-3107bor-2023.vol37.0094

[32] Moher D, Liberati A, Tetzlaff J et al (2010) Preferred reporting items for systematic reviews and meta-analyses: the PRISMA statement. Int J Surg 8:336–341 20171303 10.1016/j.ijsu.2010.02.007

[33] Stroup DF, Berlin JA, Morton SC et al (2000) Meta-analysis of observational studies in epidemiology: a proposal for reporting. JAMA 283:2008–2012 10789670 10.1001/jama.283.15.2008

[34] Aromataris E, Lockwood C, Porritt K et al (2024) JBI Manual for Evidence Synthesis. JBI

[35] Ha DH (2014) Oral health of Australian Indigenous children compared to non-Indigenous children enrolled in school dental services. Aust Dent J 59:395–400. 10.1111/adj.12205 25040876 10.1111/adj.12205

[36] Lemos PN, Rodrigues DA, Frazão P et al (2018) Cárie dentária em povos do Parque Indígena do Xingu, Brasil, 2007 e 2013. Epidemiol Serv Saude 27:e20171725. 10.5123/S1679-49742018000100005 29412349 10.5123/S1679-49742018000100005

[37] Guisilini AC, Bulgareli JV, Guerra LM et al (2021) Caries experience in children under 5 years old in the Xingu indigenous park in Brazil. Braz J Oral Sci 20:1–11. 10.20396/bjos.v20i00.8661606

[38] Ha DH, Do LG, Luzzi L et al (2016) Changes in area-level socioeconomic status and oral health of indigenous Australian children. J Health Care Poor Underserved 27:110–124. 10.1353/hpu.2016.0034 27763435 10.1353/hpu.2016.0034

[39] Baldisserotto J, Ferreira AM, Warmling CM (2019) Condições de saúde bucal da população indígena guarani moradora no Sul do Brasil. Cad Saude Colet 27:468–475. 10.1590/1414-462x201900040354

[40] Arantes R, Jamieson LM, Frazão P (2021) Dental caries, periodontal disease and restorative dental care among Indigenous and non-Indigenous groups in Brazil: A descriptive study. Community Dent Oral Epidemiol 49:63–69. 10.1111/cdoe.12577 32985016 10.1111/cdoe.12577

[41] Mauricio HDA, Moreira RDS (2014) Oral health status of the ethnic group Xukuru from Ororubá: Multilevel analysis. Revista Brasileira de Epidemiologia 17:787–800. 10.1590/1809-4503201400030017 25272269 10.1590/1809-4503201400030017

[42] Jamieson LM, Do LG, Bailie RS et al (2013) Associations between area-level disadvantage and DMFT among a birth cohort of Indigenous Australians. Aust Dent J 58:75–81. 10.1111/adj.12017 23441795 10.1111/adj.12017

[43] Kailis DG (1979) Australian Aboriginal studies: I. The effect of socio-economic changes on the prevalence of caries in 6–14 year‐old children resident at Bathurst Island and Groote Eylandt in the Northern Territory of Australia, 1971. Aust Dent J 24:363–368. 10.1111/j.1834-7819.1979.tb05810.x 294243 10.1111/j.1834-7819.1979.tb05810.x

[44] Miranda KC, de O, Souza TAC, Leal SC (2018) Caries prevalence among Brazilian indigenous population of urban areas based on the 2010 national oral health survey. Ciencia e Saude Coletiva 23:1313–1322. 10.1590/1413-81232018234.18082016 29694590 10.1590/1413-81232018234.18082016

[45] Arantes R, Santos RV, Frazão P, Coimbra CEA (2009) Caries, gender and socio-economic change in the Xavante Indians from Central Brazil. Ann Hum Biol 36:162–175. 10.1080/03014460802672844 19184758 10.1080/03014460802672844

[46] Sampaio FC, Freitas CHS, de Cabral M, de Machado MB AB (2010) Dental caries and treatment needs among indigenous people of the Potiguara Indian reservation in Brazil. Revista Panam de Salud Pública 27:246–251 10.1590/s1020-49892010000400002 20512226

[47] Hopcraft M, Chow W (2007) Dental caries experience in Aboriginal and Torres Strait Islanders in the Northern Peninsula Area, Queensland. Aust Dent J 52:300–304. 10.1111/j.1834-7819.2007.tb00506.x 18265686 10.1111/j.1834-7819.2007.tb00506.x

[48] Rigonatto DDL, Antunes JLF, Frazão P (2001) Dental caries experience in Indians of the Upper Xingu, Brazil. Rev Inst Med Trop Sao Paulo 43:93–98 11340483 10.1590/s0036-46652001000200008

[49] Arantes R, Santos RV, Coimbra CE Jr. (2001) Oral health among the Xavánte Indians in Pimentel Barbosa, Mato Grosso, Brazil. Cadernos de saúde pública / Ministério da Saúde, Fundação Oswaldo Cruz. Escola Nac de Saúde Pública 17:375–384. 10.1590/s0102-311x2001000200012 10.1590/s0102-311x2001000200012 11283768

[50] Lawrence HP, Binguis D, Douglas J et al (2009) Oral health inequalities between young Aboriginal and non-Aboriginal children living in Ontario, Canada. Community Dent Oral Epidemiol 37:495–508. 10.1111/j.1600-0528.2009.00497.x 19780768 10.1111/j.1600-0528.2009.00497.x

[51] Barrett MJ, Williamson JJ (1972) Oral health of Australian aborigines: survey methods and prevalence of dental caries. Aust Dent J 17:37–50. 10.1111/j.1834-7819.1972.tb02744.x 4402739 10.1111/j.1834-7819.1972.tb02744.x

[52] de Oliveira MR, Marinho AMCL, Bendo CB et al (2024) Profile of Dental Caries in Eastern and Western Parakanã Children at Amazônia Paraense, Brazil. Pesqui Bras Odontopediatria Clin Integr 24. 10.1590/pboci.2024.043

[53] Alves Filho P, Santos RV, Vettore MV (2009) Saúde bucal dos índios Guaraní no Estado do Rio de Janeiro Brasil. Cad Saude Publica 25:37–46 19180285 10.1590/s0102-311x2009000100004

[54] Kruger E, Smith K, Atkinson D, Tennant M (2008) The oral health status and treatment needs of Indigenous adults in the Kimberley region of Western Australia. Aust J Rural Health 16:283–289. 10.1111/j.1440-1584.2008.00985.x 18808486 10.1111/j.1440-1584.2008.00985.x

[55] Lee J, Schroth RJ, Sturym M et al (2022) Oral Health Status and Oral Health–Related Quality of Life of First Nations and Metis Children. JDR Clin Trans Res 7:435–445. 10.1177/23800844211037992 34672839 10.1177/23800844211037992 PMC9490442

[56] Arantes R, Frazão P (2016) Income as a protective factor for dental caries among indigenous people from central Brazil. J Health Care Poor Underserved 27:81–89. 10.1353/hpu.2016.0043 27763432 10.1353/hpu.2016.0043

[57] Niendorff WJ, Jones CM (2000) Prevalence and severity of dental caries among American Indians and Alaska Natives. J Public Health Dent 60:243–249 11243042 10.1111/j.1752-7325.2000.tb04069.x

[58] Miyazaki H, Takehara T (1988) Prevalence of dental caries in Taiwan Aboriginal children — Bunun, Paiwan, Rukai, Ami, and Yami Tribes. Aust Dent J 33:226–230. 10.1111/j.1834-7819.1988.tb01319.x 3263852 10.1111/j.1834-7819.1988.tb01319.x

[59] Jamieson LM, Sayers SM, Roberts-Thomson KF (2013) Associations between oral health and height in an Indigenous Australian birth cohort. Community Dent Health 30:58–64 23550509

[60] Hirooka LB, Mestriner- W, Mestriner SF et al (2014) Dental caries in mother-child pairs from Xingu. Braz J Oral Sci 13:43–46

[61] Schroth RJ, Moore P, Brothwell DJ (2005) Prevalence of early childhood caries in 4 Manitoba communities. J Can Dent Assoc (Tor) 71:567 16202195

[62] Kroon J, Lalloo R, Tadakamadla SK, Johnson NW (2019) Dental caries experience in children of a remote Australian Indigenous community following passive and active preventive interventions. Community Dent Oral Epidemiol 47:470–476. 10.1111/cdoe.12486 31328295 10.1111/cdoe.12486 PMC6899803

[63] Jamieson LM, Armfield JM, Roberts-Thomson KF (2007) Dental caries trends among indigenous and non-indigenous Australian children. Community Dent Health 24:238–246 18246842

[64] Australian Buerau of Statistics (2016) Estimates of Aboriginal and Torres Strait Islander Australians. https://www.abs.gov.au/statistics/people/aboriginal-and-torres-strait-islander-peoples/estimates-aboriginal-and-torres-strait-islander-australians/jun-2016#data-downloads. Accessed 11 Sep 2024

[65] So M, Ellenikiotis YA, Husby HM et al (2017) Early childhood dental caries, mouth pain, and malnutrition in the ecuadorian amazon region. Int J Environ Res Public Health 14. 10.3390/ijerph14050550 10.3390/ijerph14050550 PMC5452000 28531148

[66] Caires NCM, de Brito LCN, Vieira LQ, Sobrinho APR (2018) Epidemiological analysis and need for endodontic treatment among the indigenous Sateré-Mawé and Tikuna. Braz Oral Res 32:1–8. 10.1590/1807-3107bor-2018.vol32.0019 10.1590/1807-3107bor-2018.vol32.0019 29538481

[67] Lalloo R, Jamieson LM, Ha D, Luzzi L (2016) Inequalities in tooth decay in Australian children by neighbourhood characteristics and indigenous status. J Health Care Poor Underserved 27:161–177. 10.1353/hpu.2016.0045 27763439 10.1353/hpu.2016.0045

[68] Jayashantha P, Johnson NW (2016) Oral health status of the veddas— Sri Lankan indigenous people. J Health Care Poor Underserved 27:139–147. 10.1353/hpu.2016.0039 27763437 10.1353/hpu.2016.0039

[69] Martinez-Mier EA, Zandona AF (2013) The impact of gender on caries prevalence and risk assessment. Dent Clin 57:301–315 10.1016/j.cden.2013.01.001 23570807

[70] UNDP (2024) Human Development Report 2023/2024: Breaking the Gridlock-Reimagining Cooperation in a Polarized World. United Nations, New York

[71] Deeks J, Higgins J, Altiman D et al (2023) Chap. 10: analysing data and undertaking meta-analyses. In: J. Higgins & J. Thomas. Cochrane Handbook for Systematic Reviews of Interventions version 63

[72] Page MJ, Sterne JAC, Higgins JPT, Egger M (2021) Investigating and dealing with publication bias and other reporting biases in meta-analyses of health research: A review. Res Synth Methods 12:248–259 33166064 10.1002/jrsm.1468

[73] Abdul Kadir R, Adnan N (1989) Dental caries experience of 7 to 12-year old West Malaysian aborigines (Temuan tribe). Odontostomatol Trop 12:7–11 2631083

[74] Arrow P (2016) Oral health of schoolchildren in Western Australia. Aust Dent J 61:333–341 26296432 10.1111/adj.12368

[75] Batliner T, Wilson A, Davis E et al (2016) A comparative analysis of oral health on the Santo Domingo Pueblo Reservation. J Community Health 41:535–540 26611694 10.1007/s10900-015-0127-9 PMC4842215

[76] Berhan Nordin EA, Shoaib LA, Mohd Yusof ZY et al (2019) Oral health-related quality of life among 11–12 year old indigenous children in Malaysia. BMC Oral Health 19(1):152. 10.1186/s12903-019-0833-2 31307462 10.1186/s12903-019-0833-2 PMC6631802

[77] Butten K, Johnson NW, Hall KK et al (2019) Risk factors for oral health in young, urban, Aboriginal and Torres Strait Islander children. Aust Dent J 64:72–81. 10.1111/adj.12662 30375649 10.1111/adj.12662 PMC6392135

[78] Carneiro MCG, Santos RV, Garnelo L et al (2008) Cárie dentária e necessidade de tratamento odontológico entre os índios Baniwa do Alto Rio Negro, Amazonas. Cien Saude Colet 13:1985–1992 18833376 10.1590/s1413-81232008000600034

[79] Chang WK (1971) Five years observation on the dental caries experience of the Saisiat tribe aboriginal children in Taiwan. J Formos Med Assoc 70:208–212 4147761

[80] Chinnakotla B, Susarla SM, Mohan DC et al (2022) Associations between maternal education and child nutrition and oral health in an indigenous population in Ecuador. Int J Environ Res Public Health 20:473 36612796 10.3390/ijerph20010473 PMC9819843

[81] D’Mello G, Chia L, Hamilton SD et al (2011) Childhood obesity and dental caries among paediatric dental clinic attenders. Int J Paediatr Dent 21:217–222 21332849 10.1111/j.1365-263X.2011.01112.x

[82] Dasanayake AP, Caufield PW (2002) Prevalence of dental caries in Sri Lankan aboriginal Veddha children. Int Dent J 52:438–444. 10.1111/j.1875-595X.2002.tb00639.x 12553398 10.1111/j.1875-595x.2002.tb00639.x

[83] Davies MJ, Spencer AJ, Westwater A, Simmons B (1997) Dental caries among Australian Aboriginal, non-Aboriginal Australian-born, and overseas-born children. Bull World Health Organ 75:197 9277006 PMC2486947

[84] de Muñniz BR (1985) Epidemiologic oral health survey of Argentine children. Community Dent Oral Epidemiol 13:328–333 3866653 10.1111/j.1600-0528.1985.tb00466.x

[85] del Rio Gomez I (1991) Dental caries and mutans streptococci in selected groups of urban and native Indian schoolchildren in Mexico. Community Dent Oral Epidemiol 19:98–100 2049931 10.1111/j.1600-0528.1991.tb00119.x

[86] Dimitropoulos Y, Gunasekera H, Blinkhorn A et al (2018) A collaboration with local Aboriginal communities in rural New South Wales, Australia to determine the oral health needs of their children and develop a community-owned oral health promotion program. Rural Remote Health 18(2):4453. 10.22605/RRH4453 29890837 10.22605/RRH4453

[87] Dogar F, Kruger E, Dyson K et al (2011) Oral health of pre-school children in rural and remote Western Australia. Rural Remote Health 11:2011. 10.3316/informit.328350238138452 22166148

[88] Drummond AMA, Ferreira EF, Gomes VE, Marcenes W (2015) Inequality of experience of dental caries between different ethnic groups of Brazilians aged 15 to 19 years. PLoS ONE 10(12):e0145553. 10.1371/journal.pone.0145553 26694321 10.1371/journal.pone.0145553 PMC4692284

[89] Endean C, Roberts-Thomson K, Wooley S (2004) Anangu oral health: The status of the Indigenous population of the Anangu Pitjantjatjara lands. Aust J Rural Health 12:99–103. 10.1111/j.1440-1854.2004.00566.x 15200519 10.1111/j.1440-1854.2004.00566.x

[90] Gonçalves ÉM, Cavalcanti LC, Firmino RT et al (2015) Dental caries experience among indigenous children and adolescents. J Oral Sci 57:123–129. 10.2334/josnusd.57.123 26062861 10.2334/josnusd.57.123

[91] Gowda S, Thomson WM, Page LAF, Croucher NA (2009) Dental caries experience of children in Northland/Te Tai Tokerau. NZ Dent J 105:116–120 20000190

[92] Grim CW, Broderick EB, Jasper B, Phipps KR (1994) A comparison of dental caries experience in Native American and Caucasian children in Oklahoma. J Public Health Dent 54:220–227 7799296 10.1111/j.1752-7325.1994.tb01218.x

[93] Ha DH, Crocombe LA, Mejia C G (2014) Clinical oral health of Australia’s rural children in a sample attending school dental services. Aust J Rural Health 22:316–322. 10.1111/ajr.12107 25495626 10.1111/ajr.12107

[94] Hagens ERC, Preatoni SM, Bazzini EM et al (2023) Oral Health Status of Ngäbe-Buglé Children in Panama: A Cross Sectional Study. Children 10(2):294. 10.3390/children10020294 36832423 10.3390/children10020294 PMC9955745

[95] Hallett KB, O’Rourke PK (2002) Dental caries experience of preschool children from the north Brisbane region. Aust Dent J 47:331–338 12587770 10.1111/j.1834-7819.2002.tb00547.x

[96] Hirata JM, Bergamaschi O, de Oliveira Filho A et al (1977) Caries prevalence in Indian children from Xingu National Park. Rev Fac Odontol Sao Paulo 15:189–198 291086

[97] Homan BT, Davies GN (1973) An oral health survey of Aborigines and Torres Strait Islanders in far North Queensland. Aust Dent J 18:75–87. 10.1111/j.1834-7819.1973.tb04995.x 4146260 10.1111/j.1834-7819.1973.tb04995.x

[98] Jamieson LM, Armfield JM, Roberts-Thomson KF, Sayers SM (2010) A retrospective longitudinal study of caries development in an Australian Aboriginal Birth Cohort. Caries Res 44:415–420. 10.1159/000316665 20720421 10.1159/000316665

[99] Jamieson LM, Do L, Kapellas K et al (2021) Oral health changes among Indigenous and non-Indigenous Australians: findings from two national oral health surveys. Aust Dent J 66:S48–S55. 10.1111/adj.12849 33899961 10.1111/adj.12849

[100] Jamieson LM, Armfield JM, Roberts-Thomson KF (2006) The Role of Location in Indigenous and Non‐Indigenous Child Oral Health. J Public Health Dent 66:123–130. 10.1111/j.1752-7325.2006.tb02567.x 16711632 10.1111/j.1752-7325.2006.tb02567.x

[101] Jamieson LM, Armfield JM, Roberts-Thomson KF (2007) Indigenous and non-indigenous child oral health in three Australian states and territories. Ethn Health 12:89–107. 10.1080/13557850601002197 17132586 10.1080/13557850601002197

[102] Jamieson LM, Roberts-Thomson KF, Sayers SM (2010) Dental caries risk indicators among Australian Aboriginal young adults. Community Dent Oral Epidemiol 38:213–221. 10.1111/j.1600-0528.2009.00519.x 20059488 10.1111/j.1600-0528.2009.00519.x

[103] John JB, Asokan S, Aswanth KP et al (2015) Dental caries and the associated factors influencing it in tribal, suburban and urban school children of Tamil Nadu, India: a cross sectional study. J Public Health Res 4:jphr–2015 10.4081/jphr.2015.361 PMC4407035 25918690

[104] Johnson NW, Lalloo R, Kroon J et al (2014) Effectiveness of water fluoridation in caries reduction in a remote Indigenous community in Far North Queensland. Aust Dent J 59:366–371. 10.1111/adj.12190 24820049 10.1111/adj.12190

[105] Jones DB, Schlife CM, Phipps KR (1992) An oral health survey of Head Start children in Alaska: oral health status, treatment needs, and cost of treatment. J Public Health Dent 52:86–93 1564696 10.1111/j.1752-7325.1992.tb02249.x

[106] Kadir RA, Yassin AT (1990) Experience of dental caries among aboriginal children in Selangor Malaysia. J Nihon Univ Sch Dent. 10.2334/josnusd1959.32.275 2074496 10.2334/josnusd1959.32.275

[107] Koike BDV, Valões RMP, Cazal C et al (2024) Oral health of an indigenous population in northeastern Brazil: a cross-sectional Study of the Fulni-ô ethnic group. Sao Paulo Med J 142. 10.1590/1516-3180.2022.0355.R1.10042023 10.1590/1516-3180.2022.0355.R1.10042023 PMC10393376 37531491

[108] Kruger E, Dyson K, Tennant M (2005) Pre-school child oral health in rural Western Australia. Aust Dent J 50:258–262. 10.1111/j.1834-7819.2005.tb00370.x 17016892 10.1111/j.1834-7819.2005.tb00370.x

[109] Kumar KR, Reddy NV, Prasad Rao VA et al (2013) Prevalence of Caries in Urban, Rural & Tribal Children in Tamil Nadu. JIDA: Journal of Indian Dental Association 7

[110] Lalloo R, Myburgh NG, Hobdell MH (1999) Dental caries, socio-economic development and national oral health policies. Int Dent J 49:196–202 10858754 10.1111/j.1875-595x.1999.tb00522.x

[111] Mauricio H, de Fávaro A, da Moreira TR S (2024) Desigualdade em saúde bucal: caracterização do povo indígena Xukuru do Ororubá, Pernambuco, Brasil. Cien Saude Colet 29:e06712024 39775637 10.1590/1413-812320242912.06712024

[112] Medina W, Hurtig AK, San Sebastian M et al (2008) Dental Caries in 6-12-Year-Old Indigenous and Non-Indigenous Schoolchildren in the Amazon Basin of Ecuador. Braz Dent J 19:83–86 18438566 10.1590/s0103-64402008000100015

[113] Peressini S, Leake JL, Mayhall JT et al (2004) Prevalence of dental caries among 7- and 13-year-old First Nations children District of Manitoulin Ontario. Can Dent Assoc 70:382–383 15175117

[114] Peressini S, Leake JL, Mayhall JT et al (2004) Prevalence of early childhood caries among First Nations children, District of Manitoulin, Ontario. Int J Paediatr Dent 14:101–110 15005698 10.1111/j.1365-263x.2004.00532.x

[115] Poni NA, Ribas-Pérez D, Flores-Fraile J et al (2023) Descriptive Study of Oral Health in an Indigenous Child Population of Baka Pygmies in Cameroon. Dent J (Basel) 11. 10.3390/dj11100237 10.3390/dj11100237 PMC10605497 37886922

[116] Prasai Dixit L, Shakya A, Shrestha M, Shrestha A (2013) Dental caries prevalence, oral health knowledge and practice among indigenous Chepang school children of Nepal. BMC Oral Health 13:1–5 23672487 10.1186/1472-6831-13-20 PMC3655880

[117] De La Quintero TE, Santana Pérez YG, Suárez Gómez IY et al (2022) Condiciones de salud enfermedad bucal en adolescentes embarazadas indígenas y no indígenas de Maracaibo-Venezuela. Revista Estomatológica Herediana 32:381–389. 10.20453/reh.v32i4.4379

[118] Schamschula RG, Cooper MH, Wright MC et al (1980) Oral health of adolescent and adult Australian Aborigines. Community Dent Oral Epidemiol 8:370–374. 10.1111/j.1600-0528.1980.tb01310.x 6937284 10.1111/j.1600-0528.1980.tb01310.x

[119] Schluter PJ, Lee M (2016) Water fluoridation and ethnic inequities in dental caries profiles of New Zealand children aged 5 and 12–13 years: analysis of national cross-sectional registry databases for the decade 2004–2013. BMC Oral Health 16:1–10 26887965 10.1186/s12903-016-0180-5 PMC4758003

[120] Seow WK, Amaratunge A, Bennett R et al (1996) Dental health of Aboriginal pre-sehools children in Brisbane. Australia Community Dent Oral Epidemiol 24:187–190 8871017 10.1111/j.1600-0528.1996.tb00839.x

[121] Seow WK, Amaratunge A, Sim R, Wan A (1999) Prevalence of caries in urban Australian aborigines aged 1-3.5 years. Pediatr Dent 21:91–96 10197332

[122] Shen A, Zeng X, Cheng M et al (2015) Inequalities in dental caries among 12-year‐old Chinese children. J Public Health Dent 75:210–217 25753770 10.1111/jphd.12091

[123] Shi C, Faris P, McNeil DA et al (2018) Ethnic disparities in children’s oral health: Findings from a population-based survey of grade 1 and 2 schoolchildren in Alberta, Canada. BMC Oral Health 18. 10.1186/s12903-017-0444-8 10.1186/s12903-017-0444-8 PMC5753483 29301577

[124] Ship II (1966) Dental caries incidence in North and South Dakota Indian school children during 30 years. J Dent Res 45:359–363 4380017 10.1177/00220345660450022401

[125] Simangwa LD, Åstrøm AN, Johansson A et al (2018) Oral diseases and socio-demographic factors in adolescents living in Maasai population areas of Tanzania: a cross-sectional study. BMC Oral Health 18:1–14 30514291 10.1186/s12903-018-0664-6 PMC6278057

[126] Singh A, Purohit B, Sequeira P, Acharya S (2011) Oral health status of 5-year-old Aborigine children compared with similar aged marginalised group in south western India. Int Dent J 61:157–162. 10.1111/j.1875-595X.2011.00033.x 21692787 10.1111/j.1875-595X.2011.00033.x PMC9374806

[127] Smith L, Blinkhorn A, Moir R et al (2015) An assessment of dental caries among young Aboriginal children in New South Wales, Australia: A cross-sectional study. BMC Public Health 15. 10.1186/s12889-015-2673-6 10.1186/s12889-015-2673-6 PMC4696270 26715325

[128] Tsai WT, Lawrence HP (2022) Association between psychosocial determinants of adverse childhood experiences and severe early childhood caries among First Nations children. Int J Paediatr Dent 32:352–366. 10.1111/ipd.12891 34358378 10.1111/ipd.12891

[129] Zander A, Sivaneswaran S, Skinner J et al (2013) Risk factors for dental caries in small rural and regional Australian communities. Rural Remote Health 13:141–149 23937258

[130] Zeng X, Luo Y, Du M, Bedid R (2005) Dental caries experience of preschool children from different ethnic groups in Guangxi Province in China. Oral Health Prev Dent 3(1):25–31 15921334

[131] Fischman SL (1974) Oral health in the republic of Paraguay. Community Dent Oral Epidemiol 2:176–181 4154820

[132] de la Maza FJ, Cueto MV (1989) Epidemiological study of oral health in a young adult Mapuche population. Odontol Chil 37:183–185 2641955

[133] Yerex K, Lee J, Schroth RJ et al (2025) Children’s Oral Health Initiative program’s impact on First Nations and Inuit children. Can J Dent Hygiene 59:9 PMC11961063 40170948

[134] Jamieson LM, Elani H, Mejia GC et al (2016) Inequalities in indigenous oral health: Findings from Australia, New Zealand, and Canada. J Dent Res 95:1375–1380. 10.1177/0022034516658233 27445131 10.1177/0022034516658233

[135] Nascimento S, Scabar L (2008) Levantamento epidemiológico de cárie, utilizando os índices CPO-D, ceo-d e IHOS, nos índios da aldeia Wakri no Estado do Pará. J Health Sci Inst

[136] Beltrán V, Muñoz-Sepúlveda F, Acevedo C et al (2024) A rural teledentistry care experience: a geriatric approach to assessing oral health status and treatment needs in older adults from a Mapuche community in Chile. Front Public Health 12. 10.3389/fpubh.2024.1356622 10.3389/fpubh.2024.1356622 PMC11188396 38903581

[137] Bongo AKS, Brustad M, Jönsson B (2021) Caries experience among adults in core Sámi areas of Northern Norway. Community Dent Oral Epidemiol 49:401–409. 10.1111/cdoe.12613 33340157 10.1111/cdoe.12613

[138] Soares GH, Aragão AS, Frias AC et al (2019) Epidemiological profile of caries and need for dental extraction in a Kaingang adult Indigenous population. Revista Brasileira de Epidemiologia 22:e190042. 10.1590/1980-549720190042 31432986 10.1590/1980-549720190042

[139] Amarasena N, Kapellas K, Skilton MR et al (2015) Associations with dental caries experience among a convenience sample of Aboriginal Australian adults. Aust Dent J 60:471–478. 10.1111/adj.12256 25424438 10.1111/adj.12256

[140] Aquino-Canchari CR, Caro-Aylas HW, Crisol-Deza DA et al (2019) Clinical epidemiological profile of oral health in peruvian native communities. Revista Habanera de Ciencias Médicas 18:907–919

[141] Traebert JL, Peres MA, Galesso ER et al (2001) Prevalência e severidade da cárie dentária em escolares de seis e doze anos de idade. Rev Saude Publica 35:283–288 11486152 10.1590/s0034-89102001000300011

[142] Ferraro M, Vieira AR (2010) Explaining gender differences in caries: a multifactorial approach to a multifactorial disease. Int J Dent 2010:649643 10.1155/2010/649643 PMC2840374 20339488

[143] Russell SL, Gordon S, Lukacs JR, Kaste LM (2013) Sex/Gender differences in tooth loss and edentulism: historical perspectives, biological factors, and sociologic reasons. Dent Clin 57:317–337 10.1016/j.cden.2013.02.006 23570808

[144] Lukacs JR (2011) Sex differences in dental caries experience: clinical evidence, complex etiology. Clin Oral Investig 15:649–656 20652339 10.1007/s00784-010-0445-3

[145] Crocetti AC, Cubillo B, Lock M et al (2022) The commercial determinants of Indigenous health and well-being: a systematic scoping review. BMJ Glob Health 7:e010366 36319033 10.1136/bmjgh-2022-010366 PMC9628540

[146] Brito ACM, Bezerra IM, Cavalcante D, de FB et al (2020) Dental caries experience and associated factors in 12-year-old-children: a population based-study. Braz Oral Res 34:e010 32049111 10.1590/1807-3107bor-2020.vol34.0010

[147] Martin-Iverson N, Phatouros A, Tennant M (1999) A brief review of indigenous Australian health as it impacts on oral health. Aust Dent J 44:88–92 10452162 10.1111/j.1834-7819.1999.tb00206.x

[148] Sheiham A, James WPT (2015) Diet and dental caries: the pivotal role of free sugars reemphasized. J Dent Res 94:1341–1347 26261186 10.1177/0022034515590377

[149] Malli A, Monteith H, Hiscock EC et al (2023) Impacts of colonization on Indigenous food systems in Canada and the United States: a scoping review. BMC Public Health 23:2105 37885000 10.1186/s12889-023-16997-7 PMC10601184

[150] Olstad DL, Nejatinamini S, Blanchet R et al (2023) Protecting traditional cultural food practices: trends in diet quality and intake of ultra-processed foods by indigenous status and race/ethnicity among a nationally representative sample of adults in Canada. SSM-Population Health 24:101496 37701069 10.1016/j.ssmph.2023.101496 PMC10493595

[151] The Lancet Series (2023) Commercial determinants of health - Summary report., VicHealth

[152] Wood B, Baker P, Sacks G (2021) Conceptualising the commercial determinants of health using a power lens: a review and synthesis of existing frameworks. Int J Health Policy Manag 11:1251 33619932 10.34172/ijhpm.2021.05 PMC9808328

[153] Baker P, Machado P, Santos T et al (2020) Ultra-processed foods and the nutrition transition: Global, regional and national trends, food systems transformations and political economy drivers. Obes Rev 21:e13126 32761763 10.1111/obr.13126

[154] Cascaes AM, da Silva NRJ, dos Santos Fernandez M et al (2023) Ultra-processed food consumption and dental caries in children and adolescents: a systematic review and meta-analysis. Br J Nutr 129:1370–1379 35894293 10.1017/S0007114522002409

[155] Richards Z, Thomas SL, Randle M, Pettigrew S (2015) Corporate social responsibility programs of big food in Australia: a content analysis of industry documents. Aust N Z J Public Health 39:550–556 26259972 10.1111/1753-6405.12429

[156] Arantes R, Santos RV, Frazão P (2010) Oral health in transition: the case of Indigenous peoples from Brazil. Int Dent J 60:235–240 20718309

[157] Vukovic A, Schmutz KA, Borg-Bartolo R et al (2025) Caries status in 12‐year‐old children, geographical location and socioeconomic conditions across European countries: A systematic review and meta‐analysis. Int J Paediatr Dent 35:201–215 38881267 10.1111/ipd.13224 PMC11626496

[158] Baker SR, Foster Page L, Thomson WM et al (2018) Structural determinants and children’s oral health: a cross-national study. J Dent Res 97:1129–1136 29608864 10.1177/0022034518767401

[159] Jamieson LM, Armfield JM, Roberts-Thomson KF (2021) Challenges in identifying indigenous peoples in population oral health surveys: a commentary. BMC Oral Health 21:216 33910554 10.1186/s12903-021-01455-w PMC8082663

[160] Skeie MS, Raadal M, Strand GV, Espelid I (2006) The relationship between caries in the primary dentition at 5 years of age and permanent dentition at 10 years of age–a longitudinal study. Int J Paediatr Dent 16:152–160 16643535 10.1111/j.1365-263X.2006.00720.x

[161] Powell LV (1998) Caries prediction: a review of the literature. Community Dent Oral Epidemiol 26:361–371 9870535 10.1111/j.1600-0528.1998.tb01974.x

[162] Uribe SE, Innes N, Maldupa I (2021) The global prevalence of early childhood caries: a systematic review with meta-analysis using the WHO diagnostic criteria. Int J Paediatr Dent 31:817–830 33735529 10.1111/ipd.12783

[163] Bullen J, Hill-Wall T, Anderson K et al (2023) From deficit to strength-based Aboriginal health research—moving toward flourishing. Int J Environ Res Public Health 20:5395 37048008 10.3390/ijerph20075395 PMC10094537

