# Supplementary material for: Dental caries experience in Indigenous children and adolescents: a systematic review and meta-analysis assessing worldwide disparities and time trends (1968–2024)
Source: Clin Oral Investig. 2026 Jul 29;30(8):365. doi: 10.1007/s00784-026-07047-8 (PMC13415286; doi:10.1007/s00784-026-07047-8)

SUPPLEMENTARY MATERIAL

Clinical Oral Investigations

Dental caries experience in Indigenous children and adolescents: A systematic review and meta-analysis assessing worldwide disparities and time trends (1968–2024)

André Luiz Martins[1,2,4], Edmarlon Girotto[1]*, Junior César de Souza Benedito[1], Pablo Guilherme Caldarelli[1], Kely Barboza Ribeiro[1], Elis Regina Jacintho[2], Sara Carolina Scremin Souza[3], Vicente Martínez-Vizcaíno[4,5,6], Marco Aurelio Anselmo Peres[7,8], Arthur Eumann Mesas[4,6]

[1] Postgraduate Program in Public Health, Universidade Estadual de Londrina, Londrina, Brazil.

[2] Special Indigenous Sanitary District of the Southern Coast, Indigenous Health Secretariat, Ministry of Health, Curitiba, Brazil.

[3] Ottawa Hospital Research Institute, Ottawa, Canada.

[4] Health and Social Research Center, Universidad de Castilla-La Mancha, Cuenca, Spain.

[5] Facultad de Ciencias de la Salud, Universidad Autónoma de Chile, Talca, Chile.

[6] Institute for Health Research of Castilla-La Mancha (IDISCAM), Toledo, Spain.

[7] National Dental Research Institute Singapore, National Dental Centre, Singapore.

[8] Duke-NUS Medical School, Singapore.

***Corresponding author**: Prof. Dr. Edmarlon Girotto, Postgraduate Program in Public Health, Universidade Estadual de Londrina, Avenida Robert Koch, 60, 86039-440, Londrina (PR), Brazil. T.: +55 (43)3371-2919. E-mail: edmarlon@uel.br

Table S1. Search strategies for studies in databases

Database	Syntaxis	Initial search results (up to 03/09/2025)	Updated search results (03/09/2025 – 02/09/2026)
PubMed	("Indigenous peoples"[Title/Abstract] OR "indigen*"[Title/Abstract] OR "aborigin*"[Title/Abstract] OR "amerind*"[Title/Abstract] OR "indian*"[Title/Abstract] OR "tribal"[Title/Abstract] OR "ethnic groups"[Title/Abstract]) AND ("dental caries"[Title/Abstract] OR "dental health surveys"[Title/Abstract] OR "oral health"[Title/Abstract] OR "caries"[Title/Abstract] OR "carious"[Title/Abstract] OR "dental decay"[Title/Abstract] OR "tooth decay"[Title/Abstract] OR "decayed teeth"[Title/Abstract])	1651	106
SciELO	Indigenous peoples (Title) OR indigen* (Title) OR aborigin* (Title) OR amerind* (Title) OR indian* (Title) OR tribal (Title) OR ethnic groups (Title) AND dental caries (Title) OR dental health surveys (Title) OR oral health (Title) OR caries (Title) OR carious (Title) OR dental decay (Title) OR tooth decay (Title) OR decayed teeth (Title)	8113	30
Embase	'dental caries':ab,ti OR 'dental health surveys':ab,ti OR 'oral health':ab,ti OR caries:ab,ti OR carious:ab,ti OR 'dental decay':ab,ti OR 'tooth decay':ab,ti OR 'decayed teeth':ab,ti AND 'Indigenous peoples':ab,ti OR indigen*:ab,ti OR aborigin*:ab,ti OR amerind*:ab,ti OR indian*:ab,ti OR 'ethnic group':ab,ti	1522	115
Scopus	(TITLE-ABS-KEY (Indigenous AND peoples) OR TITLE-ABS-KEY (indigen*) OR TITLE-ABS-KEY (aborigin*) OR TITLE-ABS-KEY (amerind*) OR TITLE-ABS-KEY (indian*) OR TITLE-ABS-KEY (tribal) OR TITLE-ABS-KEY (ethnic AND groups) AND TITLE-ABS-KEY (dental AND caries) OR TITLE-ABS-KEY (dental AND health AND surveys) OR TITLE-ABS-KEY (oral AND health) OR TITLE-ABS-KEY (caries) OR TITLE-ABS-KEY (carious) OR TITLE-ABS-KEY (dental AND decay) OR TITLE-ABS-KEY (tooth AND decay) OR TITLE-ABS-KEY (decayed AND teeth))	7107	485

Table S2. Articles excluded and reasons for exclusion.

Authors (year)	Title	Reason for exclusion*
Aamodt et al. (2015)	Prevalence of caries and malocclusion in an Indigenous population in Chiapas, Mexico	3
Amarasena et al. (2015)	Associations with dental caries experience among a convenience sample of Aboriginal Australian adults	7
Aquino-Canchari et al. (2019)	Clinical epidemiological profile of oral health in peruvian native communities	7
Beltrán et al. (2024)	A rural teledentistry care experience: a geriatric approach to assessing oral health status and treatment needs in older adults from a Mapuche community in Chile	7
Bongo, Brustad & Jönsson (2021)	Caries experience among adults in core Sámi areas of Northern Norway	7
Brennan, Roberts-Thomson, Spencer (2007)	Oral health of Indigenous adult public dental patients in Australia	7
Cantarutti et al. (2025)	Social determinants of oral health in an Indigenous community of Chile: preliminary data of a mixed qualitative and quantitative study	7
Kapellas et al. (2014)	Periodontal disease and dental caries among Indigenous Australians living in the Northern Territory, Australia.	7
Kruger et al. (2008)	The oral health status and treatment needs of Indigenous adults in the Kimberley region of Western Australia	7
Montanha-Andrade et al. (2019)	Dental health status and its indicators in adult Brazilian Indians without exposition to drinking water fluoridation: a cross-sectional study	7
Smith et al. (2007)	Oral health in rural and remote Western Australian Indigenous communities: a two-year retrospective analysis of 999 people	7
Soares et al. (2019)	Epidemiological profile of caries and need for dental extraction in a Kaingang adult Indigenous population	7
Coelho et al. (2021)	Recommendation of tooth extraction and associated factors: cross-sectional study in the Kiriri Indigenous population	3
Macnab et al. (2008)	3-year results of a collaborative school-based oral health program in a remote First Nations community	6
Rao, Bharambe (1993)	Dental caries and periodontal diseases among urban, rural and tribal school children	5
Lin, Chu (1997)	A survey on dental caries in schoolchildren on Lanyu Island	4
Mejia, Parker, Jamieson (2010)	An introduction to oral health inequalities among Indigenous and non-Indigenous populations	1
Singh et al. (2020)	Assessment of oral health status and treatment needs among people of Foklyan area, Dharan, Nepal	4
Ribeiro et al. (2016)	Association of dental infections with systemic diseases in Brazilian Native Indigenous: a cross-sectional study	3
Arrow et al. (2022)	Atraumatic restorative treatments and oral health-related quality of life and dental anxiety in Australian Aboriginal children: A cluster-randomized trial	6
Arrow et al. (2021)	Atraumatic Restorative Treatments in Australian Aboriginal Communities: A Cluster-randomized Trial	6

Ricomini Filho et al. (2021)	Community interventions and strategies for caries control in Latin American and Caribbean countries	1
Moffatt (1995)	Current status of nutritional deficiencies in Canadian aboriginal people	2
Davies et al. (1997)	Dental caries among Australian Aboriginal, non-Aboriginal Australian-born, and overseas-born children	3
Cruz Palma et al. (2014)	Dental caries and social determinants of health in Mexico	3
Khan, Cleaton-Jones (1998)	Dental caries in African preschool children: social factors as disease markers	3
Harrison, Veronneau, Leroux (2010)	Design and implementation of a dental caries prevention trial in remote Canadian Aboriginal communities.	6
Baghdadi (2016)	Early Childhood Caries and Indigenous Children in Canada: Prevalence, Risk Factors, and Prevention Strategies	1
Ju et al. (2024)	Early Childhood Caries Intervention in Aboriginal Australian Children: A Cross-in Randomized Trial.	6
Chen et al. (2011)	Educational intervention can improve dental care knowledge in aboriginal tribal children	5
Harrison, Veronneau, Leroux (2012)	Effectiveness of maternal counseling in reducing caries in Cree children	6
Ju, Jamieson, Mejia (2016)	Estimating the effects of maternal education on child dental caries using marginal structural models: The Longitudinal Study of Indigenous Australian Children	3
Agudelo-Suárez et al. (2014)	Ethnicity and self-perceived oral health in Colombia: a cross-sectional analysis	3
Ellenikiotis et al. (2015)	Examining the link between prevalence of severe early childhood caries and self-reported oral health and quality of life outcomes	1
Jamieson et al. (2019)	Follow-up of an Intervention to Reduce Dental Caries in Indigenous Australian Children: A Secondary Analysis of a Randomized Clinical Trial	6
Soares et al. (2021)	Household food insecurity, dental caries and oral-health-related quality of life in Brazilian Indigenous adults	2
Jamieson, Parker, Armfield (2007)	Indigenous child oral health at a regional and state level	5
Wang et al. (2024)	Inequalities in dental caries among Indigenous and non-Indigenous children in Australia: A literature review	1
Drummond et al. (2015)	Inequality of Experience of Dental Caries between Different Ethnic Groups of Brazilians Aged 15 to 19 Years	2
Haag et al. (2019)	Is There an Association between Breastfeeding and Dental Caries among Three-Year-Old Australian Aboriginal Children?	6
Vega Lizama, Cucina (2014)	Maize dependence or market integration? Caries prevalence among Indigenous Maya communities with maize-based versus globalized economies	5
Lemos et al. (2018)	Oral health care in the Xingu Indigenous Park, Brazil, from 2004 to 2013: an analysis based on evaluation indicators	3
Parker, Jamieson (2007)	Oral health comparisons between children attending an Aboriginal health service and a Government school dental service in a regional location	4
Brustad et al. (2020)	Oral health in the Indigenous Sámi population in Norway - the dental health in the North study	3
Arantes, Santos, Frazão (2010)	Oral health in transition: the case of Indigenous peoples from Brazil	3

Jamieson, Armfield, Roberts-Thomson (2006)	Oral health inequalities among Indigenous and non-Indigenous children in the Northern Territory of Australia	3
Haag et al. (2021)	Oral Health Inequalities among Indigenous and Non-Indigenous Children	3
Kilpatrick et al. (2012)	Oral health inequalities in a national sample of Australian children aged 2-3 and 6-7 years	3
Kapellas et at. (2021)	Oral health of aboriginal people with kidney disease living in Central Australia	3
Dowsett, Archila, Kowolik (2001)	Oral health status of an Indigenous adult population of Central America	3
Kailis, Silva (1971)	Prevalence of dental caries in Australian aboriginal children resident in Carnarvon, Western Australia	3
Do et al. (2018)	Race- and Income-Related Inequalities in Oral Health in Australian Children by Fluoridation Status	3
Jordão, Malta, Freire (2018)	Simultaneous oral health risk behaviors among adolescents: evidence from the National School-based Student Health Survey	3
Alves Filho, Santos, Vettore (2013)	Social and environmental inequities in dental caries among Indigenous population in Brazil: evidence from 2000 to 2007	1
Ferro et al. (2010)	Social differences in tooth decay occurrence in a sample of children aged 3 to 5 in north-east Italy	2
Ha et al. (2016)	Social inequality in dental caries and changes over time among Indigenous and non-Indigenous Australian children	5
Findlay, Janz (2012)	The health of Inuit children under age 6 in Canada	2
Schuch et al. (2017)	The magnitude of Indigenous and non-Indigenous oral health inequalities in Brazil, New Zealand and Australia	3
Moreira, Lima (2015)	The Oral Health in Brazilian Indigenous: The Presence of Permanent Teeth in Elderlies and Adults Belonging to an Indigenous Population in the Northeast, Brazil	1
Claudia et al. (2016)	The relationship between maternal smoking during pregnancy and parental-reported experience of dental caries in Indigenous Australian children	3
Silveira et al. (2015)	Tooth decay and associated factors among adolescents in the north of the State of Minas Gerais, Brazil: a hierarchical analysis	3
Ha et al. (2016)	Trends in caries experience and associated contextual factors among Indigenous children	3
Lopez et al. (2023)	Trends in social inequalities in early childhood caries using population-based clinical data	3
Neel et al. (1964)	Studies on the Xavante Indians of the Brazilian Mato Grosso	9
Donnelly et al. (1977)	Plaque, caries, periodontal diseases, and acculturation among Yanomamö Indians, Venezuela	9
Kieser, Preston (1984)	Oral health status of the Lengua Indians of Paraguay	9
Biazevic, Fantin Michel-Crosato (2005)	Oral health conditions survey among 6-to12-year-old children living in the indigenous reserve of Cacique Doble, Rio Grande do Sul, Brazil	1
Diab, Lucas (2008)	Cárie dental em crianças indígenas Xakriabá	1
Ju et al. (2025)	Early childhood caries intervention in Aboriginal Australian children: Follow-up at child age 9 years	6
Garza, Miguel (2025)	Health disparities among indigenous populations in Latin America: a scoping review	2

Roman-Torres et al. (2025)	Comparison of oral conditions between the Macuxi and Yanomami indigenous ethnicities	7
Kolahdooz et al. (2025)	Oral Health Practices Among Indigenous Mothers and Young Children (0-36 Months) in Northwest Territories, Canada	1
Armas-Veja et al. (2025)	Association Between Dental Caries, Dental Biofilm, and Body Mass Index in Indigenous Children from Two Regions of Ecuador: A Cross-Sectional Study	3
Lopez et al. (2025)	Socio-Educational Factors Associated With the Caries Index in Indigenous Amazonian Nationalities	3
Jaramillo et al. (2025)	Epidemiological Profile of Oral Health Conditions in Ecuador: A Retrospective Study From 2016 to 2022	3
Cantarutti et al. (2025)	Social determinants of oral health in an indigenous community of Chile: preliminary data of a mixed qualitative and quantitative study	7
Tamchos et al. (2025)	Prevalence of early childhood caries among preschool children of tribal Leh district of Ladakh: A cross-sectional study	3
Raymundo et al. (2025)	Oral health surveys of traditional peoples and communities in Brazil: A scope review	2
Williams et al. (2025)	Relationship between Safety-net Oral Health Care Practices and Income among American Indian/Alaska Native, Black, and Hispanic/Latino Dentists	1
Singh et al. (2025)	Social inequities in early childhood caries in Australia: A population-based study on statewide public dental services data	3

The reasons for exclusion were as follows: (1) nonprimary research articles, including systematic reviews, meta-analyses, narrative reviews, editorials, commentaries, conference abstracts, and case reports; (2) studies that did not address dental caries in Indigenous populations as the primary outcome; (3) studies using non-standardized caries indices or composite measures that could not be disaggregated into dmft or DMFT values; (4) mixed-population studies that included both Indigenous and non-Indigenous participants, unless they provided results stratified by ethnicity or Indigenous status; (5) studies reporting aggregate data across dentition types that combine deciduous and permanent teeth without providing individual dmft and DMFT indices; (6) intervention studies, including randomized controlled trials and quasiexperimental designs, as the research focus was observational prevalence data rather than treatment effects; (7) studies that included participants outside the 5–19 age range, unless age-stratified reporting allowed extraction of data for the target age group; (8) duplicate publications reporting identical datasets (preference was given to the most recent or methodologically comprehensive version, as the research focus was on double-counting study populations); and (9) no data interest.

Table S3. Assessment of the methodological quality of studies selected using the Joanna Briggs Institute instrument.

**Authors (year)**	Were the criteria for inclusion in the sample clearly defined?	Were the study subjects and the setting described in detail?	Were objective, standard criteria used for measurement of the condition?	Were confounding factors identified?	Were strategies to deal with confounding factors stated?	Was appropriate statistical analysis used?
Abdul Kadir, Adnan (1989)	Yes	No	Unclear	Yes	Yes	Yes
Alves Filho, Santos, Vettore (2009)	Yes	Yes	Yes	Yes	Yes	Yes
Arantes & Frazão (2016)	Yes	Yes	Yes	Yes	Yes	Yes
Arantes, Jamieson & Frazão (2021)	Yes	Yes	Yes	Yes	Yes	Yes
Arantes et al. (2009)	Yes	Yes	Yes	Yes	Yes	Yes
Arantes, Santos, Coimbra Jr. (2001)	Yes	Yes	Yes	Yes	Yes	Yes
Arrow, (2016)	Yes	Yes	Unclear	Yes	Yes	Yes
Baldisserotto, Ferreira & Warmling (2019)	Yes	Yes	Yes	Yes	Yes	Yes
Barrett et al. (1972)	Yes	Yes	Yes	Yes	Unclear	Yes
Batliner et al. (2016)	Yes	Yes	Yes	Yes	Yes	Yes
Berhan Nordin et al. (2019)	Yes	Yes	Yes	Yes	Unclear	Yes
Butten et al. (2019)	Yes	Unclear	No	Yes	Yes	Yes
Caires et al. (2018)	Yes	Yes	Unclear	Yes	Yes	Yes
Carneiro et al. (2008)	Unclear	Yes	Yes	Yes	Yes	Yes
Chang (1971)	Unclear	Unclear	Unclear	Yes	Yes	Yes
Chinnakotla et al. (2023)	Yes	Yes	Yes	Yes	Yes	Yes
D´Mello, et al. (2011)	Yes	Yes	Unclear	Yes	Yes	Yes
Dasanayake, Caufield (2002)	Yes	Yes	Yes	Yes	Unclear	Yes
Davies et al. (1997)	Yes	Yes	No	Yes	Yes	Yes
de la Maza, Cueto	Yes	Yes	Yes	Yes	No	Unclear
de Muññiz (1985)	Yes	No	Yes	Yes	Yes	Yes
del Rio Gomez (1991)	Yes	No	No	Yes	Yes	Yes
Dimitropoulos et al. (2018)	Yes	Yes	No	Yes	Yes	Yes
Dogar et al. (2011)	Yes	No	Yes	Yes	Yes	Yes
Drummond et al. (2015)	Yes	Yes	Yes	Yes	Yes	Yes
Endean et al. (2004)	Yes	Yes	No	Yes	Yes	Yes
Fischman (1974)	Unclear	Yes	Yes	Yes	No	Yes
Gonçalves et al. (2015)	Yes	Yes	Yes	Yes	Yes	Yes
Gowda et al. (2009)	Unclear	No	No	Yes	Yes	Yes
Grim et al. (1994)	Yes	Yes	Yes	Yes	Yes	Yes
Guisilini et al. (2021)	Yes	Yes	Yes	Yes	Yes	Yes
Ha (2014)	Yes	Yes	Unclear	Yes	Yes	Yes
Ha, Crocombe, Mejia (2014)	Yes	Yes	No	Yes	Yes	Yes
Ha et al. (2016)	Unclear	Yes	Unclear	Yes	Yes	Yes
Hagens et al. (2023)	Yes	Yes	Yes	Yes	Unclear	Yes
Hallett, O'Rourke (2002)	Yes	Yes	Yes	Yes	Yes	Yes
Hirata et al. (1997)	Yes	No	Unclear	Yes	Yes	Yes
Hirooka et al. (2014)	Yes	Yes	Yes	Yes	Yes	Yes
Homan, Davies (1973)	Yes	No	Yes	Yes	Unclear	Yes
Hopcraft, Chow (2007)	Yes	Yes	Yes	Yes	Yes	Yes
Jamieson, Armfield, Roberts-Thomson (2007)	Yes	Yes	Unclear	Yes	Yes	Yes
Jamieson, Armfield, Roberts-Thomson (2007)	Yes	Yes	Unclear	Yes	Yes	Yes
Jamieson, Roberts-Thomson, Sayers (2010)	Yes	Yes	Unclear	Yes	Yes	Yes
Jamieson et al. (2013)	Yes	Yes	Yes	Yes	Yes	Yes

Jamieson, Sayers, Roberts-Thomson (2013)	Yes	Yes	Yes	Yes	Yes	Yes
Jayashantha & Johnson (2016)	Yes	Yes	No	Yes	Yes	Yes
Jamieson et al. (2021)	Yes	Yes	Yes	Yes	Yes	Yes
Jamieson, Armfield, Roberts-Thomson (2006)	Yes	Yes	Unclear	Yes	Yes	Yes
Jamieson et al. (2010)	Yes	Yes	Unclear	Yes	Yes	Yes
Jayashantha & Johnson (2016)	Yes	Yes	No	Yes	Unclear	Yes
John et al. (2015)	Yes	No	No	Yes	Yes	Yes
Johnson et al. (2014)	Yes	Yes	Yes	Yes	Yes	Yes
Jones et al. (1992)	Yes	No	Yes	Yes	Yes	Yes
Kadir et al. (1990)	Yes	Yes	Yes	Yes	Yes	Yes
Kailis (1979)	Yes	Yes	No	Yes	Yes	Yes
Koike et al. (2024)	Yes	No	No	Yes	Unclear	Yes
Kroon et al. (2019)	Yes	Yes	Yes	Yes	Yes	Yes
Kruger, Dyson, Tennant (2005)	Yes	Yes	No	Yes	Yes	Yes
Kumar et al. (2013)	Yes	No	No	Yes	Unclear	Yes
Lalloo et al. (2016)	Unclear	Yes	Unclear	Yes	Yes	Yes
Lawrence et al. (2009)	Yes	Yes	Yes	Yes	Yes	Yes
Lee et al. (2022)	Yes	Yes	Yes	Yes	Yes	Yes
Lemos et al. (2018)	Yes	Yes	Yes	Yes	Yes	Yes
Mauricio & Moreira (2014)	Yes	Yes	Yes	Yes	Unclear	Yes
Mauricio, Fávaro, Moreira (2024)	Yes	Yes	Yes	Yes	Yes	Yes
Medina et al. (2008)	Yes	Yes	No	Yes	Yes	Yes
Miranda, Souza, Leal (2018)	Yes	Yes	Yes	Yes	Yes	Yes
Miyazaki, Takehara (1988)	Yes	Yes	Unclear	Yes	Yes	Yes
Nascimento, Scabar (2008)	Yes	Yes	Yes	Yes	Unclear	Yes
Niendorff, Jones (2000)	Unclear	Yes	No	Yes	Yes	Yes
Oliveira et al. (2024)	Yes	No	Yes	Yes	Yes	Yes
Peressini et al. (2004)	Yes	Yes	No	Yes	Yes	Yes
Peressini et al. (2004)	Yes	Yes	Yes	Yes	Yes	Yes
Phipps et al. (2012)	Yes	Yes	No	Yes	Yes	Yes
Poni et al. (2023)	Yes	No	Yes	Yes	Yes	Yes
Prasai Dixit et al. (2013)	Yes	Yes	Yes	Yes	Yes	Yes
Quintero De La Hoz (2022)	Yes	Yes	Yes	Yes	Unclear	Yes
Rigonatto, Antunes, Frazão (2001)	Yes	Yes	Unclear	Yes	Yes	Yes
Sampaio et al. (2010)	Yes	No	Yes	Yes	Yes	Yes
Schamschula et al. (1980)	Yes	No	No	Yes	Unclear	Yes
Schroth, Moore, Brothwell (2005)	Yes	Yes	No	Yes	Yes	Yes
Schuluter, Lee (2016)	Yes	Yes	Yes	Yes	Yes	Yes
Seow et al. (1996)	Yes	No	No	Yes	Unclear	Yes
Seow et al. (1999)	Yes	Yes	Yes	Yes	Yes	Yes
Shen et al. (2015)	Yes	Yes	Yes	Yes	Yes	Yes
Shi et al. (2018)	Yes	Yes	Yes	Yes	Yes	Yes
Ship (1966)	Yes	Yes	No	Yes	Yes	Yes
Simangwa et al. (2018)	Yes	Yes	Yes	Yes	Yes	Yes
Singh et al. (2011)	Yes	Yes	Yes	Yes	Yes	Yes
Smith et al. (2015)	Yes	Yes	No	Yes	Yes	Yes
So et al. (2017)	Yes	Yes	No	Yes	Unclear	Yes
Tsai & Lawrence (2022)	Yes	Yes	Yes	Yes	Yes	Yes
Yerex et al. (2025)	Yes	Yes	Yes	Yes	Yes	Yes
Zander et al. (2013)	Yes	Yes	Yes	Yes	Yes	Yes
Zeng et al. (2005)	Yes	No	Yes	Yes	Yes	Yes

Table S4. Summary of the assessment of the methodological quality of studies selected using the Joanna Briggs Institute instrument.

**Criterion**	**Yes**	**No**	**Unclear**
	**n (%)**	**n (%)**	**n (%)**
Clearly defined inclusion criteria?	88 (92.6)	-	7 (7.4)
Subjects and scenarios described in detail?	76 (80.0)	17 (17.9)	2 (2.1)
Objective criteria used for measurement?	56 (57.9)	24 (25.3)	16 (16.8)
Confounding factors identified?	95 (100.0)	-	-
Strategies for dealing with stated confounders?	79 (83.2)	2 (2.1)	14 (14.7)
Appropriate statistical analysis used?	94 (98.9)	-	1 (1.1)

Table S5. Sensitivity analysis performed by removing studies one by one from the pooled analysis of the dmft index (deciduous dentition) of Indigenous children.

Study omitted	Estimate	Lower CI	Upper CI
Tsai et al. (2021)	3.7479455	3.5794649	3.9164262
Lemos et al. (2018)[1]	3.7682073	3.5986779	3.9377365
Lemos et al. (2018)[2]	3.7421670	3.5738411	3.9104927
Guisilini et al. (2021)	3.7567160	3.5877154	3.9257166
Ha et al. (2016)[3]	3.7199647	3.5525560	3.8873732
Ha et al. (2016)[4]	3.7540123	3.5853038	3.9227209
Ha et al. (2014)	3.7418222	3.5734181	3.9102266
Baldisserotto et al. (2019)	3.7098308	3.5427387	3.8769231
Gonçalves et al. (2015)	3.7602723	3.5919337	3.9286108
Arantes et al. (2021)	3.7317953	3.5640321	3.8995583
Ha (2014)	3.7806895	3.6118271	3.9495516
Lee et al. (2022)	3.7466271	3.5784173	3.9148369
Jamieson et al. (2010)	3.7610779	3.5920367	3.9301190
Smith et al. (2015)	3.7705863	3.6014206	3.9397519
Kailis (1979)	3.6961772	3.5310402	3.8613141
Miranda et al. (2018)	3.7604001	3.5910168	3.9297836
Sampaio et al. (2010)	3.7213910	3.5536940	3.8890877
Hopcraft et al. (2007)	3.7772274	3.6083295	3.9461250
Kroon et al. (2019)[5]	3.7486608	3.5800345	3.9172873
Kroon et al. (2019)[6]	3.7550778	3.5863681	3.9237876
Kroon et al. (2019)[7]	3.7717757	3.6007638	3.9427876
Kroon et al. (2019)[8]	3.7641780	3.5935855	3.9347703
Kroon et al. (2019)[9]	3.7615583	3.5913844	3.9317322
Rigonatto et al. (2001)	3.7730739	3.6022518	3.9438963
Gowda et al. (2009)	3.7692759	3.5982690	3.9402828
Seow et al. (1996)	3.7637932	3.5935009	3.9340856
Arantes et al. (2001)	3.7054381	3.5390897	3.8717866
Lawrence et al. (2009)[10]	3.7486455	3.5800097	3.9172812
Lawrence et al. (2009)[11]	3.7672613	3.5976491	3.9368737
Lawrence et al. (2009)[12]	3.7453108	3.5773425	3.9132791
Dogar et al. (2011)	3.7365034	3.5685196	3.9044871
Singh et al. (2011)	3.7437625	3.5765421	3.9109831
Hagens et al. (2023)	3.7626994	3.5944757	3.9309230
Kruger et al. (2005)	3.7359257	3.5678298	3.9040217
Peressini et al. (2004)[13]	3.7285075	3.5609517	3.8960633
Peressini et al. (2004)[14]	3.7347183	3.5668731	3.9025633
Oliveira et al. (2024)	3.7301323	3.5621903	3.8980744
Butten et al. (2019)	3.7377746	3.5694914	3.9060576
Alves Filho et al. (2009)	3.7281921	3.5604019	3.8959823
Phipps et al. (2012)	3.7436402	3.5753160	3.9119642
Grim et al. (1994)	3.7201560	3.5526066	3.8877053
Batliner et al. (2016)	3.7254448	3.5578067	3.8930829
Jamieson et al. (2007)[15]	3.7706444	3.5994620	3.9418271
Jamieson et al. (2007)[16]	3.7719798	3.6129735	3.9309862
Jamieson et al. (2007)[17]	3.7635841	3.5934494	3.9337189

Jamieson et al. (2007)[18]	3.7575908	3.5879998	3.9271817
Jamieson et al. (2007)[19]	3.7637932	3.5935009	3.9340856
Jamieson et al. (2007)[20]	3.7539349	3.5850880	3.9227817
Jamieson et al. (2007)[21]	3.7344551	3.5663929	3.9025173
Jamieson et al. (2007)[22]	3.7718480	3.6026533	3.9410424
Jamieson et al. (2007)[23]	3.7666709	3.5975688	3.9357729
Jamieson et al. (2007)[24]	3.7542596	3.5857589	3.9227605
Jamieson et al. (2007)[25]	3.7470877	3.5787814	3.9153941
Jamieson et al. (2007)[26]	3.8265741	3.5805304	4.0726180
Chang (1971)	3.7579124	3.5872965	3.9285281
Arrow (2016)	3.7322226	3.5644016	3.9000437
Hallett et al. (2002)	3.6974697	3.5313876	3.8635521
Jones et al. (1992)	3.7343750	3.5662560	3.9024940
Hirooka et al. (2014)	3.7636845	3.5944335	3.9329357
Chinnakotla et al. (2023)	3.7604353	3.5918002	3.9290705
Schroth et al. (2005)	3.7657330	3.5969951	3.9344707
Kumar et al. (2013)	3.7540503	3.5851586	3.9229417
Zeng et al. (2005)	3.7705739	3.6018353	3.9393122
**Combined**	**3.7495509**	**3.5822018**	**3.916000**

Note: (1) year of data collection: 2007; (2) year of data collection: 2013; (3) period of data collection: 2000-2002; (4) period of data collection: 2007-2010; (5) year of data collection: 2004; (6) year of data collection: 2012; (7) year of data collection: 2015; (8) year of data collection: 2016; (9) year of data collection: 2016; (10) period of data collection: 2003-2004; (11) period of data collection: 2004-2005; (12) period of data collection: 2005-2006; (13) 7 years; (14) 3-5 years; (15) year of data collection: 1989; (16) year of data collection: 1990; (17) year of data collection: 1991; (18) year of data collection: 1992; (19) year of data collection: 1993; (20) year of data collection: 1994; (21) year of data collection: 1995; (22) year of data collection: 1996; (23) year of data collection: 1997; (24) year of data collection: 1998; (25) year of data collection: 1999; (26) year of data collection: 2000.

Table S6. Sensitivity analysis performed by removing studies one by one from the pooled analysis of the DMFT index (permanent dentition) of Indigenous children and adolescents.

Study omitted	Estimate	Lower CI	Upper CI
Lemos et al. (2018)[1]	2.3520885	2.1679816	2.5361953
Lemos et al. (2018)[2]	2.3896277	2.2048340	2.5744214
Ha et al. (2016)[3]	2.3446584	2.1610169	2.5282998
Ha et al. (2016)[4]	2.3322995	2.1493874	2.5152118
Ha et al. (2014)	2.3834155	2.1965220	2.5703089
Baldisserotto et al. (2019)[5]	2.3497198	2.1653478	2.5340917
Gonçalves et al. (2015)	2.3958299	2.2119648	2.5796950
Arantes et al. (2021)[6]	2.3850045	2.1999614	2.5700476
Arantes et al. (2016)	2.3090146	2.1265631	2.4914660
Ha (2014)	2.3544819	2.1700237	2.5389404
Maurício et al. (2014)[7]	2.3685069	2.1838183	2.5531955
Jamieson et al. (2010)	2.3839214	2.1975925	2.5702503
Kailis (1979)	2.3569674	2.1724591	2.5414755
Miranda et al. (2018)[8]	2.3865113	2.2014995	2.5715232
Arantes et al. (2009)[9]	2.3867466	2.2002399	2.5732532
Sampaio et al (2010)[10]	2.3957369	2.2113717	2.5801020
Hopcraft (2007)[11]	2.3378515	2.1536753	2.5220277
Kroon et al. (2019)[12]	2.3772352	2.1921551	2.5623152
Kroon et al. (2019)[13]	2.3125310	2.1391482	2.4859138
Kroon et al. (2019)[14]	2.3876829	2.2013252	2.5740407
Kroon et al. (2019)[15]	2.3881443	2.2002509	2.5760374
Kroon et al. (2019)[16]	2.3814435	2.1956954	2.5671914
Rigonatto et al. (2001)[17]	2.3801041	2.1937215	2.5664864
Gowda et al. (2009)	2.3802729	2.1943204	2.5662253
Medina et al. (2008)	2.3736560	2.1886368	2.5586755
Kadir et al. (1990)	2.3876367	2.2026186	2.5726547
Arantes et al. (2001)[18]	2.3836262	2.1972828	2.5699694
Barrett et al. (1972)[19]	2.3424385	2.1581743	2.5267026
Hagens et al. (2023)	2.3812902	2.1955409	2.5670395
Miyazaki et al. (1988)[20]	2.3833282	2.1965206	2.5701358
Alves Filho et al. (2009)[21]	2.3393862	2.1558275	2.5229449
Niendorff et al.(2000)[22]	2.3890061	2.2019620	2.5760503
Jamieson et al. (2007)[23]	2.3269455	2.1433294	2.5105617
Jamieson et al. (2007)[24]	2.3320777	2.1480050	2.5161505
Jamieson et al. (2007)[25]	2.3782287	2.1928644	2.5635929
Jamieson et al. (2007)[26]	2.3766081	2.1914349	2.5617814
Jamieson et al. (2007)[27]	2.3833783	2.1959069	2.5708497
Jamieson et al. (2007)[28]	2.2997639	2.1169400	2.4825876
Jamieson et al. (2007)[29]	2.3687644	2.1839824	2.5535462
Jamieson et al. (2007)[30]	2.3802400	2.1948662	2.5656140
Jamieson et al. (2007)[31]	2.3818772	2.1969700	2.5667844
Jamieson et al. (2007)[32]	2.3832111	2.1984580	2.5679641
Jamieson et al. (2007)[33]	2.3790884	2.1942778	2.5638990
Jamieson et al. (2007)[34]	2.3655047	2.1808112	2.5501986
Chang (1971)	2.3575342	2.1730416	2.5420265

Arrow (2016)	2.3540392	2.1697695	2.5383091
John et al. (2015)	2.3818886	2.1971378	2.5666397
Shen et al. (2015)	2.3813276	2.1966515	2.5660038
Kumar et al. (2013)	2.3430750	2.1590104	2.5271399
Batliner et al. (2016)	2.3549619	2.1706951	2.5392287
Lemos et al. (2018)[35]	2.3608208	2.1763024	2.5453393
Lemos et al. (2018)[36]	2.3534400	2.1689913	2.5378890
Baldisserotto et al. (2019)[37]	2.3422749	2.1587768	2.5257730
Arantes et al. (2021)[38]	2.3284760	2.1451080	2.5118439
Maurício et al. (2014)[39]	2.3439810	2.1597767	2.5281854
Jamieson et al. (2013)	2.3783796	2.1936817	2.5630774
Miranda et al. (2018)[40]	2.3424554	2.1586192	2.5262916
Arantes et al. (2009)[41]	2.3486388	2.1646073	2.5326705
Sampaio et al (2010)[42]	2.3766131	2.1914361	2.5617902
Hopcraft (2007)[43]	2.3716593	2.1866617	2.5566568
Rigonatto et al. (2001)[44]	2.3878407	2.1964326	2.5792491
Jamieson et al. (2010)	2.4038353	2.2023001	2.6053705
Quintero De La Hoz (2022)	2.4200914	2.1837358	2.6564472
Arantes et al. (2001)[45]	2.3902097	2.2053771	2.5750422
Barrett et al. (1972)[46]	2.3946211	2.2100017	2.5792403
Peressini et al. (2004)	2.3125310	2.1391482	2.4859138
Miyazaki et al. (1988)[47]	2.3530097	2.1694312	2.5365880
Alves Filho et al. (2009)[48]	2.2639151	2.1024001	2.4254301
Niendorff et al. (2000)[49]	2.3748188	2.1872346	2.5624030
Grim et al. (1994)	2.2916789	2.1101954	2.4731622
Jamieson et al. (2013)	2.3444555	2.1601930	2.5287180
**Combined**	**2.363214**	**2.1798814**	**2.5465466**

Note: (1) year of data collection: 2007 / 12 years; (2) year of data collection: 2013 / 12 years; (3) period of data collection: 2000-2002; (4) period of data collection: 2007-2010; (5) 12 years; (6) 12 years; (7) 10-12 years; (8) 5-12 years; (9) 6-12 years; (10) 12 years; (11) 5-12 years; (12) year of data collection: 2004; (13) year of data collection: 2012; (14) year of data collection: 2015; (15) year of data collection: 2016; (16) year of data collection: 2017; (17) 5-13 years; (18) 6-12 years; (19) 5-10 years; (20) 10-12 years; (21) 5-10 and 12 years; (22) 5-13 years; (23) year of data collection: 1989; (24) year of data collection: 1990; (25) year of data collection: 1991; (26) year of data collection: 1992; (27) year of data collection: 1993; (28) year of data collection: 1994; (29) year of data collection: 1995; (30) year of data collection: 1996; (31) year of data collection: 1997; (32) year of data collection; 1998; (33) year of data collection: 1999; (34) year of data collection: 2000; (35) year of data collection: 2007 / 15-19 years; (36) year of data collection: 2013 / 15-19 years; (37) 15-19 years; (38) 13-19 years; (39) 13-14 years; (40) 15-19 years; (41) 13-19 years; (42) 15-19 years; (43) 13-15 years; (44) 14-19 years; (45) 13-19 years; (46) 10-19 years; (47) 13-15 years; (48) 15-19 years; (49) 14-19 years.

Table S7. Egger test results for the included studies on the dmft index (deciduous dentition) in Indigenous children.

Egger's test for small-study effects:							
Regress standard normal deviate of intervention							
effect estimate against its standard error							
Number of studies = 63				Root MSE = 7.815			
**Std_Eff**	**Coefficient**	**Std. err.**	**t**	**P>|t|**		**[95% conf. interval]**	
slope	2.717123	.0428317		63.44	0.000	2.631476	2.802771
bias	4.05357	1.063929		3.81	0.000	1.926111	6.181028
Test of H0: no small-study effects		P = 0.000					

Table S8. Results of the Egger test for the included studies on the DMFT index (permanent dentition) of Indigenous children and adolescents.

Egger's test for small-study effects:							
Regress standard normal deviate of intervention							
effect estimate against its standard error							
Number of studies = 71				Root MSE = 15.58			
**Std_Eff**	**Coefficient**	**Std. err.**	**t**	**P>|t|**		**[95% conf. interval]**	
slope	1.160299	.0839986		13.81	0.000	.9927267	1.327872
bias	6.898948	2.207043		3.13	0.003	2.496017	11.30188
Test of H0: no small-study effects		P = 0.003					

Figure S1. Forest plot of the mean dmft index (deciduous dentition) among Indigenous children.

Authors	Country	Global region	Year of data colletion	HDI	Sample size	Mean age	% Female	Effect (95% CI)	% Weight
Alves Filho et al. (2009)	BRA	Latin America	2006	0.722	246	6.8	48.6	2.00 (1.63, 2.37)	1.75
Arantes et al. (2001)	BRA	Latin America	1997	0.668	105	7.0	53.0	3.47 (2.89, 4.05)	1.55
Arantes et al. (2021)	BRA	Latin America	2013	0.750	606	5.0	NR	3.20 (2.95, 3.45)	1.84
Arrow (2016)	AUS	Oceania and Asia	2014	0.924	268	7.5	47.6	2.54 (2.21, 2.87)	1.78
Baldisserotto et al. (2019)	BRA	Latin America	2009	0.717	45	5.0	46.6	2.80 (1.77, 3.83)	1.11
Batliner et al. (2016)	USA	North America	2015	0.924	50	3.5	46.0	5.70 (4.37, 7.03)	0.87
Butten et al. (2019)	AUS	Oceania and Asia	2014	0.933	111	2.5	48.9	3.00 (2.68, 3.32)	1.79
Chang (1971)	CHN	Oceania and Asia	1968	0.482	90	7.0	48.8	3.86 (3.12, 4.60)	1.39
Chinnakotla et al. (2023)	ECU	Latin America	2012	0.736	458	3.0	56.4	6.70 (6.20, 7.20)	1.63
Dogar et al. (2011)	AUS	Oceania and Asia	2010	0.924	79	3.0	46.0	3.40 (2.58, 4.22)	1.32
Gonçalves et al. (2015)	BRA	Latin America	2011	0.727	205	6.5	51.8	3.11 (2.71, 3.51)	1.73
Gowda et al. (2009)	NZL	Oceania and Asia	2008	0.924	236	5.5	50.4	4.60 (4.12, 5.08)	1.65
Grim et al. (1994)	USA	North America	1989	0.875	457	10.5	48.0	4.52 (4.13, 4.91)	1.74
Guisilini et al. (2021)	BRA	Latin America	2012	0.732	402	2.8	51.7	2.60 (2.25, 2.95)	1.76
Ha (2014)	AUS	Oceania and Asia	2010	0.924	3985	7.5	NR	3.84 (3.62, 4.06)	1.85
Ha et al. (2014)	AUS	Oceania and Asia	2009	0.924	2918	7.5	NR	3.42 (3.33, 3.51)	1.90
Ha et al. (2016)	AUS	Oceania and Asia	2001	0.896	6918	7.5	NR	1.97 (1.94, 2.00)	1.91
Ha et al. (2016)	AUS	Oceania and Asia	2008	0.924	3251	7.5	NR	2.82 (2.81, 2.83)	1.91
Hagens et al. (2023)	PAN	Latin America	2022	0.820	106	9.0	NR	3.80 (3.25, 4.35)	1.58
Hallett et al. (2002)	AUS	Oceania and Asia	2000	0.869	48	5.0	48.0	2.44 (1.27, 3.61)	0.99
Hirooka et al. (2014)	BRA	Latin America	2007	0.722	246	4.0	NR	4.70 (4.28, 5.12)	1.71
Hopcraft et al. (2007)	AUS	Oceania and Asia	2003	0.869	393	8.0	49.0	4.43 (4.04, 4.82)	1.74
Jamieson et al. (2007)	AUS	Oceania and Asia	1989	0.864	196	6.0	50.5	2.85 (2.61, 3.09)	1.84
Jamieson et al. (2007)	AUS	Oceania and Asia	1990	0.864	463	6.0	51.3	2.85 (2.71, 2.99)	1.89
Jamieson et al. (2007)	AUS	Oceania and Asia	1991	0.864	550	6.0	49.7	2.72 (2.58, 2.86)	1.89
Jamieson et al. (2007)	AUS	Oceania and Asia	1992	0.864	465	6.0	50.3	3.09 (2.95, 3.23)	1.89
Jamieson et al. (2007)	AUS	Oceania and Asia	1993	0.864	543	6.0	48.6	2.64 (2.50, 2.78)	1.89
Jamieson et al. (2007)	AUS	Oceania and Asia	1994	0.864	450	6.0	48.3	3.09 (2.93, 3.25)	1.88
Jamieson et al. (2007)	AUS	Oceania and Asia	1995	0.864	509	6.0	49.3	3.20 (3.05, 3.35)	1.88
Jamieson et al. (2007)	AUS	Oceania and Asia	1996	0.896	574	6.0	51.1	2.79 (2.66, 2.92)	1.89
Jamieson et al. (2007)	AUS	Oceania and Asia	1997	0.896	467	6.0	49.1	3.09 (2.93, 3.25)	1.88
Jamieson et al. (2007)	AUS	Oceania and Asia	1998	0.896	463	6.0	49.4	3.09 (2.92, 3.26)	1.88
Jamieson et al. (2007)	AUS	Oceania and Asia	1999	0.896	494	6.0	50.6	3.37 (3.20, 3.54)	1.88
Jamieson et al. (2007)	AUS	Oceania and Asia	2000	0.869	547	6.0	49.8	3.94 (3.78, 4.10)	1.88
Jamieson et al. (2010)	AUS	Oceania and Asia	1995	0.864	145	7.0	NR	3.40 (2.80, 4.00)	1.54
Jones et al. (1992)	USA	North America	1991	0.875	381	4.0	49.5	4.88 (4.45, 5.31)	1.70
Kailis (1979)	AUS	Oceania and Asia	1971	0.864	201	7.5	52.2	6.33 (5.77, 6.89)	1.58
Kroon et al. (2019)	AUS	Oceania and Asia	2004	0.869	393	8.0	51.5	4.50 (4.05, 4.95)	1.68
Kroon et al. (2019)	AUS	Oceania and Asia	2012	0.924	263	8.0	51.5	2.80 (2.41, 3.19)	1.74
Kroon et al. (2019)	AUS	Oceania and Asia	2015	0.933	284	8.0	51.5	3.50 (3.12, 3.88)	1.74
Kroon et al. (2019)	AUS	Oceania and Asia	2016	0.933	87	8.0	51.5	2.70 (2.07, 3.33)	1.51
Kroon et al. (2019)	AUS	Oceania and Asia	2017	0.933	63	8.0	51.5	3.00 (2.28, 3.72)	1.42
Kruger et al. (2005)	AUS	Oceania and Asia	2004	0.869	31	3.5	50.0	4.29 (2.82, 5.76)	0.77
Kumar et al. (2013)	IND	Oceania and Asia	2012	0.572	743	10.0	51.3	2.79 (2.53, 3.05)	1.83
Lawrence (2009)	CAN	North America	2002	0.890	65	4.0	43.0	6.50 (5.15, 7.85)	0.85
Lawrence et al. (2009)	CAN	North America	2003	0.890	76	4.0	46.0	6.70 (5.50, 7.90)	0.96
Lawrence et al. (2009)	CAN	North America	2004	0.890	63	4.0	49.2	5.90 (4.70, 7.10)	0.96
Lee et al. (2022)	CAN	North America	2018	0.928	146	3.0	50.7	4.90 (4.04, 5.76)	1.27
Lemos et al. (2018)	BRA	Latin America	2007	0.708	100	5.0	52.0	6.43 (5.70, 7.16)	1.40
Lemos et al. (2018)	BRA	Latin America	2013	0.750	86	5.0	61.0	5.85 (5.00, 6.70)	1.28
Miranda et al. (2018)	BRA	Latin America	2010	0.722	52	5.0	52.9	4.02 (2.93, 5.11)	1.06
Oliveira et al. (2024)	BRA	Latin America	2023	0.760	125	3.6	37.6	2.16 (1.73, 2.59)	1.70
Peressini et al. (2004)	CAN	North America	2000	0.890	37	7.0	56.7	5.40 (4.40, 6.40)	1.14
Peressini et al. (2004)	CAN	North America	2000	0.890	87	4.0	54.0	4.20 (3.34, 5.06)	1.27
Phipps et al. (2012)	USA	North America	2010	0.916	8323	3.0	52.0	3.50 (3.07, 3.93)	1.70
Rigonatto et al. (2001)	BRA	Latin America	1991	0.620	171	6.5	54.5	5.52 (4.94, 6.10)	1.56
Sampaio et al. (2010)	BRA	Latin America	2003	0.682	288	3.2	NR	4.18 (3.71, 4.65)	1.67
Schroth et al. (2005)	CAN	North America	2001	0.890	236	3.0	55.1	4.50 (3.86, 5.14)	1.49
Seow et al. (1996)	AUS	Oceania and Asia	1995	0.864	184	4.8	52.2	3.80 (3.27, 4.33)	1.60
Singh et al. (2011)	IND	Oceania and Asia	2010	0.572	418	5.0	46.0	4.13 (3.76, 4.50)	1.75
Smith et al. (2015)	AUS	Oceania and Asia	2014	0.933	173	3.5	45.7	2.40 (1.79, 3.01)	1.53
Tsai et al. (2021)	CAN	North America	2013	0.927	344	2.0	NR	7.00 (6.35, 7.65)	1.49
Zeng et al. (2005)	CHN	Oceania and Asia	2004	0.586	470	4.0	NR	3.36 (3.00, 3.72)	1.76
Overall, DL (I^2 = 98.7%, $p < 0.001$)								3.75 (3.58, 3.92)	100.00

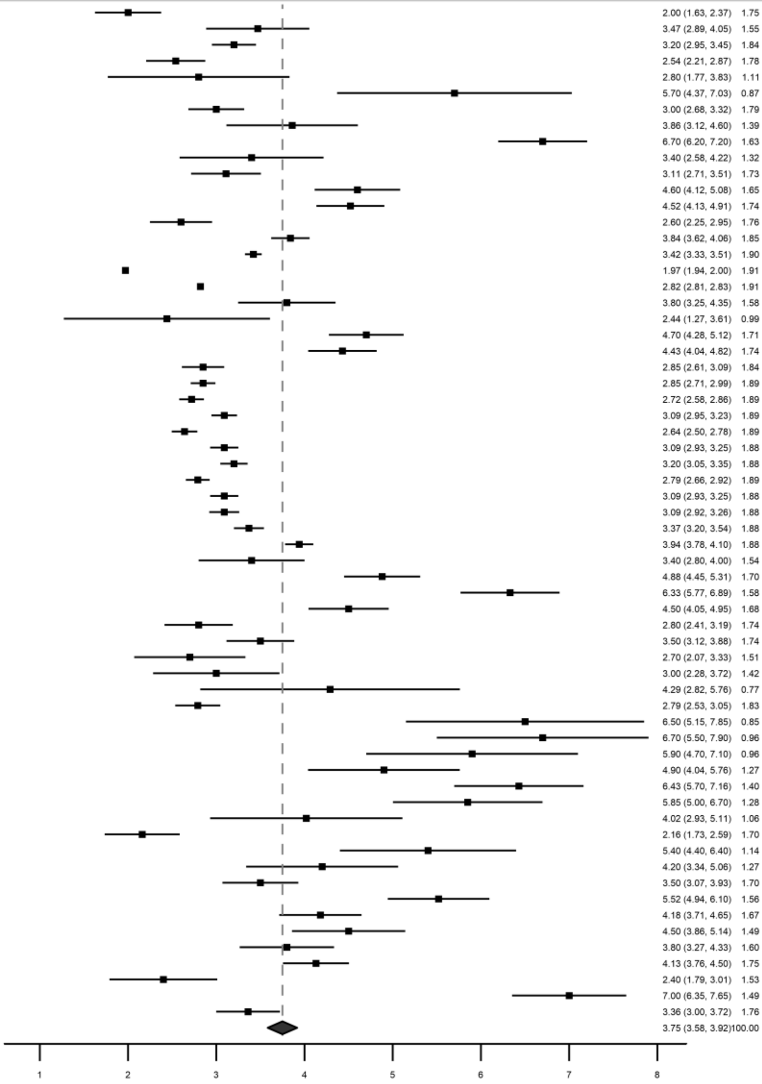

Figure S2. Forest plot of the mean dmft index (deciduous dentition) among Indigenous children by age group.

Subgroup and Authors	Country	Global region	Year of data colletion	HDI	Sample size	Mean age	% Female	Effect (95% CI)	% Weight
2-4.9 years									
Batliner et al. (2016)	USA	North America	2015	0.924	50	3.5	46.0	5.70 (4.37, 7.03)	0.87
Butten et al. (2019)	AUS	Oceania and Asia	2014	0.933	111	2.5	48.9	3.00 (2.68, 3.32)	1.79
Chinnakotla et al. (2023)	ECU	Latin America	2012	0.736	458	3.0	56.4	6.70 (6.20, 7.20)	1.63
Dogar et al. (2011)	AUS	Oceania and Asia	2010	0.924	79	3.0	46.0	3.40 (2.58, 4.22)	1.32
Guisilini et al. (2021)	BRA	Latin America	2012	0.732	402	2.8	51.7	2.60 (2.25, 2.95)	1.76
Hirooka et al. (2014)	BRA	Latin America	2007	0.722	246	4.0	NR	4.70 (4.28, 5.12)	1.71
Jones et al. (1992)	USA	North America	1991	0.875	381	4.0	49.5	4.88 (4.45, 5.31)	1.70
Kruger et al. (2005)	AUS	Oceania and Asia	2004	0.869	31	3.5	50.0	4.29 (2.82, 5.76)	0.77
Lawrence (2009)	CAN	North America	2002	0.890	65	4.0	43.0	6.50 (5.15, 7.85)	0.85
Lawrence et al. (2009)	CAN	North America	2003	0.890	76	4.0	46.0	6.70 (5.50, 7.90)	0.96
Lawrence et al. (2009)	CAN	North America	2004	0.890	63	4.0	49.2	5.90 (4.70, 7.10)	0.96
Lee et al. (2022)	CAN	North America	2018	0.928	146	3.0	50.7	4.90 (4.04, 5.76)	1.27
Oliveira et al. (2024)	BRA	Latin America	2023	0.760	125	3.6	37.6	2.16 (1.73, 2.59)	1.70
Peressini et al. (2004)	CAN	North America	2000	0.890	87	4.0	54.0	4.20 (3.34, 5.06)	1.27
Phipps et al. (2012)	USA	North America	2010	0.916	8323	3.0	52.0	3.50 (3.07, 3.93)	1.70
Sampaio et al. (2010)	BRA	Latin America	2003	0.682	288	3.2	NR	4.18 (3.71, 4.65)	1.67
Schroth et al. (2005)	CAN	North America	2001	0.890	236	3.0	55.1	4.50 (3.86, 5.14)	1.49
Seow et al. (1996)	AUS	Oceania and Asia	1995	0.864	184	4.8	52.2	3.80 (3.27, 4.33)	1.60
Smith et al. (2015)	AUS	Oceania and Asia	2014	0.933	173	3.5	45.7	2.40 (1.79, 3.01)	1.53
Tsai et al. (2021)	CAN	North America	2013	0.927	344	2.0	NR	7.00 (6.35, 7.65)	1.49
Zeng et al. (2005)	CHN	Oceania and Asia	2004	0.586	470	4.0	NR	3.36 (3.00, 3.72)	1.76
Subgroup, DL (I^2 = 95.9%, p < 0.001)								4.44 (3.84, 5.03)	29.81
5-6.9 years									
Alves Filho et al. (2009)	BRA	Latin America	2006	0.722	246	6.8	48.6	2.00 (1.63, 2.37)	1.75
Arantes et al. (2021)	BRA	Latin America	2013	0.750	606	5.0	NR	3.20 (2.95, 3.45)	1.84
Baldisserotto et al. (2019)	BRA	Latin America	2009	0.717	45	5.0	46.6	2.80 (1.77, 3.83)	1.11
Gonçalves et al. (2015)	BRA	Latin America	2011	0.727	205	6.5	51.8	3.11 (2.71, 3.51)	1.73
Gowda et al. (2009)	NZL	Oceania and Asia	2008	0.924	236	5.5	50.4	4.60 (4.12, 5.08)	1.65
Hallett et al. (2002)	AUS	Oceania and Asia	2000	0.869	48	5.0	48.0	2.44 (1.27, 3.61)	0.99
Jamieson et al. (2007)	AUS	Oceania and Asia	1989	0.864	196	6.0	50.5	2.85 (2.61, 3.09)	1.84
Jamieson et al. (2007)	AUS	Oceania and Asia	1990	0.864	463	6.0	51.3	2.85 (2.71, 2.99)	1.89
Jamieson et al. (2007)	AUS	Oceania and Asia	1991	0.864	550	6.0	49.7	2.72 (2.58, 2.86)	1.89
Jamieson et al. (2007)	AUS	Oceania and Asia	1992	0.864	465	6.0	50.3	3.09 (2.95, 3.23)	1.89
Jamieson et al. (2007)	AUS	Oceania and Asia	1993	0.864	543	6.0	48.6	2.64 (2.50, 2.78)	1.89
Jamieson et al. (2007)	AUS	Oceania and Asia	1994	0.864	450	6.0	48.3	3.09 (2.93, 3.25)	1.88
Jamieson et al. (2007)	AUS	Oceania and Asia	1995	0.864	509	6.0	49.3	3.20 (3.05, 3.35)	1.88
Jamieson et al. (2007)	AUS	Oceania and Asia	1996	0.896	574	6.0	51.1	2.79 (2.66, 2.92)	1.89
Jamieson et al. (2007)	AUS	Oceania and Asia	1997	0.896	467	6.0	49.1	3.09 (2.93, 3.25)	1.88
Jamieson et al. (2007)	AUS	Oceania and Asia	1998	0.896	463	6.0	49.4	3.09 (2.92, 3.26)	1.88
Jamieson et al. (2007)	AUS	Oceania and Asia	1999	0.896	494	6.0	50.6	3.37 (3.20, 3.54)	1.88
Jamieson et al. (2007)	AUS	Oceania and Asia	2000	0.869	547	6.0	49.8	3.94 (3.78, 4.10)	1.88
Lemos et al. (2018)	BRA	Latin America	2007	0.708	100	5.0	52.0	6.43 (5.70, 7.16)	1.40
Lemos et al. (2018)	BRA	Latin America	2013	0.750	86	5.0	61.0	5.85 (5.00, 6.70)	1.28
Miranda et al. (2018)	BRA	Latin America	2010	0.722	52	5.0	52.9	4.02 (2.93, 5.11)	1.06
Rigonatto et al. (2001)	BRA	Latin America	1991	0.620	171	6.5	54.5	5.52 (4.94, 6.10)	1.56
Singh et al. (2011)	IND	Oceania and Asia	2010	0.572	418	5.0	46.0	4.13 (3.76, 4.50)	1.75
Subgroup, DL (I^2 = 95.7%, p < 0.001)								3.43 (3.20, 3.65)	38.68
7-11 years									
Arantes et al. (2001)	BRA	Latin America	1997	0.668	105	7.0	53.0	3.47 (2.89, 4.05)	1.55
Arrow (2016)	AUS	Oceania and Asia	2014	0.924	268	7.5	47.6	2.54 (2.21, 2.87)	1.78
Chang (1971)	CHN	Oceania and Asia	1968	0.482	90	7.0	48.8	3.86 (3.12, 4.60)	1.39
Grim et al. (1994)	USA	North America	1989	0.875	457	10.5	48.0	4.52 (4.13, 4.91)	1.74
Ha (2014)	AUS	Oceania and Asia	2010	0.924	3985	7.5	NR	3.84 (3.62, 4.06)	1.85
Ha et al. (2014)	AUS	Oceania and Asia	2009	0.924	2918	7.5	NR	3.42 (3.33, 3.51)	1.90
Ha et al. (2016)	AUS	Oceania and Asia	2001	0.896	6918	7.5	NR	1.97 (1.94, 2.00)	1.91
Ha et al. (2016)	AUS	Oceania and Asia	2008	0.924	3251	7.5	NR	2.82 (2.81, 2.83)	1.91
Hagens et al. (2023)	PAN	Latin America	2022	0.820	106	9.0	NR	3.80 (3.25, 4.35)	1.58
Hopcraft et al. (2007)	AUS	Oceania and Asia	2003	0.869	393	8.0	49.0	4.43 (4.04, 4.82)	1.74
Jamieson et al. (2010)	AUS	Oceania and Asia	1995	0.864	145	7.0	NR	3.40 (2.80, 4.00)	1.54
Kailis (1979)	AUS	Oceania and Asia	1971	0.864	201	7.5	52.2	6.33 (5.77, 6.89)	1.58
Kroon et al. (2019)	AUS	Oceania and Asia	2004	0.869	393	8.0	51.5	4.50 (4.05, 4.95)	1.68
Kroon et al. (2019)	AUS	Oceania and Asia	2012	0.924	263	8.0	51.5	2.80 (2.41, 3.19)	1.74
Kroon et al. (2019)	AUS	Oceania and Asia	2015	0.933	284	8.0	51.5	3.50 (3.12, 3.88)	1.74
Kroon et al. (2019)	AUS	Oceania and Asia	2016	0.933	87	8.0	51.5	2.70 (2.07, 3.33)	1.51
Kroon et al. (2019)	AUS	Oceania and Asia	2017	0.933	63	8.0	51.5	3.00 (2.28, 3.72)	1.42
Kumar et al. (2013)	IND	Oceania and Asia	2012	0.572	743	10.0	51.3	2.79 (2.53, 3.05)	1.83
Peressini et al. (2004)	CAN	North America	2000	0.890	37	7.0	56.7	5.40 (4.40, 6.40)	1.14
Subgroup, DL (I^2 = 99.4%, p < 0.001)								3.58 (3.28, 3.88)	31.52
Heterogeneity between groups: p = 0.008									
Overall, DL (I^2 = 98.7%, p < 0.001)								3.75 (3.58, 3.92)	100.00

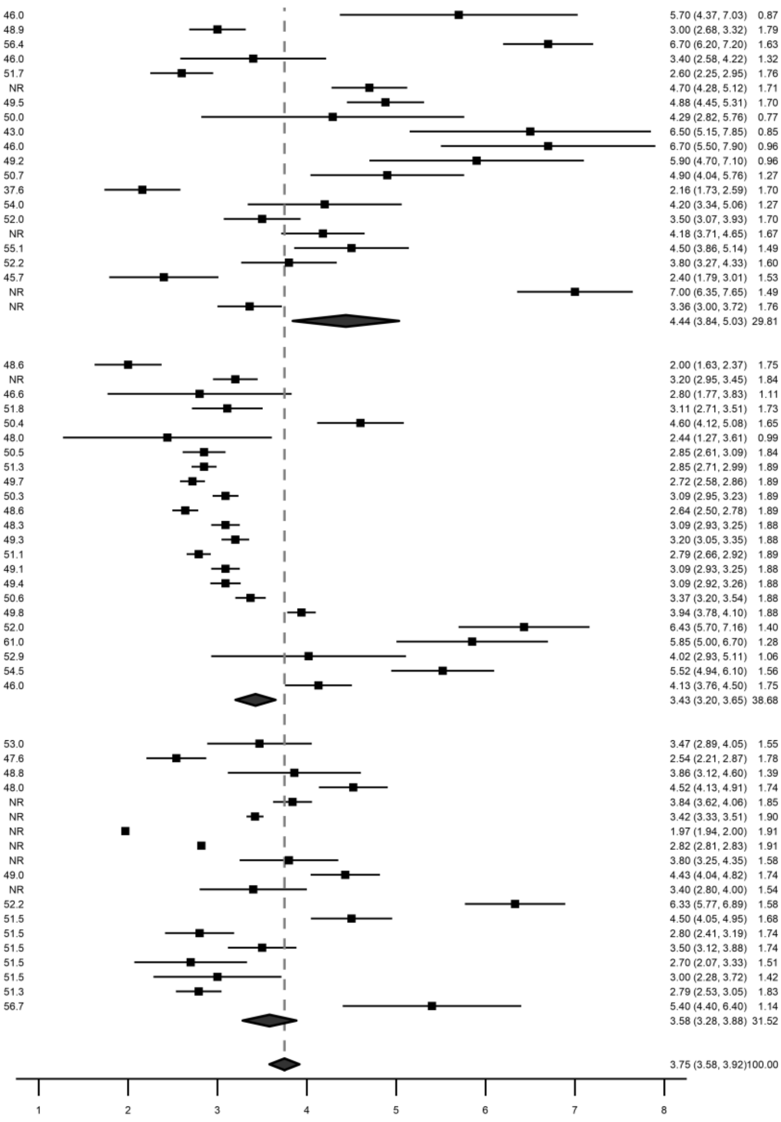

Figure S3. Forest plot of the mean dmft index (deciduous dentition) among Indigenous children by the percentage of female individuals.

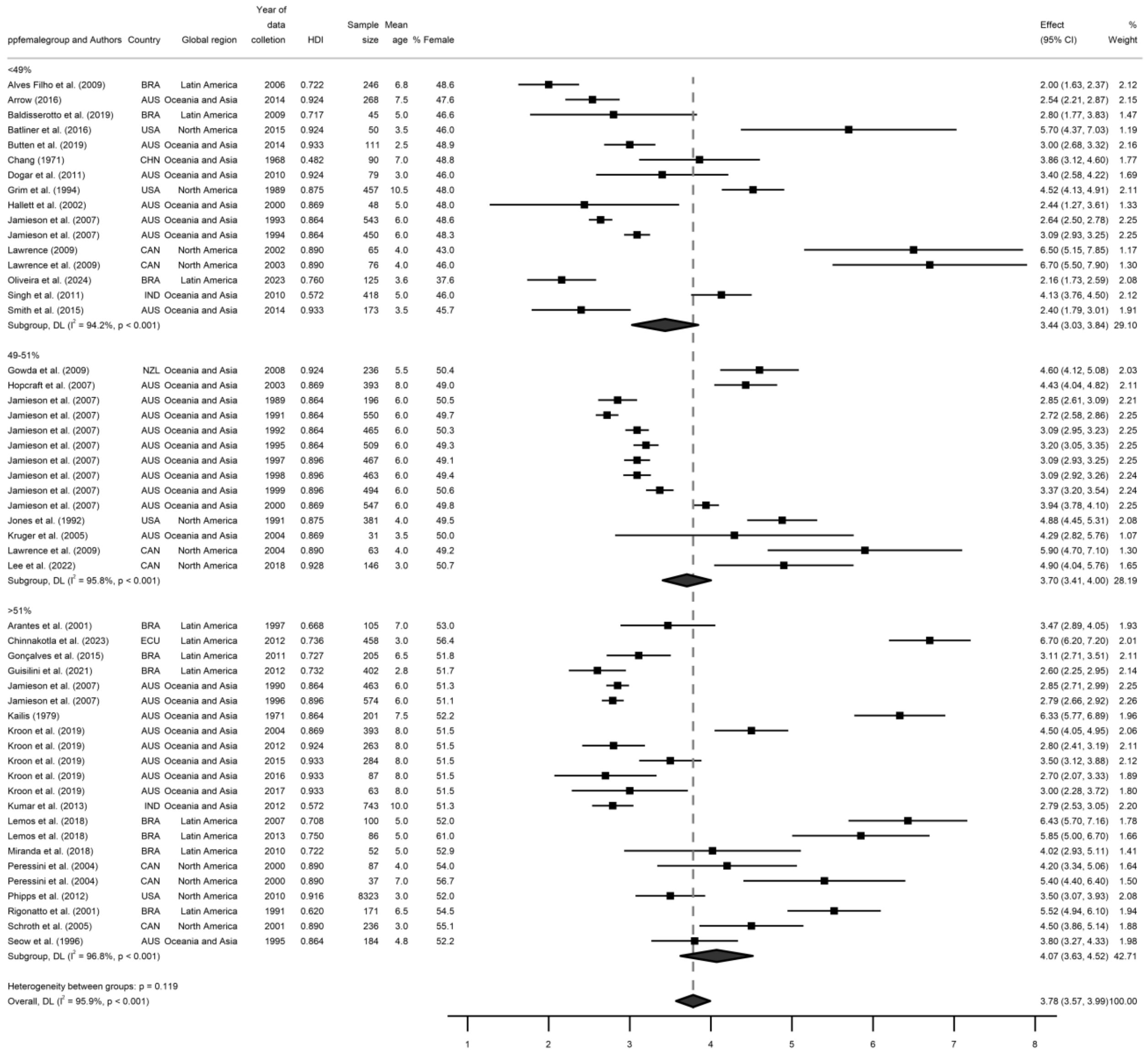

Figure S4. Forest plot of the mean dmft index (deciduous dentition) among Indigenous children by country.

Subgroup Country and Authors	Country	Global region	Year of data colletion	HDI	Sample size	Mean age	% Female	Effect (95% CI)	% Weight
Australia									
Arrow (2016)	AUS	Oceania and Asia	2014	0.924	268	7.5	47.6	2.54 (2.21, 2.87)	1.78
Butten et al. (2019)	AUS	Oceania and Asia	2014	0.933	111	2.5	48.9	3.00 (2.68, 3.32)	1.79
Dogar et al. (2011)	AUS	Oceania and Asia	2010	0.924	79	3.0	46.0	3.40 (2.58, 4.22)	1.32
Ha (2014)	AUS	Oceania and Asia	2010	0.924	3985	7.5	NR	3.84 (3.62, 4.06)	1.85
Ha et al. (2014)	AUS	Oceania and Asia	2009	0.924	2918	7.5	NR	3.42 (3.33, 3.51)	1.90
Ha et al. (2016)	AUS	Oceania and Asia	2001	0.896	6918	7.5	NR	1.97 (1.94, 2.00)	1.91
Ha et al. (2016)	AUS	Oceania and Asia	2008	0.924	3251	7.5	NR	2.82 (2.81, 2.83)	1.91
Hallett et al. (2002)	AUS	Oceania and Asia	2000	0.869	48	5.0	48.0	2.44 (1.27, 3.61)	0.99
Hopcraft et al. (2007)	AUS	Oceania and Asia	2003	0.869	393	8.0	49.0	4.43 (4.04, 4.82)	1.74
Jamieson et al. (2007)	AUS	Oceania and Asia	1989	0.864	196	6.0	50.5	2.85 (2.61, 3.09)	1.84
Jamieson et al. (2007)	AUS	Oceania and Asia	1990	0.864	463	6.0	51.3	2.85 (2.71, 2.99)	1.89
Jamieson et al. (2007)	AUS	Oceania and Asia	1991	0.864	550	6.0	49.7	2.72 (2.58, 2.86)	1.89
Jamieson et al. (2007)	AUS	Oceania and Asia	1992	0.864	465	6.0	50.3	3.09 (2.95, 3.23)	1.89
Jamieson et al. (2007)	AUS	Oceania and Asia	1993	0.864	543	6.0	48.6	2.64 (2.50, 2.78)	1.89
Jamieson et al. (2007)	AUS	Oceania and Asia	1994	0.864	450	6.0	48.3	3.09 (2.93, 3.25)	1.88
Jamieson et al. (2007)	AUS	Oceania and Asia	1995	0.864	509	6.0	49.3	3.20 (3.05, 3.35)	1.88
Jamieson et al. (2007)	AUS	Oceania and Asia	1996	0.896	574	6.0	51.1	2.79 (2.66, 2.92)	1.89
Jamieson et al. (2007)	AUS	Oceania and Asia	1997	0.896	467	6.0	49.1	3.09 (2.93, 3.25)	1.88
Jamieson et al. (2007)	AUS	Oceania and Asia	1998	0.896	463	6.0	49.4	3.09 (2.92, 3.26)	1.88
Jamieson et al. (2007)	AUS	Oceania and Asia	1999	0.896	494	6.0	50.6	3.37 (3.20, 3.54)	1.88
Jamieson et al. (2007)	AUS	Oceania and Asia	2000	0.869	547	6.0	49.8	3.94 (3.78, 4.10)	1.88
Jamieson et al. (2010)	AUS	Oceania and Asia	1995	0.864	145	7.0	NR	3.40 (2.80, 4.00)	1.54
Kailis (1979)	AUS	Oceania and Asia	1971	0.864	201	7.5	52.2	6.33 (5.77, 6.89)	1.58
Kroon et al. (2019)	AUS	Oceania and Asia	2004	0.869	393	8.0	51.5	4.50 (4.05, 4.95)	1.68
Kroon et al. (2019)	AUS	Oceania and Asia	2012	0.924	263	8.0	51.5	2.80 (2.41, 3.19)	1.74
Kroon et al. (2019)	AUS	Oceania and Asia	2015	0.933	284	8.0	51.5	3.50 (3.12, 3.88)	1.74
Kroon et al. (2019)	AUS	Oceania and Asia	2016	0.933	87	8.0	51.5	2.70 (2.07, 3.33)	1.51
Kroon et al. (2019)	AUS	Oceania and Asia	2017	0.933	63	8.0	51.5	3.00 (2.28, 3.72)	1.42
Kruger et al. (2005)	AUS	Oceania and Asia	2004	0.869	31	3.5	50.0	4.29 (2.82, 5.76)	0.77
Seow et al. (1996)	AUS	Oceania and Asia	1995	0.864	184	4.8	52.2	3.80 (3.27, 4.33)	1.60
Smith et al. (2015)	AUS	Oceania and Asia	2014	0.933	173	3.5	45.7	2.40 (1.79, 3.01)	1.53
Subgroup, DL (I^2 = 99.1%, p < 0.001)								3.24 (3.03, 3.44)	52.85
Brazil									
Alves Filho et al. (2009)	BRA	Latin America	2006	0.722	246	6.8	48.6	2.00 (1.63, 2.37)	1.75
Arantes et al. (2001)	BRA	Latin America	1997	0.668	105	7.0	53.0	3.47 (2.89, 4.05)	1.55
Arantes et al. (2021)	BRA	Latin America	2013	0.750	606	5.0	NR	3.20 (2.95, 3.45)	1.84
Baldisserotto et al. (2019)	BRA	Latin America	2009	0.717	45	5.0	46.6	2.80 (1.77, 3.83)	1.11
Gonçalves et al. (2015)	BRA	Latin America	2011	0.727	205	6.5	51.8	3.11 (2.71, 3.51)	1.73
Guisilini et al. (2021)	BRA	Latin America	2012	0.732	402	2.8	51.7	2.60 (2.25, 2.95)	1.76
Hirooka et al. (2014)	BRA	Latin America	2007	0.722	246	4.0	NR	4.70 (4.28, 5.12)	1.71
Lemos et al. (2018)	BRA	Latin America	2007	0.708	100	5.0	52.0	6.43 (5.70, 7.16)	1.40
Lemos et al. (2018)	BRA	Latin America	2013	0.750	86	5.0	61.0	5.85 (5.00, 6.70)	1.28
Miranda et al. (2018)	BRA	Latin America	2010	0.722	52	5.0	52.9	4.02 (2.93, 5.11)	1.06
Oliveira et al. (2024)	BRA	Latin America	2023	0.760	125	3.6	37.6	2.16 (1.73, 2.59)	1.70
Rigonatto et al. (2001)	BRA	Latin America	1991	0.620	171	6.5	54.5	5.52 (4.94, 6.10)	1.56
Sampaio et al. (2010)	BRA	Latin America	2003	0.682	288	3.2	NR	4.18 (3.71, 4.65)	1.67
Subgroup, DL (I^2 = 96.1%, p < 0.001)								3.83 (3.16, 4.49)	20.11
Canada									
Lawrence (2009)	CAN	North America	2002	0.890	65	4.0	43.0	6.50 (5.15, 7.85)	0.85
Lawrence et al. (2009)	CAN	North America	2003	0.890	76	4.0	46.0	6.70 (5.50, 7.90)	0.96
Lawrence et al. (2009)	CAN	North America	2004	0.890	63	4.0	49.2	5.90 (4.70, 7.10)	0.96
Lee et al. (2022)	CAN	North America	2018	0.928	146	3.0	50.7	4.90 (4.04, 5.76)	1.27
Peressini et al. (2004)	CAN	North America	2000	0.890	37	7.0	56.7	5.40 (4.40, 6.40)	1.14
Peressini et al. (2004)	CAN	North America	2000	0.890	87	4.0	54.0	4.20 (3.34, 5.06)	1.27
Schroth et al. (2005)	CAN	North America	2001	0.890	236	3.0	55.1	4.50 (3.86, 5.14)	1.49
Tsai et al. (2021)	CAN	North America	2013	0.927	344	2.0	NR	7.00 (6.35, 7.65)	1.49
Subgroup, DL (I^2 = 85.1%, p < 0.001)								5.60 (4.77, 6.43)	9.44
China									
Chang (1971)	CHN	Oceania and Asia	1968	0.482	90	7.0	48.8	3.86 (3.12, 4.60)	1.39
Zeng et al. (2005)	CHN	Oceania and Asia	2004	0.586	470	4.0	NR	3.36 (3.00, 3.72)	1.76
Subgroup, DL (I^2 = 28.7%, p = 0.236)								3.50 (3.06, 3.94)	3.15
India									
Kumar et al. (2013)	IND	Oceania and Asia	2012	0.572	743	10.0	51.3	2.79 (2.53, 3.05)	1.83
Singh et al. (2011)	IND	Oceania and Asia	2010	0.572	418	5.0	46.0	4.13 (3.76, 4.50)	1.75
Subgroup, DL (I^2 = 97.0%, p < 0.001)								3.45 (2.14, 4.77)	3.58
Others									
Chinnakotla et al. (2023)	ECU	Latin America	2012	0.736	458	3.0	56.4	6.70 (6.20, 7.20)	1.63
Gowda et al. (2009)	NZL	Oceania and Asia	2008	0.924	236	5.5	50.4	4.60 (4.12, 5.08)	1.65
Hagens et al. (2023)	PAN	Latin America	2022	0.820	106	9.0	NR	3.80 (3.25, 4.35)	1.58
Subgroup, DL (I^2 = 96.9%, p < 0.001)								5.04 (3.36, 6.71)	4.87
USA									
Batliner et al. (2016)	USA	North America	2015	0.924	50	3.5	46.0	5.70 (4.37, 7.03)	0.87
Grim et al. (1994)	USA	North America	1989	0.875	457	10.5	48.0	4.52 (4.13, 4.91)	1.74
Jones et al. (1992)	USA	North America	1991	0.875	381	4.0	49.5	4.88 (4.45, 5.31)	1.70
Phipps et al. (2012)	USA	North America	2010	0.916	8323	3.0	52.0	3.50 (3.07, 3.93)	1.70
Subgroup, DL (I^2 = 88.2%, p < 0.001)								4.52 (3.77, 5.27)	6.00
Heterogeneity between groups: p = 0.000									
Overall, DL (I^2 = 98.7%, p < 0.001)								3.75 (3.58, 3.92)	100.00

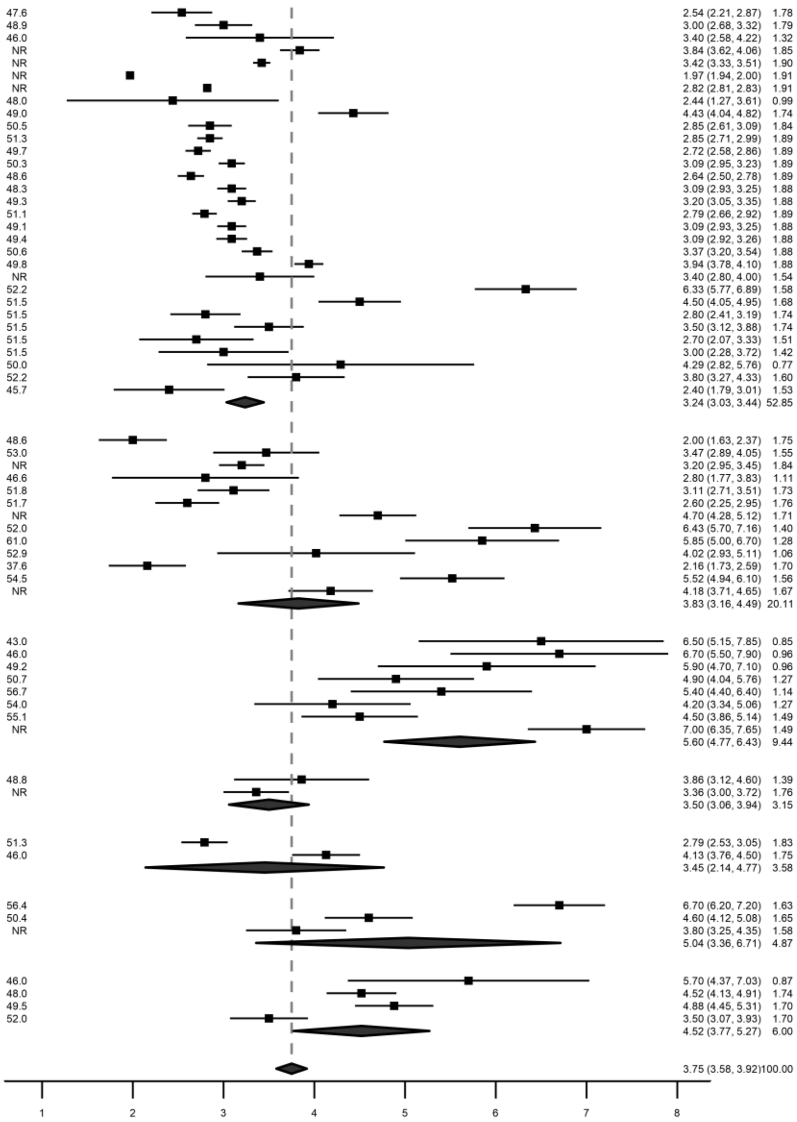

Figure S5. Forest plot of the mean dmft index (deciduous dentition) among Indigenous children by global region.

Global region and Authors	Country	Year of data colletion	HDI	Sample size	Mean age	% Female	Effect (95% CI)	% Weight
**North America**								
Batliner et al. (2016)	USA	2015	0.924	50	3.5	46.0	5.70 (4.37, 7.03)	0.87
Grim et al. (1994)	USA	1989	0.875	457	10.5	48.0	4.52 (4.13, 4.91)	1.74
Jones et al. (1992)	USA	1991	0.875	381	4.0	49.5	4.88 (4.45, 5.31)	1.70
Lawrence (2009)	CAN	2002	0.890	65	4.0	43.0	6.50 (5.15, 7.85)	0.85
Lawrence et al. (2009)	CAN	2003	0.890	76	4.0	46.0	6.70 (5.50, 7.90)	0.96
Lawrence et al. (2009)	CAN	2004	0.890	63	4.0	49.2	5.90 (4.70, 7.10)	0.96
Lee et al. (2022)	CAN	2018	0.928	146	3.0	50.7	4.90 (4.04, 5.76)	1.27
Peressini et al. (2004)	CAN	2000	0.890	37	7.0	56.7	5.40 (4.40, 6.40)	1.14
Peressini et al. (2004)	CAN	2000	0.890	87	4.0	54.0	4.20 (3.34, 5.06)	1.27
Phipps et al. (2012)	USA	2010	0.916	8323	3.0	52.0	3.50 (3.07, 3.93)	1.70
Schroth et al. (2005)	CAN	2001	0.890	236	3.0	55.1	4.50 (3.86, 5.14)	1.49
Tsai et al. (2021)	CAN	2013	0.927	344	2.0	NR	7.00 (6.35, 7.65)	1.49
Subgroup, DL (I^2 = 89.7%, p < 0.001)							5.23 (4.60, 5.86)	15.45
**Latin America**								
Alves Filho et al. (2009)	BRA	2006	0.722	246	6.8	48.6	2.00 (1.63, 2.37)	1.75
Arantes et al. (2001)	BRA	1997	0.668	105	7.0	53.0	3.47 (2.89, 4.05)	1.55
Arantes et al. (2021)	BRA	2013	0.750	606	5.0	NR	3.20 (2.95, 3.45)	1.84
Baldisserotto et al. (2019)	BRA	2009	0.717	45	5.0	46.6	2.80 (1.77, 3.83)	1.11
Chinnakotla et al. (2023)	ECU	2012	0.736	458	3.0	56.4	6.70 (6.20, 7.20)	1.63
Gonçalves et al. (2015)	BRA	2011	0.727	205	6.5	51.8	3.11 (2.71, 3.51)	1.73
Guisilini et al. (2021)	BRA	2012	0.732	402	2.8	51.7	2.60 (2.25, 2.95)	1.76
Hagens et al. (2023)	PAN	2022	0.820	106	9.0	NR	3.80 (3.25, 4.35)	1.58
Hirooka et al. (2014)	BRA	2007	0.722	246	4.0	NR	4.70 (4.28, 5.12)	1.71
Lemos et al. (2018)	BRA	2007	0.708	100	5.0	52.0	6.43 (5.70, 7.16)	1.40
Lemos et al. (2018)	BRA	2013	0.750	86	5.0	61.0	5.85 (5.00, 6.70)	1.28
Miranda et al. (2018)	BRA	2010	0.722	52	5.0	52.9	4.02 (2.93, 5.11)	1.06
Oliveira et al. (2024)	BRA	2023	0.760	125	3.6	37.6	2.16 (1.73, 2.59)	1.70
Rigonatto et al. (2001)	BRA	1991	0.620	171	6.5	54.5	5.52 (4.94, 6.10)	1.56
Sampaio et al. (2010)	BRA	2003	0.682	288	3.2	NR	4.18 (3.71, 4.65)	1.67
Subgroup, DL (I^2 = 97.0%, p < 0.001)							4.02 (3.32, 4.73)	23.33
**Oceania and Asia**								
Arrow (2016)	AUS	2014	0.924	268	7.5	47.6	2.54 (2.21, 2.87)	1.78
Butten et al. (2019)	AUS	2014	0.933	111	2.5	48.9	3.00 (2.68, 3.32)	1.79
Chang (1971)	CHN	1968	0.482	90	7.0	48.8	3.86 (3.12, 4.60)	1.39
Dogar et al. (2011)	AUS	2010	0.924	79	3.0	46.0	3.40 (2.58, 4.22)	1.32
Gowda et al. (2009)	NZL	2008	0.924	236	5.5	50.4	4.60 (4.12, 5.08)	1.65
Ha (2014)	AUS	2010	0.924	3985	7.5	NR	3.84 (3.62, 4.06)	1.85
Ha et al. (2014)	AUS	2009	0.924	2918	7.5	NR	3.42 (3.33, 3.51)	1.90
Ha et al. (2016)	AUS	2001	0.896	6918	7.5	NR	1.97 (1.94, 2.00)	1.91
Ha et al. (2016)	AUS	2008	0.924	3251	7.5	NR	2.82 (2.81, 2.83)	1.91
Hallett et al. (2002)	AUS	2000	0.869	48	5.0	48.0	2.44 (1.27, 3.61)	0.99
Hopcraft et al. (2007)	AUS	2003	0.869	393	8.0	49.0	4.43 (4.04, 4.82)	1.74
Jamieson et al. (2007)	AUS	1989	0.864	196	6.0	50.5	2.85 (2.61, 3.09)	1.84
Jamieson et al. (2007)	AUS	1990	0.864	463	6.0	51.3	2.85 (2.71, 2.99)	1.89
Jamieson et al. (2007)	AUS	1991	0.864	550	6.0	49.7	2.72 (2.58, 2.86)	1.89
Jamieson et al. (2007)	AUS	1992	0.864	465	6.0	50.3	3.09 (2.95, 3.23)	1.89
Jamieson et al. (2007)	AUS	1993	0.864	543	6.0	48.6	2.64 (2.50, 2.78)	1.89
Jamieson et al. (2007)	AUS	1994	0.864	450	6.0	48.3	3.09 (2.93, 3.25)	1.88
Jamieson et al. (2007)	AUS	1995	0.864	509	6.0	49.3	3.20 (3.05, 3.35)	1.88
Jamieson et al. (2007)	AUS	1996	0.896	574	6.0	51.1	2.79 (2.66, 2.92)	1.89
Jamieson et al. (2007)	AUS	1997	0.896	467	6.0	49.1	3.09 (2.93, 3.25)	1.88
Jamieson et al. (2007)	AUS	1998	0.896	463	6.0	49.4	3.09 (2.92, 3.26)	1.88
Jamieson et al. (2007)	AUS	1999	0.896	494	6.0	50.6	3.37 (3.20, 3.54)	1.88
Jamieson et al. (2007)	AUS	2000	0.869	547	6.0	49.8	3.94 (3.78, 4.10)	1.88
Jamieson et al. (2010)	AUS	1995	0.864	145	7.0	NR	3.40 (2.80, 4.00)	1.54
Kailis (1979)	AUS	1971	0.864	201	7.5	52.2	6.33 (5.77, 6.89)	1.58
Kroon et al. (2019)	AUS	2004	0.869	393	8.0	51.5	4.50 (4.05, 4.95)	1.68
Kroon et al. (2019)	AUS	2012	0.924	263	8.0	51.5	2.80 (2.41, 3.19)	1.74
Kroon et al. (2019)	AUS	2015	0.933	284	8.0	51.5	3.50 (3.12, 3.88)	1.74
Kroon et al. (2019)	AUS	2016	0.933	87	8.0	51.5	2.70 (2.07, 3.33)	1.51
Kroon et al. (2019)	AUS	2017	0.933	63	8.0	51.5	3.00 (2.28, 3.72)	1.42
Kruger et al. (2005)	AUS	2004	0.869	31	3.5	50.0	4.29 (2.82, 5.76)	0.77
Kumar et al. (2013)	IND	2012	0.572	743	10.0	51.3	2.79 (2.53, 3.05)	1.83
Seow et al. (1996)	AUS	1995	0.864	184	4.8	52.2	3.80 (3.27, 4.33)	1.60
Singh et al. (2011)	IND	2010	0.572	418	5.0	46.0	4.13 (3.76, 4.50)	1.75
Smith et al. (2015)	AUS	2014	0.933	173	3.5	45.7	2.40 (1.79, 3.01)	1.53
Zeng et al. (2005)	CHN	2004	0.586	470	4.0	NR	3.36 (3.00, 3.72)	1.76
Subgroup, DL (I^2 = 99.0%, p < 0.001)							3.30 (3.11, 3.50)	61.22
Heterogeneity between groups: p = 0.000								
Overall, DL (I^2 = 98.7%, p < 0.001)							3.75 (3.58, 3.92)	100.00

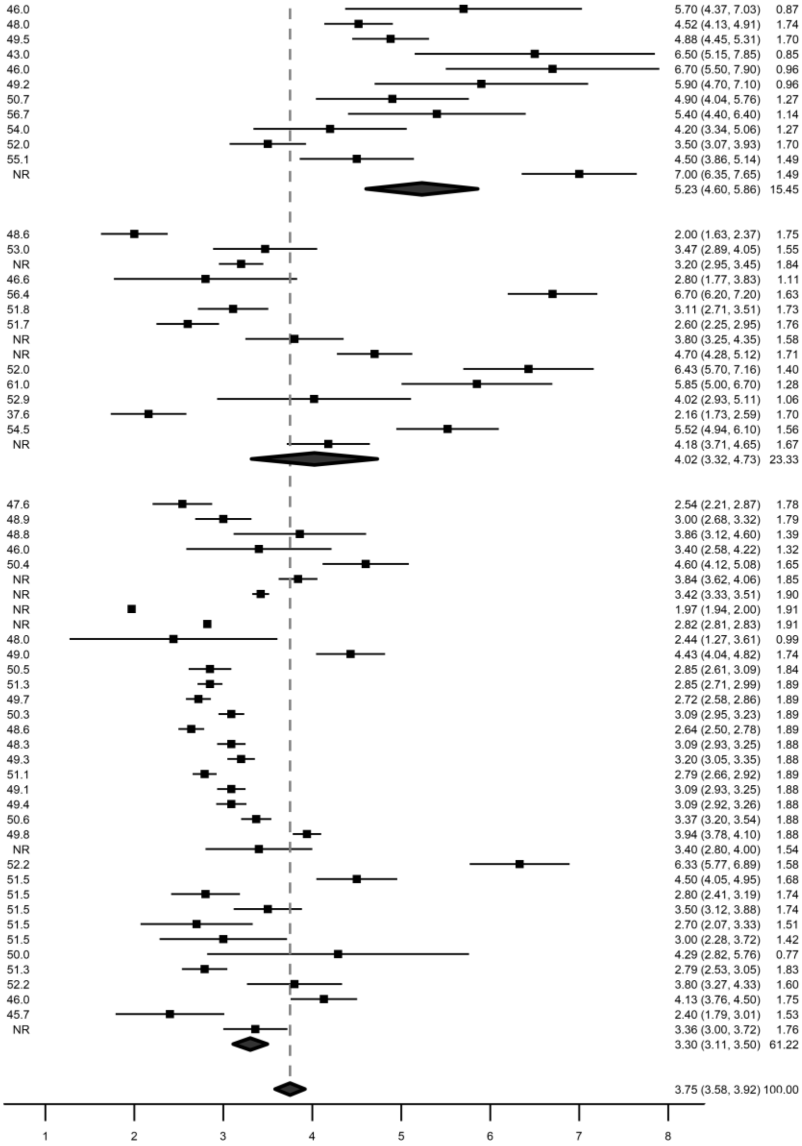

Figure S6. Forest plot of the mean dmft index (deciduous dentition) among Indigenous children by HDI.

Subgroup and Authors	Country	Global region	Year of data colletion	HDI	Sample size	Mean age	% Female	Effect (95% CI)	% Weight
<= 0.82									
Alves Filho et al. (2009)	BRA	Latin America	2006	0.722	246	6.8	48.6	2.00 (1.63, 2.37)	1.75
Arantes et al. (2001)	BRA	Latin America	1997	0.668	105	7.0	53.0	3.47 (2.89, 4.05)	1.55
Arantes et al. (2021)	BRA	Latin America	2013	0.750	606	5.0	NR	3.20 (2.95, 3.45)	1.84
Baldisserotto et al. (2019)	BRA	Latin America	2009	0.717	45	5.0	46.6	2.80 (1.77, 3.83)	1.11
Chang (1971)	CHN	Oceania and Asia	1968	0.482	90	7.0	48.8	3.86 (3.12, 4.60)	1.39
Chinnakotla et al. (2023)	ECU	Latin America	2012	0.736	458	3.0	56.4	6.70 (6.20, 7.20)	1.63
Gonçalves et al. (2015)	BRA	Latin America	2011	0.727	205	6.5	51.8	3.11 (2.71, 3.51)	1.73
Guisilini et al. (2021)	BRA	Latin America	2012	0.732	402	2.8	51.7	2.60 (2.25, 2.95)	1.76
Hagens et al. (2023)	PAN	Latin America	2022	0.820	106	9.0	NR	3.80 (3.25, 4.35)	1.58
Hirooka et al. (2014)	BRA	Latin America	2007	0.722	246	4.0	NR	4.70 (4.28, 5.12)	1.71
Kumar et al. (2013)	IND	Oceania and Asia	2012	0.572	743	10.0	51.3	2.79 (2.53, 3.05)	1.83
Lemos et al. (2018)	BRA	Latin America	2007	0.708	100	5.0	52.0	6.43 (5.70, 7.16)	1.40
Lemos et al. (2018)	BRA	Latin America	2013	0.750	86	5.0	61.0	5.85 (5.00, 6.70)	1.28
Miranda et al. (2018)	BRA	Latin America	2010	0.722	52	5.0	52.9	4.02 (2.93, 5.11)	1.06
Oliveira et al. (2024)	BRA	Latin America	2023	0.760	125	3.6	37.6	2.16 (1.73, 2.59)	1.70
Rigonatto et al. (2001)	BRA	Latin America	1991	0.620	171	6.5	54.5	5.52 (4.94, 6.10)	1.56
Sampaio et al. (2010)	BRA	Latin America	2003	0.682	288	3.2	NR	4.18 (3.71, 4.65)	1.67
Singh et al. (2011)	IND	Oceania and Asia	2010	0.572	418	5.0	46.0	4.13 (3.76, 4.50)	1.75
Zeng et al. (2005)	CHN	Oceania and Asia	2004	0.586	470	4.0	NR	3.36 (3.00, 3.72)	1.76
Subgroup, DL (I^2 = 96.5%, p < 0.001)								3.91 (3.37, 4.45)	30.06
0.821-0.890									
Grim et al. (1994)	USA	North America	1989	0.875	457	10.5	48.0	4.52 (4.13, 4.91)	1.74
Hallett et al. (2002)	AUS	Oceania and Asia	2000	0.869	48	5.0	48.0	2.44 (1.27, 3.61)	0.99
Hopcraft et al. (2007)	AUS	Oceania and Asia	2003	0.869	393	8.0	49.0	4.43 (4.04, 4.82)	1.74
Jamieson et al. (2007)	AUS	Oceania and Asia	1994	0.864	450	6.0	48.3	3.09 (2.93, 3.25)	1.88
Jamieson et al. (2007)	AUS	Oceania and Asia	2000	0.869	547	6.0	49.8	3.94 (3.78, 4.10)	1.88
Jamieson et al. (2007)	AUS	Oceania and Asia	1991	0.864	550	6.0	49.7	2.72 (2.58, 2.86)	1.89
Jamieson et al. (2007)	AUS	Oceania and Asia	1989	0.864	196	6.0	50.5	2.85 (2.61, 3.09)	1.84
Jamieson et al. (2007)	AUS	Oceania and Asia	1990	0.864	463	6.0	51.3	2.85 (2.71, 2.99)	1.89
Jamieson et al. (2007)	AUS	Oceania and Asia	1995	0.864	509	6.0	49.3	3.20 (3.05, 3.35)	1.88
Jamieson et al. (2007)	AUS	Oceania and Asia	1992	0.864	465	6.0	50.3	3.09 (2.95, 3.23)	1.89
Jamieson et al. (2007)	AUS	Oceania and Asia	1993	0.864	543	6.0	48.6	2.64 (2.50, 2.78)	1.89
Jamieson et al. (2010)	AUS	Oceania and Asia	1995	0.864	145	7.0	NR	3.40 (2.80, 4.00)	1.54
Jones et al. (1992)	USA	North America	1991	0.875	381	4.0	49.5	4.88 (4.45, 5.31)	1.70
Kailis (1979)	AUS	Oceania and Asia	1971	0.864	201	7.5	52.2	6.33 (5.77, 6.89)	1.58
Kroon et al. (2019)	AUS	Oceania and Asia	2004	0.869	393	8.0	51.5	4.50 (4.05, 4.95)	1.68
Kruger et al. (2005)	AUS	Oceania and Asia	2004	0.869	31	3.5	50.0	4.29 (2.82, 5.76)	0.77
Lawrence (2009)	CAN	North America	2002	0.890	65	4.0	43.0	6.50 (5.15, 7.85)	0.85
Lawrence et al. (2009)	CAN	North America	2003	0.890	76	4.0	46.0	6.70 (5.50, 7.90)	0.96
Lawrence et al. (2009)	CAN	North America	2004	0.890	63	4.0	49.2	5.90 (4.70, 7.10)	0.96
Peressini et al. (2004)	CAN	North America	2000	0.890	87	4.0	54.0	4.20 (3.34, 5.06)	1.27
Peressini et al. (2004)	CAN	North America	2000	0.890	37	7.0	56.7	5.40 (4.40, 6.40)	1.14
Schroth et al. (2005)	CAN	North America	2001	0.890	236	3.0	55.1	4.50 (3.86, 5.14)	1.49
Seow et al. (1996)	AUS	Oceania and Asia	1995	0.864	184	4.8	52.2	3.80 (3.27, 4.33)	1.60
Subgroup, DL (I^2 = 96.5%, p < 0.001)								4.02 (3.71, 4.34)	35.05
> 0.890									
Arrow (2016)	AUS	Oceania and Asia	2014	0.924	268	7.5	47.6	2.54 (2.21, 2.87)	1.78
Batliner et al. (2016)	USA	North America	2015	0.924	50	3.5	46.0	5.70 (4.37, 7.03)	0.87
Butten et al. (2019)	AUS	Oceania and Asia	2014	0.933	111	2.5	48.9	3.00 (2.68, 3.32)	1.79
Dogar et al. (2011)	AUS	Oceania and Asia	2010	0.924	79	3.0	46.0	3.40 (2.58, 4.22)	1.32
Gowda et al. (2009)	NZL	Oceania and Asia	2008	0.924	236	5.5	50.4	4.60 (4.12, 5.08)	1.65
Ha (2014)	AUS	Oceania and Asia	2010	0.924	3985	7.5	NR	3.84 (3.62, 4.06)	1.85
Ha et al. (2014)	AUS	Oceania and Asia	2009	0.924	2918	7.5	NR	3.42 (3.33, 3.51)	1.90
Ha et al. (2016)	AUS	Oceania and Asia	2008	0.924	3251	7.5	NR	2.82 (2.81, 2.83)	1.91
Ha et al. (2016)	AUS	Oceania and Asia	2001	0.896	6918	7.5	NR	1.97 (1.94, 2.00)	1.91
Jamieson et al. (2007)	AUS	Oceania and Asia	1998	0.896	463	6.0	49.4	3.09 (2.92, 3.26)	1.88
Jamieson et al. (2007)	AUS	Oceania and Asia	1996	0.896	574	6.0	51.1	2.79 (2.66, 2.92)	1.89
Jamieson et al. (2007)	AUS	Oceania and Asia	1999	0.896	494	6.0	50.6	3.37 (3.20, 3.54)	1.88
Jamieson et al. (2007)	AUS	Oceania and Asia	1997	0.896	467	6.0	49.1	3.09 (2.93, 3.25)	1.88
Kroon et al. (2019)	AUS	Oceania and Asia	2017	0.933	63	8.0	51.5	3.00 (2.28, 3.72)	1.42
Kroon et al. (2019)	AUS	Oceania and Asia	2012	0.924	263	8.0	51.5	2.80 (2.41, 3.19)	1.74
Kroon et al. (2019)	AUS	Oceania and Asia	2015	0.933	284	8.0	51.5	3.50 (3.12, 3.88)	1.74
Kroon et al. (2019)	AUS	Oceania and Asia	2016	0.933	87	8.0	51.5	2.70 (2.07, 3.33)	1.51
Lee et al. (2022)	CAN	North America	2018	0.928	146	3.0	50.7	4.90 (4.04, 5.76)	1.27
Phipps et al. (2012)	USA	North America	2010	0.916	8323	3.0	52.0	3.50 (3.07, 3.93)	1.70
Smith et al. (2015)	AUS	Oceania and Asia	2014	0.933	173	3.5	45.7	2.40 (1.79, 3.01)	1.53
Tsai et al. (2021)	CAN	North America	2013	0.927	344	2.0	NR	7.00 (6.35, 7.65)	1.49
Subgroup, DL (I^2 = 99.3%, p < 0.001)								3.39 (3.12, 3.65)	34.90
Heterogeneity between groups: p = 0.007									
Overall, DL (I^2 = 98.7%, p < 0.001)								3.75 (3.58, 3.92)	100.00

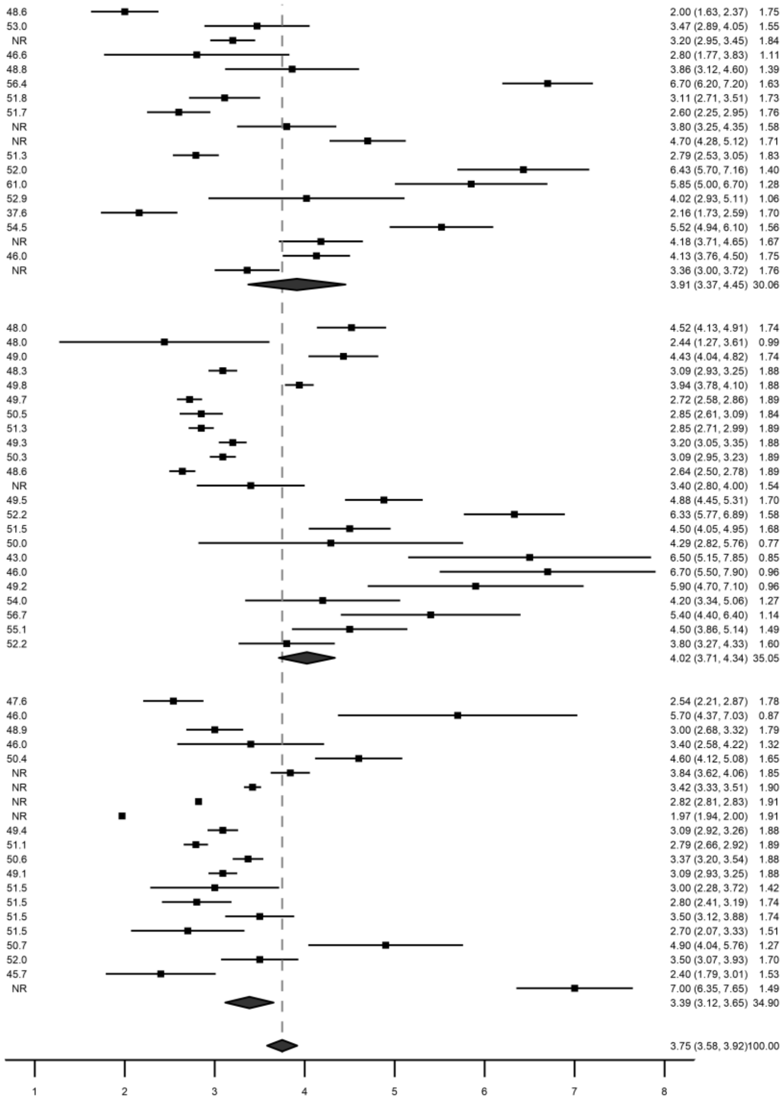

Figure S7. Forest plot of the mean dmft index (deciduous dentition) among Indigenous children by the data collection period.

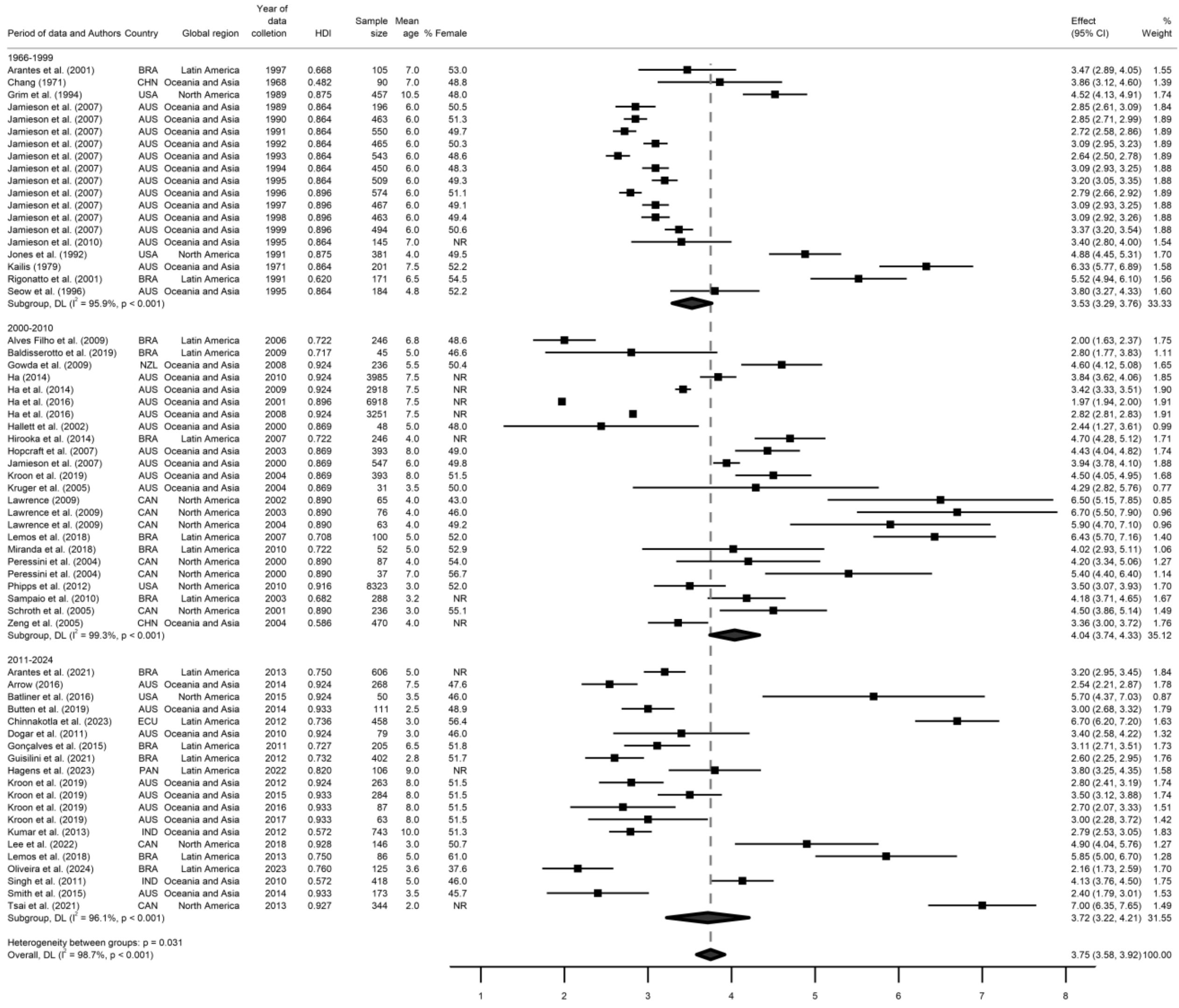

Figure S8. Sensitivity analysis performed by removing studies one by one from the pooled analysis of the dmft index (deciduous dentition) of Indigenous children.

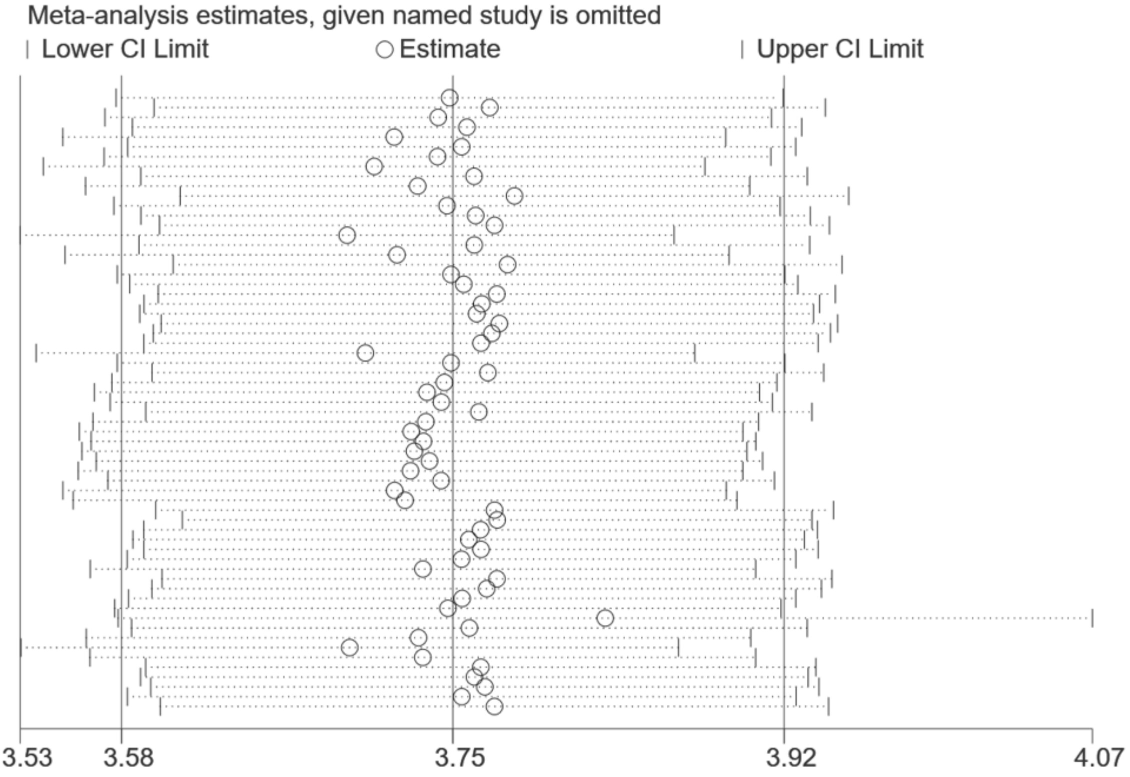

Figure S9. Funnel plot of the dmft index (deciduous dentition) of Indigenous children.

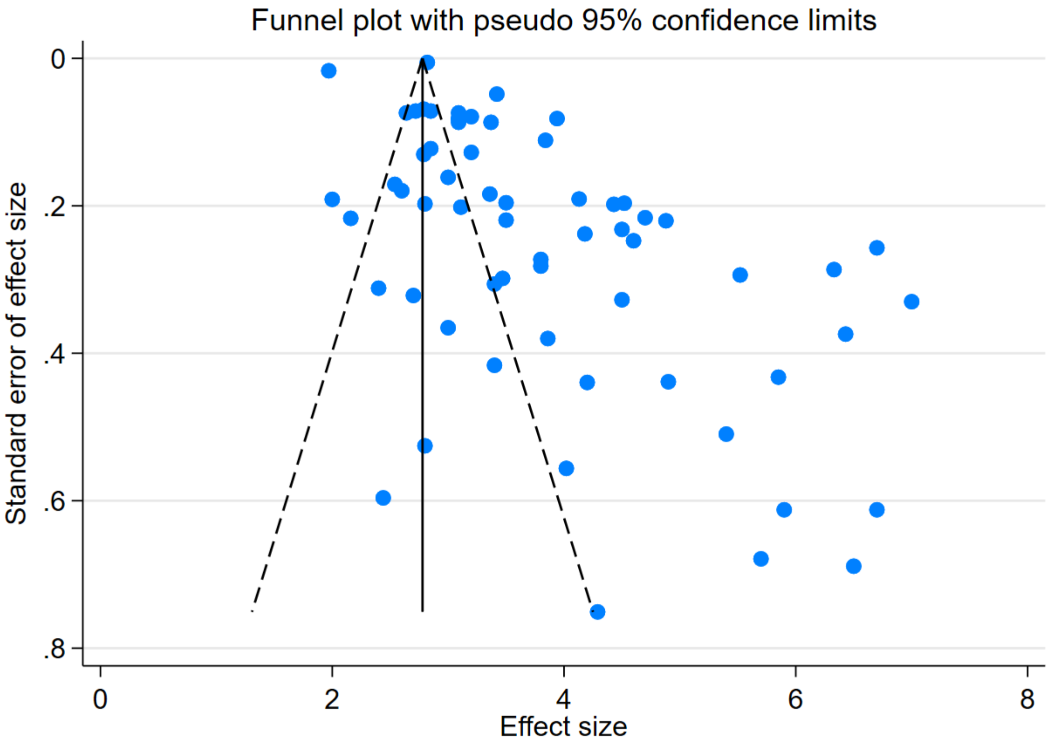

Figure S10. Forest plot of the mean DMFT index (permanent dentition) among Indigenous children and adolescents.

Authors (Year)	Country	Global region	Year of data collection	Num_HDI	Sample size	Mean_age	% Female	Effect (95% CI)	% Weight
Arantes et al. (2009)	BRA	Latin America	1999	.668	33	16	41.2	3.60 (2.34, 4.86)	0.89
Arantes et al. (2009)	BRA	Latin America	1999	.668	82	9	43.3	0.62 (0.37, 0.87)	1.49
Miyazaki et al. (1988)	CHN	Oceania and Asia	1979	.482	295	11	43.7	3.52 (3.26, 3.78)	1.49
Miyazaki et al. (1988)	CHN	Oceania and Asia	1979	.482	548	14	43.7	4.30 (4.02, 4.58)	1.48
Barrett et al. (1972)	AUS	Oceania and Asia	1970	.864	98	14.5	45.2	1.20 (1.12, 1.28)	1.53
Baldisserotto et al. (2019)	BRA	Latin America	2009	.722	74	17	47.3	3.40 (2.73, 4.07)	1.27
Barrett et al. (1972)	AUS	Oceania and Asia	1970	.864	67	7.5	47.4	0.20 (0.10, 0.30)	1.53
Arrow (2016)	AUS	Oceania and Asia	2014	.933	349	10.5	47.6	0.96 (0.77, 1.15)	1.51
Grim et al. (1994)	USA	North America	1989	.875	367	16	48	5.96 (5.55, 6.37)	1.42
Maurício et al. (2014)	BRA	Latin America	2010	.722	88	13.5	48.1	3.00 (2.43, 3.57)	1.34
Maurício et al. (2014)	BRA	Latin America	2010	.722	145	11	48.1	2.00 (1.60, 2.40)	1.43
Jamieson et al. (2007)	AUS	Oceania and Asia	1994	.864	322	12	48.3	1.13 (1.04, 1.22)	1.53
Alves Filho et al. (2009)	BRA	Latin America	2006	.722	73	17	48.6	2.80 (2.32, 3.28)	1.39
Alves Filho et al. (2009)	BRA	Latin America	2006	.722	168	8	48.6	0.86 (0.67, 1.05)	1.51
Jamieson et al. (2007)	AUS	Oceania and Asia	1993	.864	423	12	48.6	0.96 (0.88, 1.04)	1.53
Chang (1971)	CHN	Oceania and Asia	1968	.482	90	7	48.8	0.23 (0.09, 0.37)	1.52
Hopcraft (2007)	AUS	Oceania and Asia	2004	.896	93	14	49	4.49 (3.66, 5.32)	1.17
Hopcraft (2007)	AUS	Oceania and Asia	2004	.896	375	8.5	49	1.47 (1.28, 1.66)	1.51
Batliner et al. (2016)	USA	North America	2015	.924	441	12	49.1	4.83 (4.69, 4.97)	1.52
Jamieson et al. (2007)	AUS	Oceania and Asia	1997	.896	386	12	49.1	0.89 (0.81, 0.97)	1.53
Jamieson et al. (2007)	AUS	Oceania and Asia	1995	.896	498	12	49.3	0.96 (0.90, 1.02)	1.53
Jamieson et al. (2007)	AUS	Oceania and Asia	1998	.896	341	12	49.4	1.25 (1.14, 1.36)	1.52
Jamieson et al. (2007)	AUS	Oceania and Asia	1991	.864	466	12	49.7	1.38 (1.29, 1.47)	1.53
Jamieson et al. (2007)	AUS	Oceania and Asia	2000	.896	442	12	49.8	1.34 (1.24, 1.44)	1.52
Kadir et al. (1990)	MYS	Oceania and Asia	1989	.659	303	10.5	49.8	1.70 (1.51, 1.89)	1.51
Shen et al. (2015)	CHN	Oceania and Asia	2005	.698	360	12	50	0.79 (0.62, 0.96)	1.51
Jamieson et al. (2007)	AUS	Oceania and Asia	1992	.864	444	12	50.3	1.15 (1.06, 1.24)	1.53
Gowda et al. (2009)	NZL	Oceania and Asia	2008	.924	109	12.5	50.4	3.90 (3.30, 4.50)	1.32
Jamieson et al. (2007)	AUS	Oceania and Asia	1989	.864	200	12	50.5	1.26 (1.15, 1.37)	1.52
Jamieson et al. (2007)	AUS	Oceania and Asia	1999	.896	445	12	50.6	1.20 (1.12, 1.28)	1.53
Kailis (1979)	AUS	Oceania and Asia	1971	.864	372	10	50.8	3.88 (3.58, 4.18)	1.47
Jamieson et al. (2007)	AUS	Oceania and Asia	1996	.896	514	12	51.1	0.85 (0.79, 0.91)	1.53
Jamieson et al. (2010)	AUS	Oceania and Asia	2006	.924	442	18	51.1	4.84 (4.39, 5.29)	1.40
Jamieson et al. (2013)	AUS	Oceania and Asia	2007	.924	442	18	51.1	5.12 (4.20, 6.04)	1.11
Kumar et al. (2013)	IND	Oceania and Asia	2012	.572	743	10	51.28	1.43 (1.29, 1.57)	1.52
Arantes et al. (2016)	BRA	Latin America	2013	.752	509	12	51.3	1.52 (1.35, 1.69)	1.51
Jamieson et al. (2007)	AUS	Oceania and Asia	1990	.864	485	12	51.3	1.24 (1.17, 1.31)	1.53
Lemos et al. (2018)	BRA	Latin America	2007	.722	218	17	51.4	6.89 (6.34, 7.44)	1.35
Kroon et al. (2019)	AUS	Oceania and Asia	2004	.896	468	10	51.5	2.00 (1.74, 2.26)	1.49
Kroon et al. (2019)	AUS	Oceania and Asia	2012	.924	309	10	51.5	1.30 (1.16, 1.44)	1.52
Kroon et al. (2019)	AUS	Oceania and Asia	2015	.933	333	10	51.5	1.14 (0.88, 1.40)	1.49
Kroon et al. (2019)	AUS	Oceania and Asia	2016	.933	120	10	51.5	1.00 (0.62, 1.38)	1.44
Kroon et al. (2019)	AUS	Oceania and Asia	2017	.941	114	10	51.5	1.30 (0.97, 1.63)	1.46
Gonçalves et al. (2015)	BRA	Latin America	2011	.722	305	10	51.8	2.21 (1.93, 2.49)	1.48
Lemos et al. (2018)	BRA	Latin America	2013	.752	77	12	51.9	2.78 (2.20, 3.36)	1.33
Miranda et al. (2018)	BRA	Latin America	2010	.722	48	17	52.9	3.21 (2.23, 4.19)	1.07
Miranda et al. (2018)	BRA	Latin America	2010	.722	113	8.5	52.9	1.09 (0.70, 1.47)	1.44
Arantes et al. (2001)	BRA	Latin America	1997	.62	60	9	53	1.08 (0.60, 1.56)	1.39
Arantes et al. (2001)	BRA	Latin America	1997	.668	37	16	53	4.54 (3.32, 5.76)	0.91
John et al. (2015)	IND	Oceania and Asia	2014	.619	206	10.5	53.9	2.88 (2.62, 3.14)	1.49
Lemos et al. (2018)	BRA	Latin America	2007	.722	50	12	54	2.54 (1.94, 3.14)	1.32
Rigonatto et al. (2001)	BRA	Latin America	1991	.62	130	9	54.5	3.05 (2.56, 3.54)	1.38
Rigonatto et al. (2001)	BRA	Latin America	1991	.62	37	17	54.5	9.14 (6.18, 12.10)	0.31
Lemos et al. (2018)	BRA	Latin America	2013	.752	260	17	57.3	4.65 (4.27, 5.03)	1.44
Peressini et al. (2004)	CAN	North America	2000	.89	29	13	65.5	4.10 (3.15, 5.05)	1.09
Baldisserotto et al. (2019)	BRA	Latin America	2009	.722	35	12	65.7	1.30 (0.85, 1.75)	1.40
Quintero De La Hoz (2022)	VEN	Latin America	2016	.766	25	14.5	100	5.52 (3.80, 7.24)	0.65
Arantes et al. (2021)	BRA	Latin America	2013	.752	415	17	NR	3.29 (3.00, 3.58)	1.48
Arantes et al. (2021)	BRA	Latin America	2013	.752	543	12	NR	1.52 (1.35, 1.69)	1.51
Ha (2014)	AUS	Oceania and Asia	2010	.924	10016	10.5	NR	1.83 (1.65, 2.01)	1.51
Ha et al. (2014)	AUS	Oceania and Asia	2009	.924	2311	10	NR	1.20 (1.16, 1.24)	1.53
Ha et al. (2016)	AUS	Oceania and Asia	2001	.896	9674	10.5	NR	0.76 (0.74, 0.78)	1.53
Ha et al. (2016)	AUS	Oceania and Asia	2008	.924	5094	10.5	NR	1.41 (1.40, 1.42)	1.53
Hagens et al. (2023)	PAN	Latin America	2022	.82	106	9	NR	0.60 (0.39, 0.81)	1.50
Jamieson et al. (2010)	AUS	Oceania and Asia	1995	.896	145	7	NR	0.30 (0.10, 0.50)	1.50
Jamieson et al. (2013)	AUS	Oceania and Asia	2006	.924	441	18	NR	4.83 (4.69, 4.97)	1.52
Medina et al. (2008)	ECU	Latin America	1998	.684	930	9	NR	2.95 (2.78, 3.12)	1.51
Niendorff et al. (2000)	USA	North America	1991	.875	3290	16.5	NR	7.00 (6.84, 7.16)	1.51
Niendorff et al.(2000)	USA	North America	1991	.875	6424	9	NR	1.80 (1.74, 1.86)	1.53
Sampaio et al (2010)	BRA	Latin America	2003	.668	507	17	NR	7.13 (6.70, 7.56)	1.41
Sampaio et al (2010)	BRA	Latin America	2003	.668	159	12	NR	3.68 (3.19, 4.17)	1.38
Overall, DL (I^2 = 99.6%, $p < 0.001$)								2.36 (2.18, 2.55)	100.00

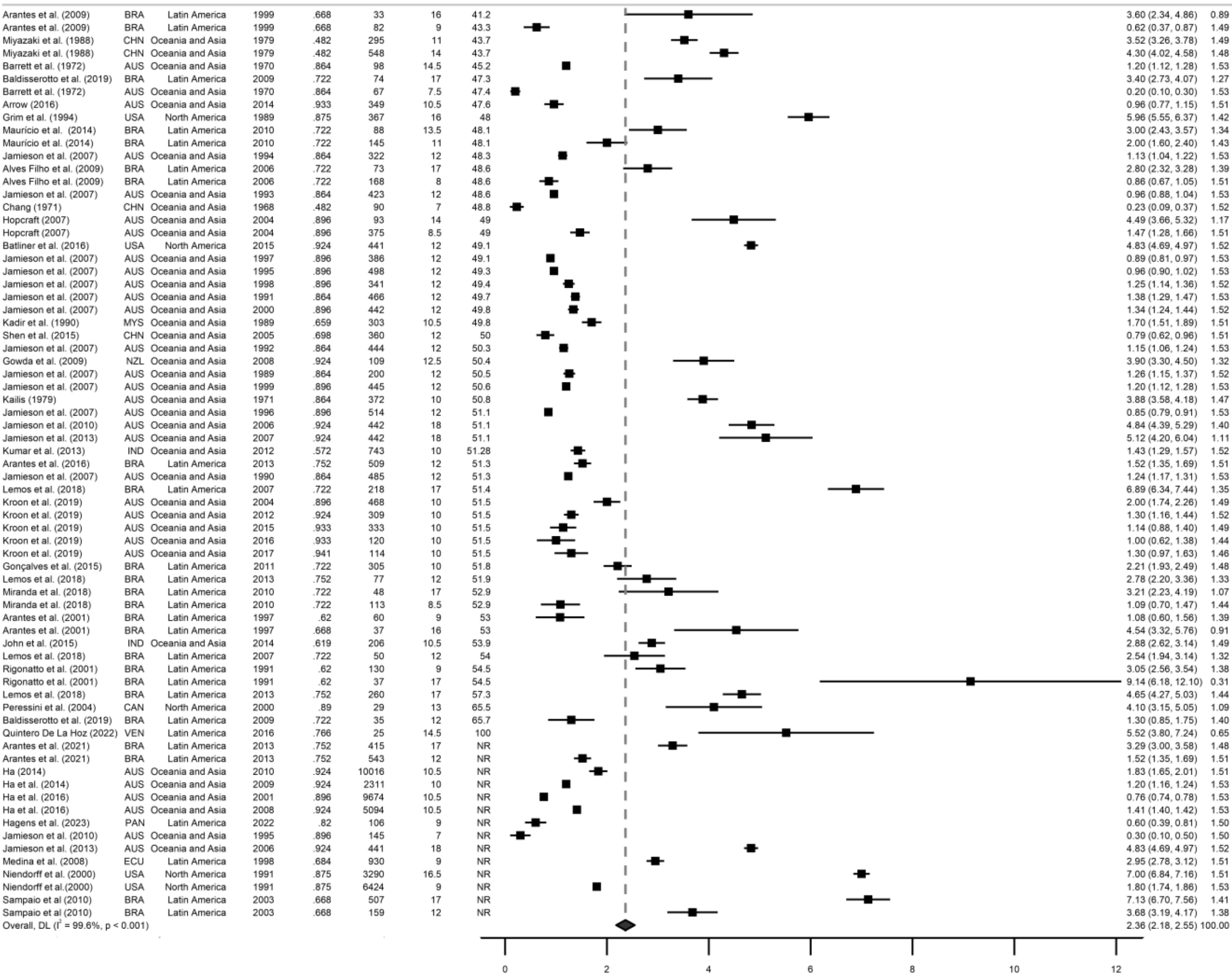

Figure S11. Forest plot of the mean DMFT index (permanent dentition) among Indigenous children and adolescents by age group.

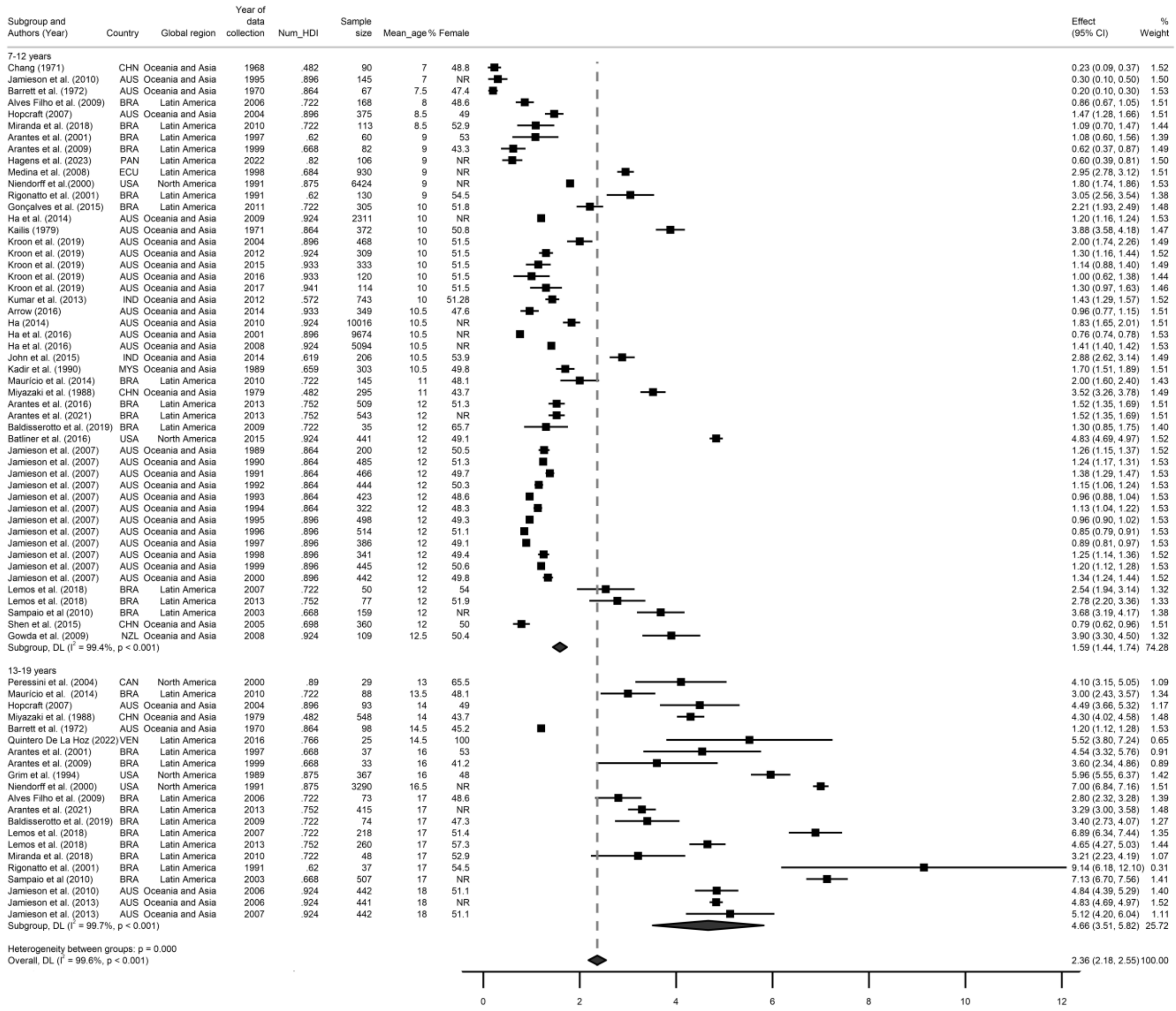

Figure S12. Forest plot of the mean DMFT index (permanent dentition) among Indigenous children and adolescents by the percentage of female individuals.

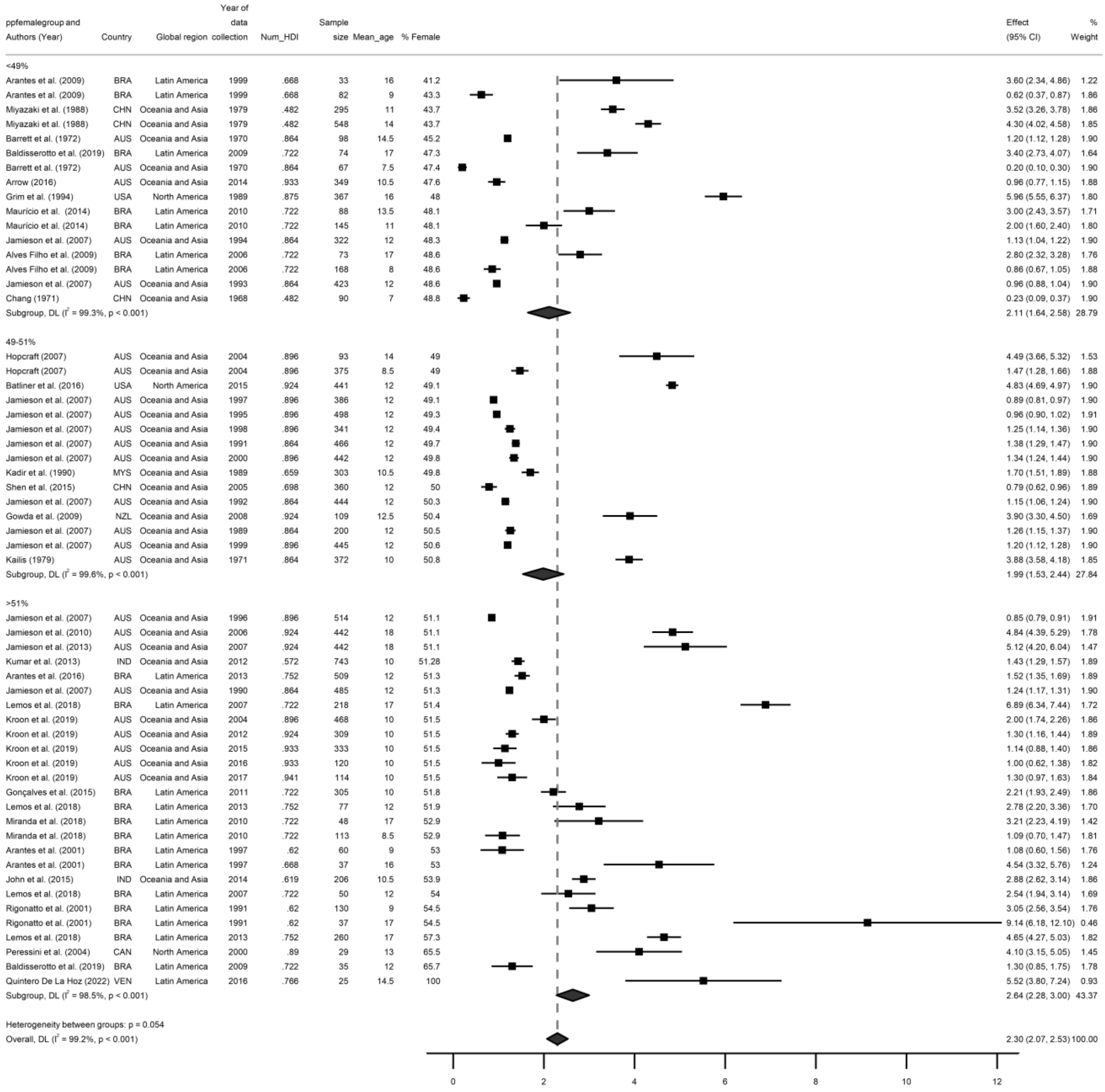

Figure S13. Forest plot of the mean DMFT index (permanent dentition) among Indigenous children and adolescents by country.

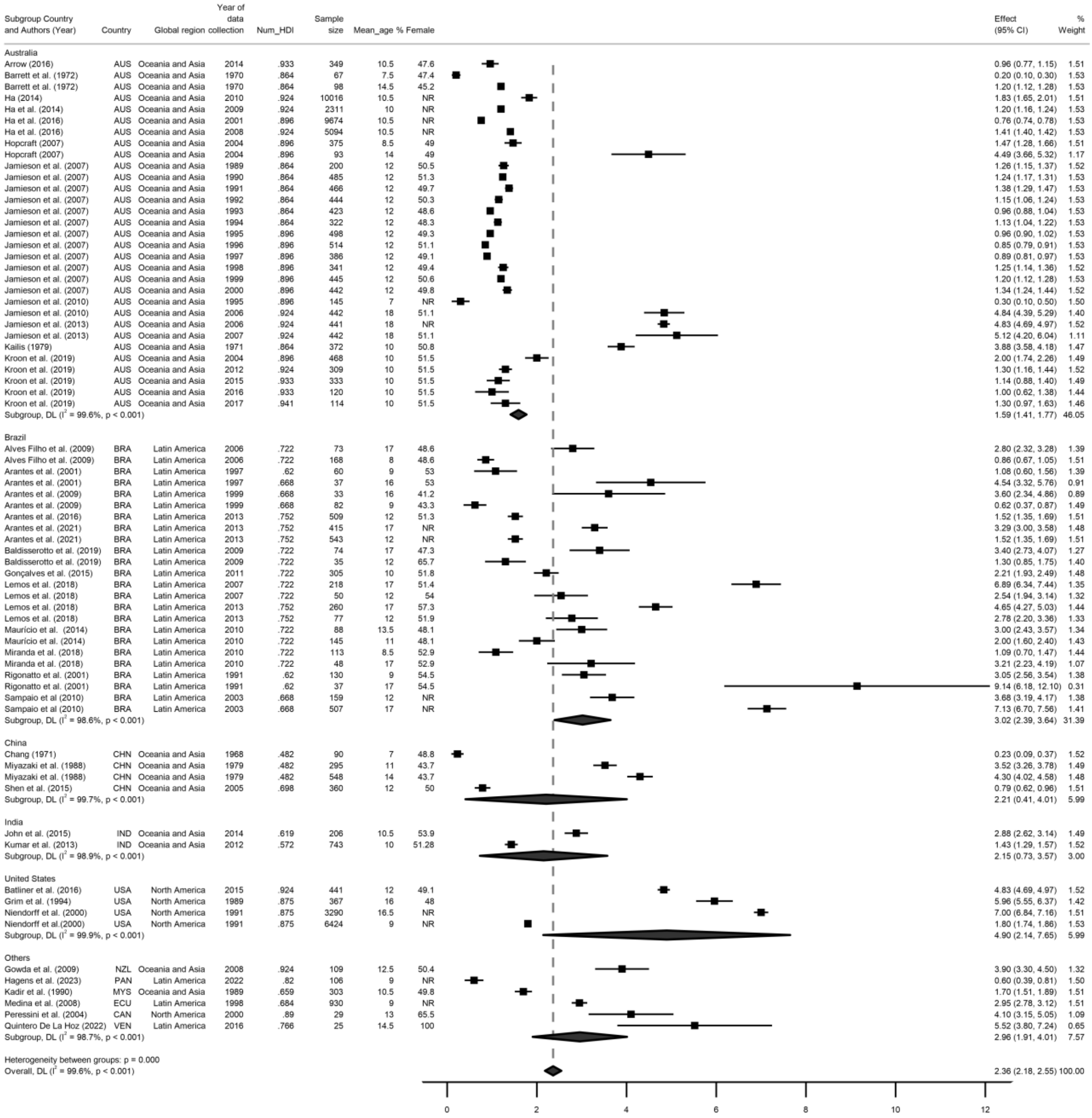

Figure S14. Forest plot of the mean DMFT index (permanent dentition) among Indigenous children and adolescents by global region.

Global region and Authors (Year)	Country	Year of data collection	Num_HDI	Sample size	Mean_age	% Female	Effect (95% CI)	% Weight
North America								
Batliner et al. (2016)	USA	2015	.924	441	12	49.1	4.83 (4.69, 4.97)	1.52
Grim et al. (1994)	USA	1989	.875	367	16	48	5.96 (5.55, 6.37)	1.42
Niendorff et al. (2000)	USA	1991	.875	3290	16.5	NR	7.00 (6.84, 7.16)	1.51
Niendorff et al.(2000)	USA	1991	.875	6424	9	NR	1.80 (1.74, 1.86)	1.53
Peressini et al. (2004)	CAN	2000	.89	29	13	65.5	4.10 (3.15, 5.05)	1.09
Subgroup, DL (I^2 = 99.9%, p < 0.001)							4.74 (2.28, 7.20)	7.07
Latin America								
Alves Filho et al. (2009)	BRA	2006	.722	168	8	48.6	0.86 (0.67, 1.05)	1.51
Alves Filho et al. (2009)	BRA	2006	.722	73	17	48.6	2.80 (2.32, 3.28)	1.39
Arantes et al. (2001)	BRA	1997	.668	37	16	53	4.54 (3.32, 5.76)	0.91
Arantes et al. (2001)	BRA	1997	.62	60	9	53	1.08 (0.60, 1.56)	1.39
Arantes et al. (2009)	BRA	1999	.668	33	16	41.2	3.60 (2.34, 4.86)	0.89
Arantes et al. (2009)	BRA	1999	.668	82	9	43.3	0.62 (0.37, 0.87)	1.49
Arantes et al. (2016)	BRA	2013	.752	509	12	51.3	1.52 (1.35, 1.69)	1.51
Arantes et al. (2021)	BRA	2013	.752	543	12	NR	1.52 (1.35, 1.69)	1.51
Arantes et al. (2021)	BRA	2013	.752	415	17	NR	3.29 (3.00, 3.58)	1.48
Baldisserotto et al. (2019)	BRA	2009	.722	35	12	65.7	1.30 (0.85, 1.75)	1.40
Baldisserotto et al. (2019)	BRA	2009	.722	74	17	47.3	3.40 (2.73, 4.07)	1.27
Gonçalves et al. (2015)	BRA	2011	.722	305	10	51.8	2.21 (1.93, 2.49)	1.48
Hagens et al. (2023)	PAN	2022	.82	106	9	NR	0.60 (0.39, 0.81)	1.50
Lemos et al. (2018)	BRA	2007	.722	50	12	54	2.54 (1.94, 3.14)	1.32
Lemos et al. (2018)	BRA	2007	.722	218	17	51.4	6.89 (6.34, 7.44)	1.35
Lemos et al. (2018)	BRA	2013	.752	260	17	57.3	4.65 (4.27, 5.03)	1.44
Lemos et al. (2018)	BRA	2013	.752	77	12	51.9	2.78 (2.20, 3.36)	1.33
Maurício et al. (2014)	BRA	2010	.722	88	13.5	48.1	3.00 (2.43, 3.57)	1.34
Maurício et al. (2014)	BRA	2010	.722	145	11	48.1	2.00 (1.60, 2.40)	1.43
Medina et al. (2008)	ECU	1998	.684	930	9	NR	2.95 (2.78, 3.12)	1.51
Miranda et al. (2018)	BRA	2010	.722	113	8.5	52.9	1.09 (0.70, 1.47)	1.44
Miranda et al. (2018)	BRA	2010	.722	48	17	52.9	3.21 (2.23, 4.19)	1.07
Quintero De La Hoz (2022)	VEN	2016	.766	25	14.5	100	5.52 (3.80, 7.24)	0.65
Rigonatto et al. (2001)	BRA	1991	.62	130	9	54.5	3.05 (2.56, 3.54)	1.38
Rigonatto et al. (2001)	BRA	1991	.62	37	17	54.5	9.14 (6.18, 12.10)	0.31
Sampaio et al (2010)	BRA	2003	.668	507	17	NR	7.13 (6.70, 7.56)	1.41
Sampaio et al (2010)	BRA	2003	.668	159	12	NR	3.68 (3.19, 4.17)	1.38
Subgroup, DL (I^2 = 98.7%, p < 0.001)							2.99 (2.42, 3.55)	35.06
Oceania and Asia								
Arrow (2016)	AUS	2014	.933	349	10.5	47.6	0.96 (0.77, 1.15)	1.51
Barrett et al. (1972)	AUS	1970	.864	67	7.5	47.4	0.20 (0.10, 0.30)	1.53
Barrett et al. (1972)	AUS	1970	.864	98	14.5	45.2	1.20 (1.12, 1.28)	1.53
Chang (1971)	CHN	1968	.482	90	7	48.8	0.23 (0.09, 0.37)	1.52
Gowda et al. (2009)	NZL	2008	.924	109	12.5	50.4	3.90 (3.30, 4.50)	1.32
Ha (2014)	AUS	2010	.924	10016	10.5	NR	1.83 (1.65, 2.01)	1.51
Ha et al. (2014)	AUS	2009	.924	2311	10	NR	1.20 (1.16, 1.24)	1.53
Ha et al. (2016)	AUS	2001	.896	9674	10.5	NR	0.76 (0.74, 0.78)	1.53
Ha et al. (2016)	AUS	2008	.924	5094	10.5	NR	1.41 (1.40, 1.42)	1.53
Hopcraft (2007)	AUS	2004	.896	93	14	49	4.49 (3.66, 5.32)	1.17
Hopcraft (2007)	AUS	2004	.896	375	8.5	49	1.47 (1.28, 1.66)	1.51
Jamieson et al. (2007)	AUS	1989	.864	200	12	50.5	1.26 (1.15, 1.37)	1.52
Jamieson et al. (2007)	AUS	1990	.864	485	12	51.3	1.24 (1.17, 1.31)	1.53
Jamieson et al. (2007)	AUS	1991	.864	466	12	49.7	1.38 (1.29, 1.47)	1.53
Jamieson et al. (2007)	AUS	1992	.864	444	12	50.3	1.15 (1.06, 1.24)	1.53
Jamieson et al. (2007)	AUS	1993	.864	423	12	48.6	0.96 (0.88, 1.04)	1.53
Jamieson et al. (2007)	AUS	1994	.864	322	12	48.3	1.13 (1.04, 1.22)	1.53
Jamieson et al. (2007)	AUS	1995	.896	498	12	49.3	0.96 (0.90, 1.02)	1.53
Jamieson et al. (2007)	AUS	1996	.896	514	12	51.1	0.85 (0.79, 0.91)	1.53
Jamieson et al. (2007)	AUS	1997	.896	386	12	49.1	0.89 (0.81, 0.97)	1.53
Jamieson et al. (2007)	AUS	1998	.896	341	12	49.4	1.25 (1.14, 1.36)	1.52
Jamieson et al. (2007)	AUS	1999	.896	445	12	50.6	1.20 (1.12, 1.28)	1.53
Jamieson et al. (2007)	AUS	2000	.896	442	12	49.8	1.34 (1.24, 1.44)	1.52
Jamieson et al. (2010)	AUS	1995	.896	145	7	NR	0.30 (0.10, 0.50)	1.50
Jamieson et al. (2010)	AUS	2006	.924	442	18	51.1	4.84 (4.39, 5.29)	1.40
Jamieson et al. (2013)	AUS	2006	.924	441	18	NR	4.83 (4.69, 4.97)	1.52
Jamieson et al. (2013)	AUS	2007	.924	442	18	51.1	5.12 (4.20, 6.04)	1.11
John et al. (2015)	IND	2014	.619	206	10.5	53.9	2.88 (2.62, 3.14)	1.49
Kadir et al. (1990)	MYS	1989	.659	303	10.5	49.8	1.70 (1.51, 1.89)	1.51
Kailis (1979)	AUS	1971	.864	372	10	50.8	3.88 (3.58, 4.18)	1.47
Kroon et al. (2019)	AUS	2004	.896	468	10	51.5	2.00 (1.74, 2.26)	1.49
Kroon et al. (2019)	AUS	2012	.924	309	10	51.5	1.30 (1.16, 1.44)	1.52
Kroon et al. (2019)	AUS	2015	.933	333	10	51.5	1.14 (0.88, 1.40)	1.49
Kroon et al. (2019)	AUS	2016	.933	120	10	51.5	1.00 (0.62, 1.38)	1.44
Kroon et al. (2019)	AUS	2017	.941	114	10	51.5	1.30 (0.97, 1.63)	1.46
Kumar et al. (2013)	IND	2012	.572	743	10	51.28	1.43 (1.29, 1.57)	1.52
Miyazaki et al. (1988)	CHN	1979	.482	548	14	43.7	4.30 (4.02, 4.58)	1.48
Miyazaki et al. (1988)	CHN	1979	.482	295	11	43.7	3.52 (3.26, 3.78)	1.49
Shen et al. (2015)	CHN	2005	.698	360	12	50	0.79 (0.62, 0.96)	1.51
Subgroup, DL (I^2 = 99.5%, p < 0.001)							1.74 (1.57, 1.91)	57.87
Heterogeneity between groups: p = 0.000								
Overall, DL (I^2 = 99.6%, p < 0.001)							2.36 (2.18, 2.55)	100.00

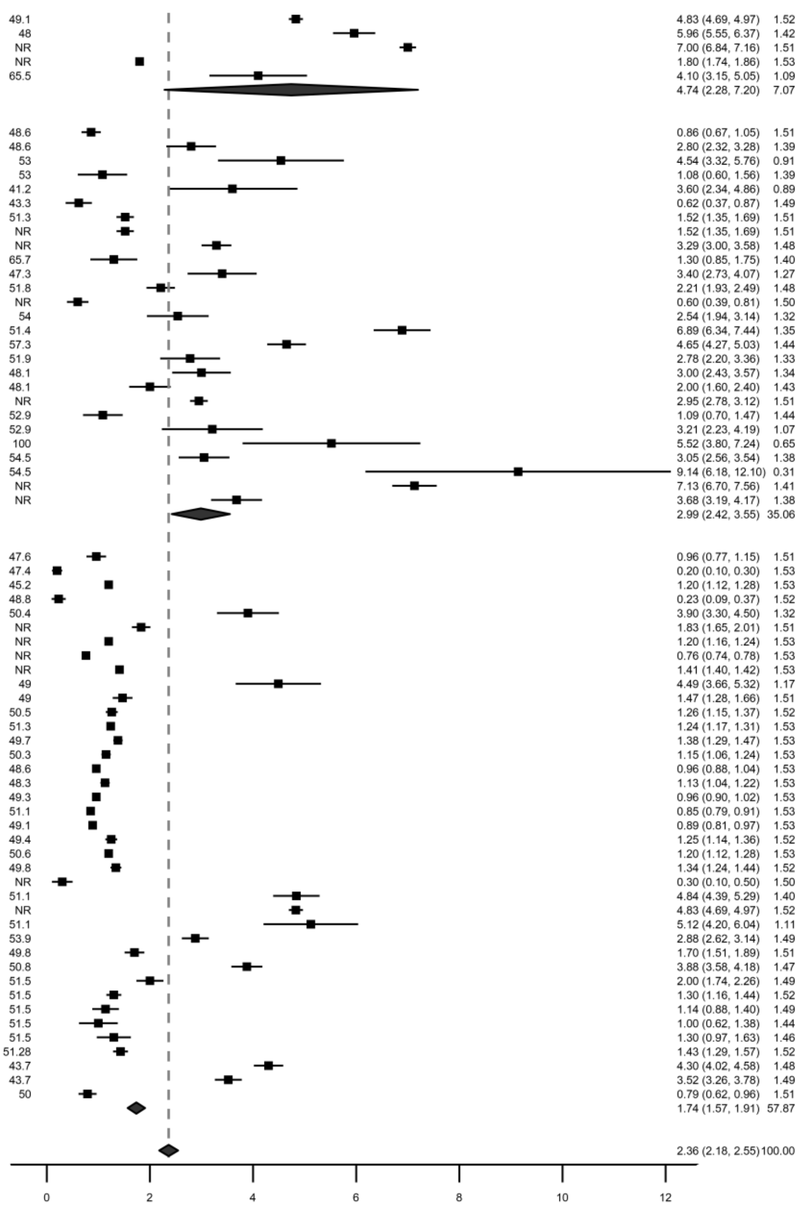

Figure S15. Forest plot of the mean DMFT index (permanent dentition) among Indigenous children and adolescents by HDI.

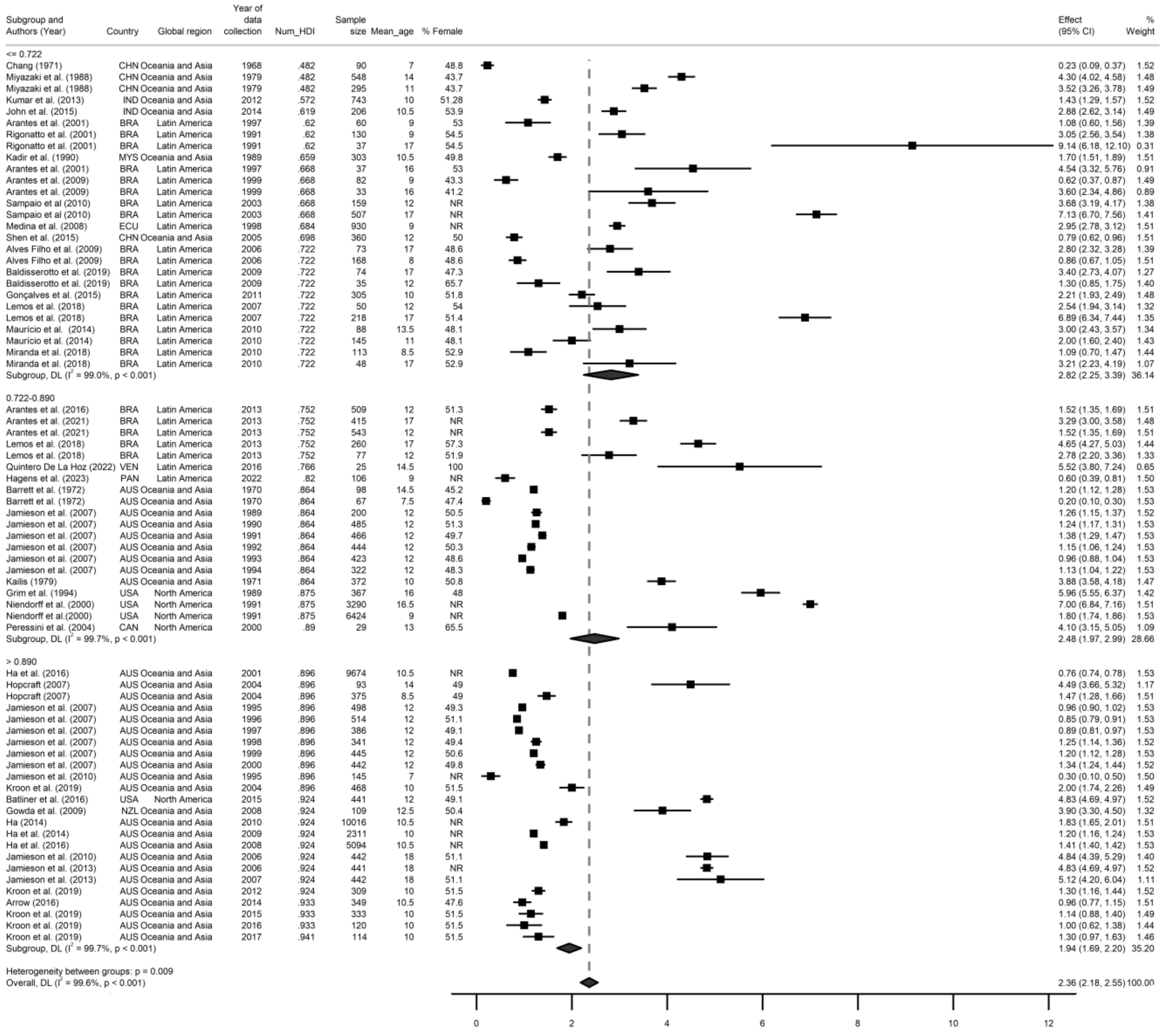

Figure S16. Forest plot of the mean DMFT index (permanent dentition) among Indigenous children and adolescents by the data collection period.

Period of data and Authors (Year)	Country	Global region	Year of data collection	Num_HDI	Sample size	Mean_age	% Female	Effect (95% CI)	% Weight
1968-1999									
Arantes et al. (2001)	BRA	Latin America	1997	.62	60	9	53	1.08 (0.60, 1.56)	1.39
Arantes et al. (2001)	BRA	Latin America	1997	.668	37	16	53	4.54 (3.32, 5.76)	0.91
Arantes et al. (2009)	BRA	Latin America	1999	.668	33	16	41.2	3.60 (2.34, 4.86)	0.89
Arantes et al. (2009)	BRA	Latin America	1999	.668	82	9	43.3	0.62 (0.37, 0.87)	1.49
Barrett et al. (1972)	AUS	Oceania and Asia	1970	.864	67	7.5	47.4	0.20 (0.10, 0.30)	1.53
Barrett et al. (1972)	AUS	Oceania and Asia	1970	.864	98	14.5	45.2	1.20 (1.12, 1.28)	1.53
Chang (1971)	CHN	Oceania and Asia	1968	.482	90	7	48.8	0.23 (0.09, 0.37)	1.52
Grim et al. (1994)	USA	North America	1989	.875	367	16	48	5.96 (5.55, 6.37)	1.42
Jamieson et al. (2007)	AUS	Oceania and Asia	1989	.864	200	12	50.5	1.26 (1.15, 1.37)	1.52
Jamieson et al. (2007)	AUS	Oceania and Asia	1990	.864	485	12	51.3	1.24 (1.17, 1.31)	1.53
Jamieson et al. (2007)	AUS	Oceania and Asia	1991	.864	466	12	49.7	1.38 (1.29, 1.47)	1.53
Jamieson et al. (2007)	AUS	Oceania and Asia	1992	.864	444	12	50.3	1.15 (1.06, 1.24)	1.53
Jamieson et al. (2007)	AUS	Oceania and Asia	1993	.864	423	12	48.6	0.96 (0.88, 1.04)	1.53
Jamieson et al. (2007)	AUS	Oceania and Asia	1994	.864	322	12	48.3	1.13 (1.04, 1.22)	1.53
Jamieson et al. (2007)	AUS	Oceania and Asia	1995	.896	498	12	49.3	0.96 (0.90, 1.02)	1.53
Jamieson et al. (2007)	AUS	Oceania and Asia	1996	.896	514	12	51.1	0.85 (0.79, 0.91)	1.53
Jamieson et al. (2007)	AUS	Oceania and Asia	1997	.896	386	12	49.1	0.89 (0.81, 0.97)	1.53
Jamieson et al. (2007)	AUS	Oceania and Asia	1998	.896	341	12	49.4	1.25 (1.14, 1.36)	1.52
Jamieson et al. (2007)	AUS	Oceania and Asia	1999	.896	445	12	50.6	1.20 (1.12, 1.28)	1.53
Jamieson et al. (2010)	AUS	Oceania and Asia	1995	.896	145	7	NR	0.30 (0.10, 0.50)	1.50
Kadir et al. (1990)	MYS	Oceania and Asia	1989	.659	303	10.5	49.8	1.70 (1.51, 1.89)	1.51
Kailis (1979)	AUS	Oceania and Asia	1971	.864	372	10	50.8	3.88 (3.58, 4.18)	1.47
Medina et al. (2008)	ECU	Latin America	1998	.684	930	9	NR	2.95 (2.78, 3.12)	1.51
Miyazaki et al. (1988)	CHN	Oceania and Asia	1979	.482	295	11	43.7	3.52 (3.26, 3.78)	1.49
Miyazaki et al. (1988)	CHN	Oceania and Asia	1979	.482	548	14	43.7	4.30 (4.02, 4.58)	1.48
Niendorff et al. (2000)	USA	North America	1991	.875	3290	16.5	NR	7.00 (6.84, 7.16)	1.51
Niendorff et al.(2000)	USA	North America	1991	.875	6424	9	NR	1.80 (1.74, 1.86)	1.53
Rigonatto et al. (2001)	BRA	Latin America	1991	.62	37	17	54.5	9.14 (6.18, 12.10)	0.31
Rigonatto et al. (2001)	BRA	Latin America	1991	.62	130	9	54.5	3.05 (2.56, 3.54)	1.38
Subgroup, DL (I^2 = 99.7%, p < 0.001)								2.10 (1.74, 2.46)	41.15
2000-2010									
Alves Filho et al. (2009)	BRA	Latin America	2006	.722	73	17	48.6	2.80 (2.32, 3.28)	1.39
Alves Filho et al. (2009)	BRA	Latin America	2006	.722	168	8	48.6	0.86 (0.67, 1.05)	1.51
Baldisserotto et al. (2019)	BRA	Latin America	2009	.722	74	17	47.3	3.40 (2.73, 4.07)	1.27
Baldisserotto et al. (2019)	BRA	Latin America	2009	.722	35	12	65.7	1.30 (0.85, 1.75)	1.40
Gowda et al. (2009)	NZL	Oceania and Asia	2008	.924	109	12.5	50.4	3.90 (3.30, 4.50)	1.32
Ha (2014)	AUS	Oceania and Asia	2010	.924	10016	10.5	NR	1.83 (1.65, 2.01)	1.51
Ha et al. (2014)	AUS	Oceania and Asia	2009	.924	2311	10	NR	1.20 (1.16, 1.24)	1.53
Ha et al. (2016)	AUS	Oceania and Asia	2001	.896	9674	10.5	NR	0.76 (0.74, 0.78)	1.53
Ha et al. (2016)	AUS	Oceania and Asia	2008	.924	5094	10.5	NR	1.41 (1.40, 1.42)	1.53
Hopcraft (2007)	AUS	Oceania and Asia	2004	.896	375	8.5	49	1.47 (1.28, 1.66)	1.51
Hopcraft (2007)	AUS	Oceania and Asia	2004	.896	93	14	49	4.49 (3.66, 5.32)	1.17
Jamieson et al. (2007)	AUS	Oceania and Asia	2000	.896	442	12	49.8	1.34 (1.24, 1.44)	1.52
Jamieson et al. (2010)	AUS	Oceania and Asia	2006	.924	442	18	51.1	4.84 (4.39, 5.29)	1.40
Jamieson et al. (2013)	AUS	Oceania and Asia	2006	.924	441	18	NR	4.83 (4.69, 4.97)	1.52
Jamieson et al. (2013)	AUS	Oceania and Asia	2007	.924	442	18	51.1	5.12 (4.20, 6.04)	1.11
Kroon et al. (2019)	AUS	Oceania and Asia	2004	.896	468	10	51.5	2.00 (1.74, 2.26)	1.49
Lemos et al. (2018)	BRA	Latin America	2007	.722	50	12	54	2.54 (1.94, 3.14)	1.32
Lemos et al. (2018)	BRA	Latin America	2007	.722	218	17	51.4	6.89 (6.34, 7.44)	1.35
Maurício et al. (2014)	BRA	Latin America	2010	.722	88	13.5	48.1	3.00 (2.43, 3.57)	1.34
Maurício et al. (2014)	BRA	Latin America	2010	.722	145	11	48.1	2.00 (1.60, 2.40)	1.43
Miranda et al. (2018)	BRA	Latin America	2010	.722	113	8.5	52.9	1.09 (0.70, 1.47)	1.44
Miranda et al. (2018)	BRA	Latin America	2010	.722	48	17	52.9	3.21 (2.23, 4.19)	1.07
Peressini et al. (2004)	CAN	North America	2000	.89	29	13	65.5	4.10 (3.15, 5.05)	1.09
Sampaio et al (2010)	BRA	Latin America	2003	.668	159	12	NR	3.68 (3.19, 4.17)	1.38
Sampaio et al (2010)	BRA	Latin America	2003	.668	507	17	NR	7.13 (6.70, 7.56)	1.41
Shen et al. (2015)	CHN	Oceania and Asia	2005	.698	360	12	50	0.79 (0.62, 0.96)	1.51
Subgroup, DL (I^2 = 99.7%, p < 0.001)								2.80 (2.54, 3.06)	36.01
2011-2024									
Arantes et al. (2016)	BRA	Latin America	2013	.752	509	12	51.3	1.52 (1.35, 1.69)	1.51
Arantes et al. (2021)	BRA	Latin America	2013	.752	543	12	NR	1.52 (1.35, 1.69)	1.51
Arantes et al. (2021)	BRA	Latin America	2013	.752	415	17	NR	3.29 (3.00, 3.58)	1.48
Arrow (2016)	AUS	Oceania and Asia	2014	.933	349	10.5	47.6	0.96 (0.77, 1.15)	1.51
Batliner et al. (2016)	USA	North America	2015	.924	441	12	49.1	4.83 (4.69, 4.97)	1.52
Gonçalves et al. (2015)	BRA	Latin America	2011	.722	305	10	51.8	2.21 (1.93, 2.49)	1.48
Hagens et al. (2023)	PAN	Latin America	2022	.82	106	9	NR	0.60 (0.39, 0.81)	1.50
John et al. (2015)	IND	Oceania and Asia	2014	.619	206	10.5	53.9	2.88 (2.62, 3.14)	1.49
Kroon et al. (2019)	AUS	Oceania and Asia	2012	.924	309	10	51.5	1.30 (1.16, 1.44)	1.52
Kroon et al. (2019)	AUS	Oceania and Asia	2015	.933	333	10	51.5	1.14 (0.88, 1.40)	1.49
Kroon et al. (2019)	AUS	Oceania and Asia	2016	.933	120	10	51.5	1.00 (0.62, 1.38)	1.44
Kroon et al. (2019)	AUS	Oceania and Asia	2017	.941	114	10	51.5	1.30 (0.97, 1.63)	1.46
Kumar et al. (2013)	IND	Oceania and Asia	2012	.572	743	10	51.28	1.43 (1.29, 1.57)	1.52
Lemos et al. (2018)	BRA	Latin America	2013	.752	260	17	57.3	4.65 (4.27, 5.03)	1.44
Lemos et al. (2018)	BRA	Latin America	2013	.752	77	12	51.9	2.78 (2.20, 3.36)	1.33
Quintero De La Hoz (2022)	VEN	Latin America	2016	.766	25	14.5	100	5.52 (3.80, 7.24)	0.65
Subgroup, DL (I^2 = 99.4%, p < 0.001)								2.25 (1.53, 2.97)	22.83
Heterogeneity between groups: p = 0.007									
Overall, DL (I^2 = 99.6%, p < 0.001)								2.36 (2.18, 2.55)	100.00

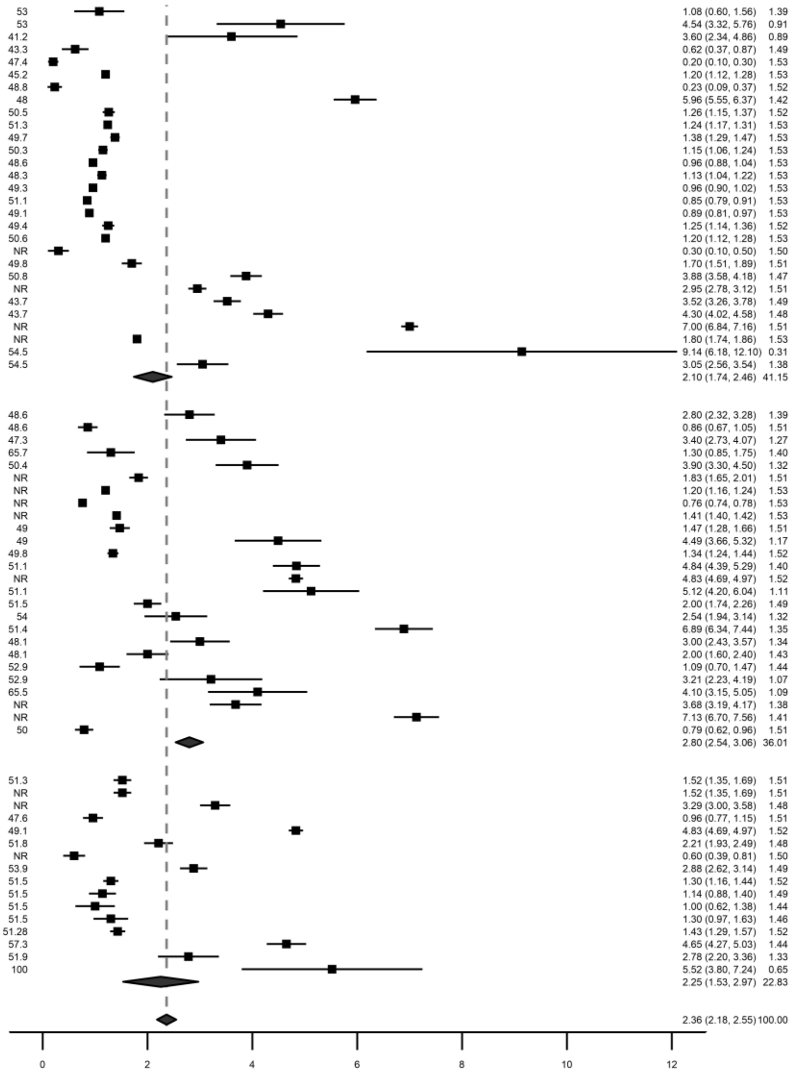

Figure S17. Sensitivity analysis performed by removing studies one by one from the pooled analysis of the DMFT index (permanent dentition) of Indigenous children and adolescents.

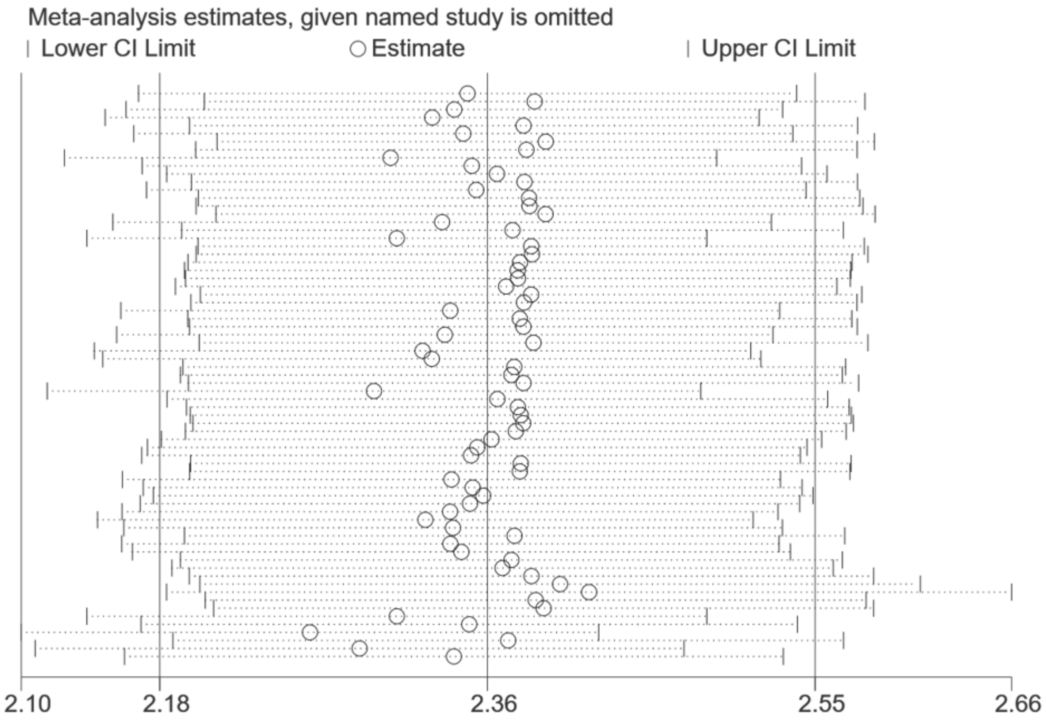

Figure S18. Funnel plot of the DMFT index (permanent dentition) of Indigenous children and adolescents.

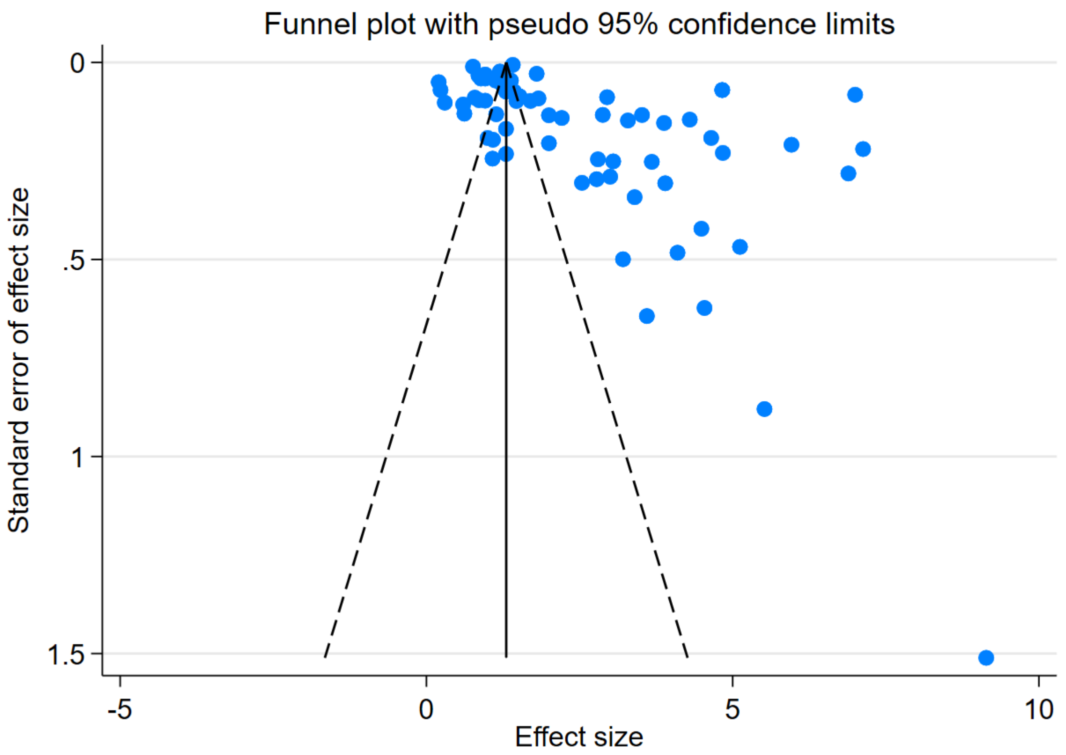

Supplement: Supplementary file 1 — Supplementary Material 1 (PDF 6.44 MB) [file 784_2026_7047_MOESM1_ESM.pdf]
